# Diagnostic accuracy of deep learning in medical imaging: a systematic review and meta-analysis

**DOI:** 10.1038/s41746-021-00438-z

**Published:** 2021-04-07

**Authors:** Ravi Aggarwal, Viknesh Sounderajah, Guy Martin, Daniel S. W. Ting, Alan Karthikesalingam, Dominic King, Hutan Ashrafian, Ara Darzi

**Affiliations:** 1 Institute of Global Health Innovation, Imperial College London, London, UK; 2 Singapore Eye Research Institute, Singapore National Eye Center, Singapore, Singapore

**Keywords:** Whole body imaging, Translational research

## Abstract

Deep learning (DL) has the potential to transform medical diagnostics. However, the diagnostic accuracy of DL is uncertain. Our aim was to evaluate the diagnostic accuracy of DL algorithms to identify pathology in medical imaging. Searches were conducted in Medline and EMBASE up to January 2020. We identified 11,921 studies, of which 503 were included in the systematic review. Eighty-two studies in ophthalmology, 82 in breast disease and 115 in respiratory disease were included for meta-analysis. Two hundred twenty-four studies in other specialities were included for qualitative review. Peer-reviewed studies that reported on the diagnostic accuracy of DL algorithms to identify pathology using medical imaging were included. Primary outcomes were measures of diagnostic accuracy, study design and reporting standards in the literature. Estimates were pooled using random-effects meta-analysis. In ophthalmology, AUC’s ranged between 0.933 and 1 for diagnosing diabetic retinopathy, age-related macular degeneration and glaucoma on retinal fundus photographs and optical coherence tomography. In respiratory imaging, AUC’s ranged between 0.864 and 0.937 for diagnosing lung nodules or lung cancer on chest X-ray or CT scan. For breast imaging, AUC’s ranged between 0.868 and 0.909 for diagnosing breast cancer on mammogram, ultrasound, MRI and digital breast tomosynthesis. Heterogeneity was high between studies and extensive variation in methodology, terminology and outcome measures was noted. This can lead to an overestimation of the diagnostic accuracy of DL algorithms on medical imaging. There is an immediate need for the development of artificial intelligence-specific EQUATOR guidelines, particularly STARD, in order to provide guidance around key issues in this field.

## Introduction

Artificial Intelligence (AI), and its subfield of deep learning (DL)^1^, offers the prospect of descriptive, predictive and prescriptive analysis, in order to attain insight that would otherwise be untenable through manual analyses^2^. DL-based algorithms, using architectures such as convolutional neural networks (CNNs), are distinct from traditional machine learning approaches. They are distinguished by their ability to learn complex representations in order to improve pattern recognition from raw data, rather than requiring human engineering and domain expertise to structure data and design feature extractors^3^.

Of all avenues through which DL may be applied to healthcare; medical imaging, part of the wider remit of diagnostics, is seen as the largest and most promising field^4,5^. Currently, radiological investigations, regardless of modality, require interpretation by a human radiologist in order to attain a diagnosis in a timely fashion. With increasing demands upon existing radiologists (especially in low-to-middle-income countries)^6–8^, there is a growing need for diagnosis automation. This is an issue that DL is able to address^9^.

Successful integration of DL technology into routine clinical practice relies upon achieving diagnostic accuracy that is non-inferior to healthcare professionals. In addition, it must provide other benefits, such as speed, efficiency, cost, bolstering accessibility and the maintenance of ethical conduct.

Although regulatory approval has already been granted by the Food and Drug Administration for select DL-powered diagnostic software to be used in clinical practice^10,11^, many note that the critical appraisal and independent evaluation of these technologies are still in their infancy^12^. Even within seminal studies in the field, there remains wide variation in design, methodology and reporting that limits the generalisability and applicability of their findings^13^. Moreover, it is noted that there has been no overarching medical specialty-specific meta-analysis assessing diagnostic accuracy of DL performance, particularly in ophthalmology, respiratory medicine and breast surgery, which have the most diagnostic studies to date^13^.

Therefore, the aim of this review is to (1) quantify the diagnostic accuracy of DL in speciality-specific radiological imaging modalities to identify or classify disease, and (2) to appraise the variation in methodology and reporting of DL-based radiological diagnosis, in order to highlight the most common flaws that are pervasive across the field.

## Results

### Search and study selection

**Fig. 1:**
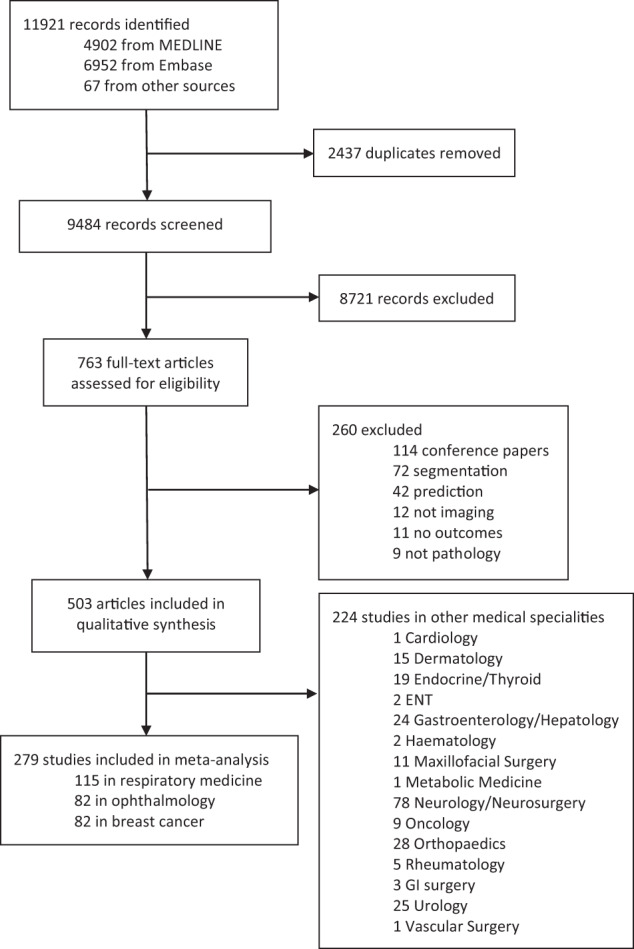
PRISMA flow diagram of included studies. PRISMA (preferred reporting items for systematic reviews and meta-analyses) flow diagram of included studies.

Our search identified 11,921 abstracts, of which 9484 were screened after duplicates were removed. Of these, 8721 did not fulfil inclusion criteria based on title and abstract. Seven hundred sixty-three full manuscripts were individually assessed and 260 were excluded at this step. Five hundred three papers fulfilled inclusion criteria for the systematic review and contained data required for sensitivity, specificity or AUC. Two hundred seventy-three studies were included for meta-analysis, 82 in ophthalmology, 115 in respiratory medicine and 82 in breast cancer (see Fig. 1). These three fields were chosen to meta-analyse as they had the largest numbers of studies with available data. Two hundred twenty-four other studies were included for qualitative synthesis in other medical specialities. Summary estimates of imaging and speciality-specific diagnostic accuracy metrics are described in Table 1. Units of analysis for each speciality and modality are indicated in Tables 2–4.

**Table 1:** Summary estimates of pooled speciality and imaging modality specific diagnostic accuracy metrics.

Imaging modality	Diagnosis	AUC	95% CI	*I*^2^	Sensitivity	95% CI	*I*^2^	Specificity	95% CI	*I*^2^	PPV	95% CI	*I*^2^	NPV	95% CI	*I*^2^	Accuracy	95% CI	*I*^2^	*F*1 score	95% CI	*I*^2^
*Ophthalmology imaging*																						
RFP	DR	0.939	0.920–0.958	99.9	0.976	0.975–0.977	99.9	0.902	0.889–0.916	99.7	0.389	0.166–0.612	99.7	1	1	90.6	0.927	0.899–0.955	96.3			
RFP	AMD	0.963	0.948–0.979	99.3	0.973	0.971–0.974	99.9	0.924	0.896–0.952	99.6							0.797	0.719–0.875	99.9			
RFP	Glaucoma	0.933	0.924–0.942	99.6	0.883	0.862–0.904	99.9	0.918	0.898–0.938	99.7							0.881	0.847–0.915	97.7			
RFP	ROP				0.96	0.913–1.008	99.5	0.907	0.749–1.066	99.8												
OCT	DR	1	0.999–1.0	98.1	0.954	0.937–0.972	98.9	0.993	0.991–0.994	98.2							0.97	0.959–0.981	97.5			
OCT	AMD	0.969	0.955–0.983	99.4	0.997	0.996–0.997	99.7	0.932	0.914–0.950	98.9							0.936	0.906–0.965	99.6			
OCT	Glaucoma	0.964	0.941–0.986	77.7																		
*Respiratory imaging*																						
CT	Lung nodules	0.937	0.924–0.949	97	0.86	0.831–0.890	99.7	0.896	0.871–0.921	99.2	0.785	0.711–0.858	99.2				0.889	0.870–0.908	98.4	0.79	0.747–0.834	97.9
CT	Lung cancer	0.887	0.847–0.928	95.9	0.837	0.780–0.894	94.6	0.826	0.735–0.918	98.1							0.827	0.784–0.870	81.7			
X-ray	Nodules	0.884	0.842–0.925	99.6	0.75	0.634–0.866	99	0.944	0.912–0.976	98.4	0.86	0.736–0.984	99.8							0.894	0.842–0.945	81.4
X-ray	Mass	0.864	0.827–0.901	99.7	0.801	0.683–0.919	99.7															
X-ray	Abnormal	0.917	0.869–0.966	99.9	0.873	0.762–0.985	99.9	0.894	0.860–0.929	98.7	0.85	0.567–1.133	100				0.859	0.736–0.983	99	0.76	0.558–0.962	99.7
X-ray	Atelectasis	0.824	0.783–0.866	99.7																		
X-ray	Cardiomegaly	0.905	0.871–0.938	99.7																		
X-ray	Consolidation	0.875	0.800–0.949	99.9	0.914	0.816–1.013	99.5	0.751	0.637–0.866	98.6							0.897	0.828–0.966	96.4			
X-ray	Pulmonary oedema	0.893	0.843–0.944	99.9																		
X-ray	Effusion	0.906	0.862–0.950	99.8																		
X-ray	Emphysema	0.885	0.855–0.916	99.7																		
X-ray	Fibrosis	0.834	0.796–0.872	99.7																		
X-ray	Hiatus hernia	0.894	0.858–0.930	99.8																		
X-ray	Infiltration	0.724	0.682–0.767	99.6																		
X-ray	Pleural thickening	0.816	0.762–0.870	99.8																		
X-ray	Pneumonia	0.845	0.782–0.907	99.9	0.951	0.936–0.965	96.3	0.716	0.480–0.953	100	0.681	0.367–0.995	100				0.763	0.559–0.968	100	0.889	0.838–0.941	97.6
X-ray	Pneumothorax	0.91	0.863–0.957	99.9	0.718	0.433–1.004	100	0.918	0.870–0.965	99.9	0.496	0.369–0.623	100									
X-ray	Tuberculosis	0.979	0.978–0.981	99.6	0.998	0.997–0.999	99.6	1	0.999–1.000	95.3							0.94	0.921–0.959	84.6			
*Breast imaging*																						
MMG	Breast cancer	0.873	0.853–0.894	98.8	0.851	0.779–0.923	99.9	0.882	0.859–0.905	97.2							0.905	0.880–0.930	97.9			
Ultrasound	Breast cancer	0.909	0.881–0.936	91.7	0.853	0.815–0.891	93.9	0.901	0.870–0.931	96.6	0.804	0.727–0.880	93.7	0.922	0.851–0.992	97.2	0.873	0.841–0.906	87.5	0.855	0.803–0.906	87.9
MRI	Breast cancer	0.868	0.850–0.886	27.8	0.786	0.710–0.861	80.5	0.788	0.697–0.880	86.2												
DBT	Breast cancer	0.908	0.880–0.937	63.2	0.831	0.675–0.988	97.6										0.918	0.905–0.930	0

**Table 2:** Characteristics of ophthalmic imaging studies.

Study	Model	Prospective?	Test set	Population	Test datasets	Type of internal validation	External validation	Reference standard	AI vs clinician?	Imaging modality	Pathology
Abramoff et al. 2016	AlexNet/VGG	No	1748	Photographs	Messidor-2	NR	No	Expert consensus	No	Retinal fundus photography	Referable DR
Abramoff et al. 2018^14^	AlexNet/VGG	Yes	819	Patients	Prospective cohort from 10 primary care practice sites in USA	NR	Yes	Expert consensus	No	Retinal fundus photography	More than mild DR
Ahn et al. 2018	(a) Inception-v3; (b) customised CNN	No	(a) 464; (b) 464	Images	Kim’s Eye Hospital, Korea	Random split	No	Expert consensus	No	Retinal fundus photography	Early and advanced glaucoma
Ahn et al. 2019	ResNet50	No	219	Photographs	Kim’s Eye Hospital, Korea	Random split	No	Expert consensus	No	Retinal fundus photographs	Pseudopapilloedema
Al-Aswad et al. 2019^46^	Pegasus (ResNet50)	No	110	Photographs	Singapore Malay Eye Study	Random split	No	Existing diagnosis from source data	Yes	Retinal fundus photographs	Glaucoma
Alqudah et al. 2019^22^	AOCT-NET	No	1250	Scans	Farsiu Ophthalmology 2013	Hold-out method	Yes	NR	No	OCT	(a) AMD; (b) DME
Arcadu et al. 2019	Inception-v3	No	(a) 1237; (b) 1798	Images	RISE/RIDE trials	Random split	No	Expert consensus	No	Retinal fundus photography	(a) DME—central subfield thickness >400 µm; (b) DME—central fovea thickness >400 µm
Asaoka et al. 2016	Deep feed-forward neural network with stacked denoising autoencoder	No	279	Eyes	University of Tokyo Hospital, Tokyo	Random split	No	Other imaging technique	No	Visual Fields	Preperimetric open-angle glaucoma
Asaoka et al. 2019	Customised CNN	No	196	Images	University of Tokyo Hospital, Tokyo	Random split	No	Expert consensus	No	OCT	Early open-angle glaucoma
Asaoka et al. 2019^23^	ResNet50	No	(a) 205; (b) 171	Scans	(a) Iinan Hospital; (b) Hiroshiuma University	NR	Yes	Expert consensus	No	OCT	Glaucoma
Bellemo et al. 2019^15^	VGG/ResNet	Yes	3093	Eyes	Kitwe Central Hospital Eye Unit, Zambia	NA	Yes	Expert consensus	No	Retinal fundus photography	(a) Referable DR; (b) vision-threatening DR; (c) DME
Bhatia et al. 2019^24^	VGG-16	No	(a) 4686; (b) 384; (c) 148; (d) 100; (e) 135; (f) 135; (g) 148; (h) 100	Scans	(a) Shiley Eye Institute of the UCDS; (b) Devers Eye Institute; (c) Noor Eye Hospital; (d) Ophthalmica Ophthalmology Greece; (e) Cardiff University; (f) Cardiff University; (g) Noor Eye Hospital; (h) Ophthalmica Ophthalmology Greece	NA	Yes	(a) Expert consensus; (b) NR; (c) NR; (d) NR; (e) Expert consensus + further imaging; (f) expert consensus + further imaging; (g) NR; (h) NR	No	OCT	(a) Abnormal scan; (b–f) AMD; (g–h) DME
Brown et al. 2018^47^	Inception-v1 and U-Net	No	100	Photographs	i-ROP	Hold-out method	No	Expert consensus	Yes	Retinal fundus photography	Plus disease in ROP
Burlina et al. 2017^49^	DCNN	No	5664	Images	AREDS 4 dataset	NR	No	Expert consensus	Yes	Retinal fundus photography	AMD-AREDS 4 step
Burlina et al. 2018^48^	ResNet50	No	5000	Images	AREDS	Random split	No	Reading centre grader	No	Retinal fundus photographs	Referable AMD
Burlina et al. 2018^50^	AlexNet	No	13,480	Photographs	NIH AREDS	NR	No	Reading centre grader	Yes	Retinal fundus photography	Referable AMD
Burlina et al. 2018	ResNet50	No	(a) 6654; (b) 58,978	Images	(a) AREDS 9 dataset; (b) AREDS 4 dataset	NR	No	Reading centre grader	Yes	Retinal fundus photography	(a) AMD-AREDS 4 step; (b) AMD-AREDS 9 step
Chan et al. 2018^25^	AlexNet, VGGNet, GoogleNet	No	4096	Images	SERI	NR	Yes	Reading centre grader	No	OCT	DME
Choi et al. 2017	VGG-19	No	(a) 3000; (b) 3000	Photographs	STARE database	Random split	No	Expert consensus	No	Retinal fundus photographs	(a) DR; (b) AMD
Christopher et al. 2018^16^	(a) VGG-16; (b) Inception-v3; (c) ResNet50	Yes	1482	Images	ADAGES and DIGS	Random split	No	Expert consensus	No	Retinal fundus photography	Glaucomatous optic neuropathy
Das et al. 2019	VGG-16	No	1000	Images	UCSD	Hold-out method	No	Expert consensus	No	OCT	DME
De Fauw et al. 2018^51^	(a) U-Net (b) customised CNN	No	(a) 997; (b) 116	(a) Scans (Topcon device); (b) scans (Spectralis device)	Moorfields, London	Random split	No	Follow up	Yes	OCT	Urgent referral eye disease
ElTanboly et al. 2016	Deep fusion classification network (DFCN)	No	12	OCT scans		Hold-out method	No	NR	No	OCT	Early DR
Gargeya et al. 2017^26^	CNN	No	(a) 15,000 (b) 1748; (c) 463	Photographs	(a) EyePACS-1; (b) Messidor-2; (c) E-Opthma	Random split	Yes	Expert consensus	No	Retinal fundus photography	DR
Gomez-Valverde et al. 2019^52^	VGG-19	No	494	Photographs	ESPERANZA	Random split	No	Expert consensus	Yes	Retinal fundus photographs	Glaucoma suspect or glaucoma
Grassman et al. 2018^27^	Ensemble:random forest	No	(a) 12,019; (b) 5555	Images	(a) AREDS dataset; (b) KORA dataset	Random split	Yes	Reading centre grader	No	Retinal fundus photography	AMD-AREDS 9 step
Gulshan et al. 2019^17^	Inception-v3	Yes	3049	Photographs	Prospective	NA	Yes	Expert consensus	Yes	Retinal fundus photographs	Referable DR
Gulshan et al. 2016^28^	Inception-v3	No	(a) 8788; (b) 1745	Photographs	(a) EyePACS-1; (b) Messidor-2	Random split	Yes	Reading centre grader	Yes	Retinal fundus photography	Referable DR
Hwang et al. 2019^29^	(a) ResNet50; (b) VGG-16; (c) Inception-v3; (d) ResNet50; (e) VGG-16; (f) Inception-v3	No	(a–c) 3872; (d–f) 750	Images	(a–c) Department of Ophthalmology of Taipei Veterans General Hospital; (d–f) External validation	Random split	Yes	Expert consensus	Yes	OCT	AMD-AREDS 4 step
Jammal et al. 2019^53^	ResNet34	No	490	Images		Randomly drawn from test sample	No	Reading centre grader	Yes	Retinal fundus photographs	Glaucomatous optic neuropathy
Kanagasingham et al. 2018^21^	DCNN	Yes	398	Patients	Primary Care Practice, Midland, Western Australia	NA	Yes	Reading centre grader	No	Retinal fundus photography	Referable DR
Karri et al. 2017	GoogLeNet	No	21	Scans	Duke University	Random split	No	NR	No	OCT	(a) DME; (b) dry AMD
Keel et al. 2018^18^	Inception-v3	Yes	93	Images	St Vincent’s Hospital Melbourne and University Hospital Geelong, Barwon Health	NA	Yes	Reading centre grader	No	Retinal fundus photography	Referable DR
Keel et al. 2019^30^	CNN	No	86,202	Photographs	Melbourne Collaborative Cohort Study	Hold-out method	Yes	Expert consensus	No	Retinal fundus photographs	Neovascular AMD
Kermany et al. 2018^54^	Inception-v3	No	(a) 1000; (b–d) 500	Scans	Shiley Eye Institute of the University of California San Diego, the California Retinal Research Foundation, Medical Centre Ophthalmology Associates, the Shanghai First People’s Hospital, and Beijing Tongren Eye Centre	Random split	No	Consensus involving experts and non-experts	Yes	OCT	(a) Choroidal neovascularisation vs DME vs drusen vs normal; (b) choroidal neovascularisation; (c) DME; (d) AMD
Krause et al. 2018^31^	CNN	No	1958	Images	EyePACS-2	Hold-out method	Yes	Expert consensus	No	Retinal fundus photographs	Referable DR
Lee et al. 2017	VGG-16	No	2151	Scans		Random split	No	Routine clinical notes	No	OCT	AMD
Lee et al. 2019	CNN	No	200	Photographs	Seoul National University Hospital	Hold-out method	No	Other imaging technique	No	Retinal fundus photographs	Glaucoma
Li et al. 2018^108^	Inception-v3	No	8000	Scans	Guangdong (China)	Random split	No	Expert graders	No	Retinal fundus photography	Glaucomatous optic neuropathy
Li et al. 2019^55^	VGG-16	No	1000	Images	Shiley Eye Institute of the University of California San Diego, the California Retinal Research Foundation, Medical Centre Ophthalmology Associates, the Shanghai First People’s Hospital, and Beijing Tongren Eye Centre	Random split	No	Expert consensus	No	OCT	Choroidal neovascularisation vs DME vs drusen Vs normal
Li et al. 2019	OCT-NET	No	859	Scans	Wenzhou Medical University	Random split	No	Expert graders	No	OCT	Early DR
Li et al. 2019^33^	Inception-v3	No	800	Images	Messidor-2	Random split	Yes	Reading centre grader	No	Retinal fundus photographs	Referable DR
Li et al. 2019	ResNet50	No	1635	Images	Shanghai Zhongshan Hospital and the Shanghai First People’s Hospital	Random split	No	Reading centre grader	Yes	OCT	DME
Lin et al. 2019^109^	CC-Cruiser	Yes—multicentre RCT	350	Images	Multicentre RCT	NA	NA	Expert consensus	Yes	Slit-lamp photography	Childhood cataracts
Li F et al. 2018	VGG-15	No	300	Images	NR	Random split	No	NR	No	Visual Fields	Glaucoma
Li Z et al. 2018^33^	CNN	No	35,201	Photographs	NIEHS, SiMES, AusDiab	Random split	Yes	Reading centre grader	No	Retinal fundus photographs	Referable DR
Liu et al. 2018^35^	ResNet50	No	(a) 754; (b) 30	Photographs	(a) NR; (b) HRF	Random split	Yes	Reading centre grader	Yes	Retinal fundus photographs	Glaucomatous optic discs
Liu et al. 2019^34^	CNN	No	(a) 28,569; (b) 20,466; (c) 12,718; (d) 9305; (e) 29,676; (f) 7877	Photographs	(a) Local Validation (Chinese Glaucoma Study Alliance); (b) Beijing Tongren Hospital; (c) Peking University Third Hospital; (d) Harbin Medical University First Hospital; (e) Handan Eye Study; (f) Hamilton Glaucoma Centre	Random split	Yes	Consensus involving experts and non-experts	No	Retinal fundus photographs	Glaucomatous optic neuropathy
Long et al. 2017^56^	DCNN	No	57	Images	Multihospital clinical trial	Hold-out method	No	Expert consensus	Yes	Ocular images	Congenital Cataracts
MacCormick et al. 2019^36^	DenseNet	No	(a) 130; (b) 159	Images	(a) ORIGA; (b) RIM-ONE	Random split	Yes	(a) NR; (b) expert consensus	No	Retinal fundus photography	Glaucomatous optic discs
Maetshke et al. 2019	3D CNN	No	110	OCT scans	Fivefold cross validation	Random split	No	Follow up	No	OCT	Glaucomatous optic neuropathy
Matsuba et al. 2018^57^	DCNN	No	111	Images	Tsukazaki Hospital	NR	No	Expert consensus + further imaging	Yes	Retinal fundus photography (optos)	Exudative AMD
Medeiros et al. 2019	ResNet34	No	6292	Images	Duke University	Random split	No	Follow up	No	Retinal fundus photography	Glaucomatous optic neuropathy
Motozawa et al. 2019	CNN	No	382	Images	Kobe City Medical Centre	Random split	No	Routine clinical notes	No	OCT	AMD
Muhammad et al. 2017	AlexNet	No	102	Images	NR	NR	No	Expert consensus	No	OCT	Glaucoma suspect or glaucoma
Nagasato et al. 2019	VGG-16	No	466	Images	NR	K-fold cross validation	No	NR	No	Retinal fundus photography (optos)	Retinal vein occlusion
Nagasato et al. 2019^58^	DNN	No	322	Scans	Tsukazaki Hospital and Tokushima University Hospital	K-fold cross validation	No	Expert graders	Yes	OCT	Retinal vein occlusion
Nagasawa et al. 2019	VGG-16	No	378	Images	Tsukazaki Hospital and Tokushima University Hospital	K-fold cross validation	No	Expert graders	No	Retinal fundus photography (optos)	Proliferative diabetic retinopathy
Ohsugi et al. 2017	DCNN	No	166	Images	Tsukazaki Hospital	Random split	No	Expert consensus	No	Retinal fundus photography (optos)	Rhegmatogenous retinal detachment
Peng et al. 2019^59^	Inception-v3	No	900	Images	AREDS	Random split	No	Reading centre grader	Yes	Retinal fundus photography	Age-related macular degeneration-AREDS 4 step
Perdomo et al. 2019	OCT-NET	No	2816	Images	SERI-CUHK data set	Random split	No	Expert graders	No	OCT	DME
Phan et al. 2019	DenseNet201	No	828	Images	Yamanashi Koseiren Hospital		No	Expert consensus + further imaging	No	Retinal fundus photography	Glaucoma
Phene et al. 2019^37^	Inception-v3	No	(a) 1205; (b) 9642; (c) 346	Images	(a) EyePACS, Inoveon, the United Kingdom Biobank, the Age-Related Eye Disease Study, and Sankara Nethralaya; (b) Atlanta Veterans Affairs (VA) Eye Clinic; (c) Dr. Shroff’s Charity Eye Hospital, New Delhi, India	Random split	Yes	Reading centre grader	Yes	Retinal fundus photographs	Glaucomatous optic neuropathy
Prahs et al. 2017	GoogLeNet	No	5358	Images	Heidelberg Eye Explorer, Heidelberg Engineering	Random split	No	Expert graders	No	OCT	Injection vs No injection for AMD
Raju et al. 2017	CNN	No	53,126	Images	EyePACS-1	Random split	No	NR	No	Retinal fundus photography	Referable DR
Ramachandran et al. 2018^38^	Visiona intelligent diabetic retinopathy screening platform	No	(a) 485; (b) 1200	Photographs	(a) ODEMS; (b) Messidor	NA	Yes	Expert graders	No	Retinal fundus photographs	Referable DR
Raumviboonsuk et al. 2019^39^	Inception-v4	No	(a–c) 25,348; (d) 24,332	Images	National screening program for DR in Thailand	NA	Yes	Expert consensus	Yes	Retinal fundus photography	(a) Moderate non-proliferative DR or worse; (b) severe non-proliferative DR or worse; (c) proliferative DR; (d) referable DME
Redd et al. 2018	Inception-v1 and U-Net	No	4861	Images	Multicentre i-ROP study	NR	No	Expert graders + further imaging	No	Retinal fundus photography	Plus disease in ROP
Rogers et al. 2019^45^	Pegasus (ResNet50)	No	94	Photographs	EODAT	NA	Yes	Reading centre grader	Yes	Retinal fundus photographs	Glaucomatous optic neuropathy
Sandhu et al. 2018^19^	Deep fusion SNCAE	Yes	160	Scans	University of Waikato	NA	No	Clinical diagnosis	No	Retinal fundus photographs	Non-proliferative DR
Sayres et al. 2019^40^	Inception-v4	No	2000	Images	EyePACS-2	NA	Yes	Expert consensus	Yes	Retinal fundus photographs	Referable DR
Shibata et al. 2018^60^	(a) ResNet; (b) VGG-16	No	110	Images	Matsue Red Cross Hospital	Random split	No	Expert consensus	Yes	Retinal fundus photography	Glaucoma
Stevenson et al. 2019	Inception-v3	No	(a) 2333; (b) 2283; (c) 2105	Photographs	Publicly available databases	Random split	No	Existing diagnosis from source data	No	Retinal fundus photographs	(a) Glaucoma; (b) DR; (c) AMD
Ting et al. 2017^41^	VGGNet	No	(a) 71,896; (b) 15,798; (c) 3052; (d) 4512; (e) 1936; (f) 1052; (g) 1968; (h) 2302; (i) 1172; (j) 1254; (k) 7706; (l) 35,948; (m) 35,948	Images	(a) Singapore National Diabetic Retinopathy Screening Program 2014–2015; (b) Guangdong (China); (c) Singapore Malay Eye Study; (d) Singapore Indian Eye Study; (e) Singapore Chinese Eye Study; (f) Beijing Eye Study; (g) African American Eye Disease Study; (h) Royal Victoria Eye and Ear Hospital; (i) Mexican; (j) Chinese University of Hong Kong, (k, l) Singapore National Diabetic Retinopathy Screening Program 2014–2015	Random split	Yes	Expert consensus	No	Retinal fundus photography	Referable DR
Ting et al. 2019^42^	VGGNet	No	85,902	Images	Combined eight datasets	NA	Yes	Consensus involving experts and non-experts	No	Retinal fundus photography	(a) Any DR; (b) referable DR; (c) vision-threatening DR
Treder et al. 2017	Inception-v3	No	100	Scans	NR	Hold-out method	No	NR	No	OCT	Exudative AMD
van Grinsven et al. 2016^44^	(a) Ses CNN 60; (b) NSesCNN170	No	1200	Images	Messidor	Random split	Yes	Existing diagnosis from source data	Yes	Retinal fundus photographs	Retinal haemorrhage
Verbraak et al. 2019^43^	AlexNet/VGG	No	1293	Images	Netherlands Star-SHL	NA	Yes	Expert consensus	No	Retinal fundus photography	(a) DR-vision-threatening; (b) DR- more than mild
Xu et al. 2017	CNN	No	200	Photographs	Kaggle	Random split	No	Existing diagnosis from source data	No	Retinal fundus photographs	DR
Yang et al. 2019	VGGNet	No	500	Photographs	Intelligent Ophthalmology Database of Zhejiang Society for Mathematical Medicine in China	Hold-out method	No	Expert consensus	No	Retinal fundus photographs	Referable DR
Yoo et al. 2019	VGG-19	No	900	Scans	Project Macula	Random split	No	NR	No	(a) OCT; (b) retinal fundus photographs	AMD
Zhang et al. 2019^61^	VGG-16	No	1742	Images	Telemed-R screening	Random split	No	Expert consensus	Yes	Retinal fundus photographs	ROP
Zheng et al. 2019^20^	Inception-v3	Yes	102	Scans	Joint Shantou International Eye Centre of Shantou University and the Chinese University of Hong Kong (JSIEC)	Hold-out method	No	NR	No	OCT	Glaucomatous optic neuropathy

**Table 3:** Characteristics of respiratory imaging studies.

Study	Model	Prospective?	Test set	Population	Test datasets	Type of internal validation	External validation	Reference standard	AI vs clinician	Imaging modality	Body system/disease
Abiyev et al. 2018	CNN	No	380	Images	Chest X-ray14	Random split	No	Routine clinical reports	No	X-ray	Abnormal X-ray
Al-Shabi et al. 2019	Local-Global	No	848	Nodules	LIDC-IDRI	NR	No	Expert readers	No	CT	Nodules
Alakwaa et al. 2017	U-Net	No	419	Scans	Kaggle Data Science Bowl	Random split	No	Expert reader, existing labels in dataset	No	CT	Lung cancer
Ali et al. 2018	3D CNN	No	668	Nodules	LIDC-IDRI	Random split	No	Expert readers	No	CT	Nodules
Annarumma et al. 2019^110^	CNN	No	15,887	Images	Kings College London	Hold-out method	No	Routine clinical reports	No	X-ray	(a) Critical radiographs; (b) normal radiographs
Ardila et al. 2019^64^	Inception-v1	No	(a) 6716; (b) 1139	Scans	(a) National Lung Cancer Screening Trial; (b) Northwestern Medicine	Random split	Yes	Histopathology, follow up	Yes	CT	Lung cancer
Baltruschat et al. 2019	ResNet50	No	22,424	X-rays	Chest X-ray14	Random split	No	Routine clinical reports	No	X-ray	(a) Abnormal chest X-ray; (b) normal chest X-ray; (c) atelectasis; (d) cardiomegaly; (e) effusion; (f) infiltration; (g) mass; (h) nodule (i) pneumonia; (j) pneumothorax; (k) consolidation; (l) oedema; (m) emphysema; (n) fibrosis; (o) pleural thickening; (p) hernia
Bar et al. 2018	CNN	No	194	Images	Diagnostic Imaging Department of Sheba Medical Centre, Tel Hashomer, Israel	Random split	No	Expert readers	No	X-ray	(a) Abnormal X-ray; (b) cardiomegaly
Becker et al. 2018^62^	CNN	Yes	21	X-rays	Infectious Diseases Institute in Kampala, Uganda	Random split	No	Expert consensus	No	X-ray	Tuberculosis
Behzadi-Khormouji et al. 2020	(a) ChestNet; (b) VGG-16; (c) DenseNet121	No	582	X-rays	Guangzhou Women and Children’s Medical Centre	NR	No	Expert readers	No	X-ray	Consolidation
Beig et al. 2019	CNN	No	145	Scans	Erlangen Germany, Waukesha Wis, Cleveland Ohio, Tochigi-ken Japan	Random split	No	Histopathology	No	CT	Lung cancer
Causey et al. 2018	CNN	No	(a) 424; (b) 213	Nodules	LIDC-IDRI	Random split	No	Expert readers	No	CT	Nodules
Cha et al. 2019^76^	ResNet50	No	(a) 1483; (b) 500	X-rays	Samsung Medical Centre, Seoul	Random split	No	Other imaging, expert readers	Yes	X-ray	(a) Lung cancer; (b) T1 lung cancer
Chae et al. 2019^77^	Ct-LUNGNET	No	60	Nodules	Chonbuk National University Hospital	Random split	No	Expert readers, histopathology, follow up	Yes	CT	Nodules
Chakravarthy et al. 2019	Probabilistic neural network	No	119	Scans	LIDC/IDRI	NR	No	NR	No	CT	Lung cancer
Chen et al. 2019	3D CNN	No	3674	Nodules	LIDC-IDRI	NR	No	Expert readers	No	CT	Nodules
Cheng et al. 2016	Stacked denoising autoencoder	No	1400	Nodules	LIDC-IDRI	Random split	No	Expert readers	No	CT	Nodules
Cicero et al. 2017	GoogLeNet	No	2443	Images	Department of Medical Imaging, St Michael’s Hospital, Toronto	Random split	No	Expert readers, routine clinical reports	No	X-ray	(a) Effusion; (b) oedema; (c) consolidation; (d) cardiomegaly; (e) pneumothorax
Ciompi et al. 2017^78^	ConvNet	No	639	Nodules	Danish Lung Cancer Screening Trial (DLCST)	Random split	No	Non-expert readers	Yes	CT	(a) Nodules—solid; (b) nodules—calcified; (c) nodules—part-solid; (d) nodules—non-solid; (e) nodules—perifissural; (f) nodules—spiculated
Correa et al. 2018	CNN	No	60	Images	Lima, Peru	NR	No	Expert readers	No	Ultrasound	Paediatric pneumonia
da Silva et al. 2017	Evolutionary CNN	No	200	Nodules	LIDC-IDRI	Hold-out method	No	Expert readers	No	CT	Nodules
da Silva et al. 2018	Particle swarm optimisation algorithm within CNN	No	2000	Nodules	LIDC-IDRI	Random split	No	Expert readers	No	CT	Nodules
Dai et al. 2018	3D DenseNet-40	No	211	Nodules	LIDC-IDRI	Random split	No	Expert readers	No	CT	Nodules
Dou et al. 2017	3D CNN	No	1186	Nodules	LUNA16	NR	No	Expert readers	No	CT	Nodules
Dunnmon et al. 2019^79^	ResNet18	No	533	Images	Stanford University	Hold-out method	No	Expert consensus	Yes	X-ray	Abnormal X-ray
Gao et al. 2018	CNN	No	20	Scans	University Hospitals of Geneva	Random split	No	NR	No	CT	Interstitial lung disease
Gong et al. 2019	3D SE-ResNet	No	1186	Nodules	LUNA16	NR	No	Expert readers	No	CT	Nodules
Gonzalez et al. 2018	CNN	No	1000	Scans	ECLIPSE study	Random split	No	NR	No	CT	COPD
Gruetzemacher et al. 2018	DNN	No	1186	Nodules	LUNA16	Ninefold cross validation	No	NR	No	CT	Nodules
Gu et al. 2018	3D CNN	No	1186	Nodules	LUNA16	Tenfold cross validation	No	Expert readers	No	CT	Nodules
Hamidian et al. 2017	3D CNN	No	104	Nodules	LIDC-IDRI	Random split	No	Expert readers	No	CT	Nodules
Han et al. 2018	Multi-CNNs	No	812	Regions of interest	LIDC-IDRI	Random split	No	NR	No	CT	Ground glass opacity
Heo et al. 2019	VGG-19	No	37,677	X-rays	Yonsei University Hospital, South Korea	Hold-out method	No	Expert readers	No	X-ray	Tuberculosis
Hua et al. 2015	(a) CNN; (b) deep belief network	No	2545	Nodules	LIDC-IDRI	NR	No	Expert readers	No	CT	Nodules
Huang et al. 2019	R-CNN	No	176	Scans	LIDC-IDRI	Random split	No	Expert readers	No	CT	Nodules
Huang et al. 2019	Amalgamated-CNN	No	1795	Nodules	LIDC/IDRI and Ali Tianchi medical	Random split	No	Expert readers	No	CT	Nodules
Hussein et al. 2019	VGG	No	1144	Nodules	LIDC/IDRI	Random split	No	Expert readers	No	CT	Lung cancer
Hwang et al. 2018^67^	DCNN	No	(a) 450; (b) 183; (c) 140; (d) 173; (e) 170; (f) 132; (g) 646	X-rays	(a) Internal validation; (b) Seoul National University Hospital; (c) Boromae Hospital; (d) Kyunghee University Hospital; (e) Daejeon Eulji Medical Centre; (f) Montgomery; (g)	Random split	Yes	Expert readers	Yes	X-ray	Tuberculosis
Hwang et al. 2019^65^	Lunit INSIGHT	No	1135	X-rays	Seoul National University Hospital	NA	Yes	Expert consensus, other imaging	Yes	X-ray	Abnormal chest X-ray
Hwang et al. 2019^66^	DCNN	No	(a) 1089; (b) 1015	X-rays	(a) Internal validation; (b) external validation	Random split	Yes	Expert reader, other imaging, histopathology	Yes	X-ray	Neoplasm/TB/pneumonia/pneumothorax
Jiang et al. 2018	CNN	No	25,723	Nodules	LIDC-IDRI	NR	No	Expert readers	No	CT	Nodules
Jin et al. 2018	ResNet 3D	No	1186	Nodules	LUNA16	NR	No	Expert readers	No	CT	Nodules
Jung et al. 2018	3D DCNN	No	1186	Nodules	LUNA16	NR	No	Expert readers	No	CT	Nodules
Kang et al. 2017	3D multi view-CNN	No	776	Nodules	LIDC-IDRI	NR	No	Expert readers	No	CT	Nodules
Kermany et al. 2018	Inception-v3	No	624	X-rays	Guangzhou Women and Children’s Medical Centre	Random split	No	Expert readers	Yes	X-ray	Pneumonia
Kim et al. 2019	MGI-CNN	No	1186	Nodules	LIDC/IDRI	NR	No	Expert readers	No	CT	Nodules
Lakhani et al. 2017^90^	(a) AlexNet; (b) GoogLeNet; (c) Ensemble (AlexNet + GoogLeNet); (d) Radiologist augmented	No	150	X-rays	Montgomery County MD, Shenzhen China, Belarus TB public Health Program, Thomas Jefferson University Hospital	Random split	No	Routine clinical reports, expert reader, histopathology	No	X-ray	Tuberculosis
Li et al. 2016	CNN	No	8937	Nodules	LIDC-IDRI	Random split	No	Expert readers	No	CT	Nodules
Li et al. 2019^81^	DL-CAD	No	812	Nodules	Shenzhen Hospital	NR	No	Expert consensus	Yes	CT	Nodules
Li et al. 2019^80^	CNN	No	200	Scans	Massachusetts General Hospital	Random split	No	Routine clinical reports	Yes	CT	Pneumothorax
Liang et al. 2020^68^	CNN	No	100	Images	Kaohsiung Veterans General Hospital, Taiwan	NA	Yes	Other imaging	No	X-ray	Nodules
Liang et al. 2019	(a) Custom CNN; (b) VGG-16; (c) DenseNet121; (d) Inception-v3; (e) Xception	No	624	X-rays	Guangzhou Women and Children’s Medical Centre	Random split	No	Expert readers	No	X-ray	Pneumonia
Liu et al. 2017	3D CNN	No	326	Nodules	National Lung Cancer Screening Trial and Early Lung Cancer Action Program	Fivefold cross validation	No	Histopathology, follow up	No	CT	Nodules
Liu et al. 2019	CDP-ResNet	No	539	Nodules	LIDC-IDRI	Random split	No	Expert readers	No	CT	Nodules
Liu H et al. 2019	Segmentation-based deep fusion network	No	112,120	X-rays	Chest X-ray14	NR	No	Routine clinical reports	No	X-ray	(a) Atelectasis; (b) cardiomegaly; (c) effusion; (d) infiltration; (e) mass; (f) nodule; (g) pneumonia; (h) pneumothorax; (i) consolidation; (j) oedema; (k) emphysema; (l) fibrosis; (m) fibrosis; (n) pleural thickening; (o) hernia
Majkowska et al. 2019^82^	CNN	No	(a–d) 1818; (e–h) 1962	X-rays	(a–d) Hospital group in India (Bangalore, Bhubaneshwar, Chennai, Hyderabad, New Delhi); (e–h) Chest X-ray14	Random split	No	Expert consensus	Yes	X-ray	(a) Pneumothorax (b) nodule; (c) opacity; (d) fracture; (e) pneumothorax; (f) nodule; (g) opacity; (h) fracture
Monkam et al. 2018	CNN	No	2600	Nodules	LIDC-IDRI	Random split	No	Expert readers	No	CT	Nodules
Nam et al. 2018^69^	CNN	No	(a) 600; (b) 181; (c) 182; (d) 181; (e) 149	Chest radiographs	(a) Internal validation; (b) Seoul National University Hospital; (c) Boromae Hospital; (d) National Cancer Centre, Korea; (e) University of California an Francisco Medical Centre	Random split	Yes	(a) Routine clinical reports, histopathology; (b–e) histopathology, follow up, other imaging	No	X-ray	Nodules
Naqi et al. 2018	Two-level stacked autoencoder + softmax	No	777	Nodules	LIDC-IDRI	NR	No	Expert readers	No	CT	Nodules
Nasrullah et al. 2019	Faster R-CNN	No	2562	Nodules	LIDC/IDRI	NR	No	Expert readers	No	CT	Nodules
Nibali et al. 2017	ResNet	No	166	Nodules	LIDC-IDRI	Random split	No	Expert readers	No	CT	Nodules
Nishio et al. 2018	VGG-16	No	123	Nodules	Kyoto University Hospital	Random split	No	NR	No	CT	Nodules
Onishi et al. 2019	AlexNet	No	60	Nodules	NR	NR	No	Histopathology, follow up	No	CT	Nodules
Onishi et al. 2019	Wasserstein generative adversarial network	No	60	Nodules	Fujita Health University Hospital	NR	No	Histopathology, follow up	No	CT	Nodules
Park et al. 2019^89^	YOLO	No	503	X-rays	Asan Medical Centre and Seoul National University Bundang Hospital	Hold-out method	No	Expert reader	No	X-ray	Pneumothorax
Park et al. 2019^83^	CNN	No	200	Images	Asan Medical Centre and Seoul National University Bundang Hospital	Hold-out method	No	Expert consensus	Yes	X-ray	(a) Nodules; (b) opacity; (c) effusion; (d) pneumothorax; (e) abnormal chest X-ray
Pasa et al. 2019	Custom CNN	No	220	X-rays	NIH Tuberculosis Chest X-ray dataset and Belarus Tuberculosis Portal dataset	Random split	No	NR	No	X-ray	Tuberculosis
Patel et al. 2019^84^	CheXMax	No	50	X-rays	Stanford University	Hold-out method	No	Expert reader, other imaging, clinical notes	Yes	X-ray	Pneumonia
Paul et al. 2018	VGG-s CNN	No	237	Nodules	National Lung Cancer Screening Trial	Hold-out method	No	Expert readers, follow up	No	CT	Nodules
Pesce et al. 2019	Convolution networks with attention feedback (CONAF)	No	7850	X-rays	Guy’s and St. Thomas’ NHS Foundation Trust	Random split	No	Routine clinical reports	No	X-ray	Lung lesions
Pezeshk et al. 2019	3D CNN	No	128	Nodules	LUNA16	Random split	No	Expert readers	No	CT	Nodules
Qin et al. 2019^70^	(a) Lunit; (b) qXR (Qure.ai); (c) CAD4TB	No	1196	X-rays	Nepal and Cameroon	NA	Yes	Expert readers	Yes	X-ray	Tuberculosis
Rajpurkar et al. 2018^85^	CNN	No	420	X-rays	ChestXray-14	Random split	No	Routine clinical reports	Yes	X-ray	(a) Atelectasis; (b) cardiomegaly; (c) consolidation; (d) oedema; (e) effusion; (f) emphysema; (g) fibrosis; (h) hernia; (i) infiltration; (j) mass; (k) nodule; (l) pleural thickening; (m) pneumonia; (n) pneumothorax
Ren et al. 2019	Manifold regularized classification deep neural network	No	98	Nodules	LIDC-IDRI	Random split	No	Expert readers	No	CT	Nodules
Sahu et al. 2019	Multi-section CNN	No	130	Nodules	LIDC-IDRI	Tenfold cross validation	No	Expert readers	No	CT	Nodules
Schwyzer et al. 2018	CNN	No	100	Patients	NR	NR	No	NR	No	FDG-PET	Lung cancer
Setio et al. 2016^71^	ConvNet	No	(a) 1186; (b) 50; (c) 898	(a) Nodules; (b) scans; (c) nodules	LIDC-IDRI	Fivefold cross validation	Yes	(a) Expert readers; (b, c) NR	No	CT	Nodules
Shaffie et al. 2018	Deep autoencoder	No	727	Nodules	LIDC-IDRI	NR	No	Expert readers	No	CT	Nodules
Shen et al. 2017	Multiscale CNN	No	1375	Nodules	LIDC-IDRI	NR	No	Expert readers	No	CT	Nodules
Sim et al. 2019^72^	ResNet50	No	800	Images	Freiberg University Hospital Freiburg, Massachusetts General Hospital Boston, Samsung Medical Centre Seoul, Severance Hospital Seoul	NA	Yes	Other imaging, histopathology	Yes	X-ray	Nodules
Singh et al. 2018^86^	Qure-AI	No	724	Chest radiographs	Chest X-ray8	Random split	No	Routine clinical reports	Yes	X-ray	(a) Lesions; (b) effusion; (c) hilar prominence; (d) cardiomegaly
Song et al. 2017	(a) CNN; (b) DNN; (c) stacked autoencoder	No	5024	Nodules	LIDC-IDRI	Random split	No	Expert readers	No	CT	Nodules
Stephen et al. 2019	CNN	No	2134	Images	Guangzhou Women and Children’s Medical Centre	Random split	No	NR	No	X-ray	Pneumonia
Sun et al. 2017	(a) CNN; (b) deep belief network; (c) stacked denoising autoencoder	No	88,948	Samples	LIDC-IDRI	Tenfold cross validation	No	Expert readers	No	CT	Nodules
Tan et al. 2019	CNN	No	280	Nodules	LIDC-IDRI	Tenfold cross validation	No	NR	No	CT	Nodules
Taylor et al. 2018^73^	(a) Inception-v3; (b) VGG-19; (c) Inception-v3; (d) VGG-19	No	(a, b) 1990; (c, d) 112,120	X-rays	(a,b) Internal validation (c,d) Chest X-ray14	Random split	Yes	Expert consensus	No	X-ray	Pneumothorax
Teramoto et al. 2016	CNN	No	104	Scans	Fujita Health University Hospital	NR	No	Expert reader	No	PET/CT	Nodules
Togacar et al. 2019	AlexNet + VGG-16 + VGG-19	No	1754	X-rays	Firat University, Turkey	Random split	No	NR	No	X-ray	Pneumonia
Togacar et al. 2020	(a) LeNet; (b) AlexNet; (c) VGG-16	No	100	Images	Cancer Imaging Archive	NR	No	Expert readers	No	CT	Lung cancer
Tran et al. 2019	LdcNet	No	1186	Nodules	LUNA16	Tenfold cross validation	No	Expert readers	No	CT	Nodules
Tu et al. 2017	CNN	No	20	Nodules	LIDC-IDRI	Tenfold cross validation	No	Expert readers	No	CT	(a) Nodules—non-solid; (b) nodules—part-solid; (c) nodules—solid
Uthoff et al. 2019^74^	CNN	No	100	Nodules	INHALE STUDY	NA	Yes	Histopathology, follow up	No	CT	Nodules
Walsh et al. 2018^87^	Inception-ResNet-v2	No	150	Scans	La Fondazione Policlinico Universitario A Gemelli IRCCS, Rome, Italy, and University of Parma, Parma, Italy	Random split	No	Expert readers	Yes	CT	Interstitial lung disease
Wang et al. 2017	AlexNet	No	230	X-rays	Japanese Society of Radiological Technology (JSRT) database	Tenfold cross validation	No	Other imaging	No	X-ray	Nodules
Wang et al. 2018^88^	3D CNN	No	200	Scans	Fudan University Shanghai Cancer Centre	Random split	No	Expert readers, histopathology	Yes	HRCT	Lung cancer
Wang et al. 2018	VGG-16	No	744	X-rays	JSRT, OpenI, SZCX and MC	Random split	No	Other imaging	No	X-ray	(a) Abnormal chest X-ray; (b) normal chest X-ray
Wang et al. 2019	ChestNet	No	442	X-rays	Zhejiang University School of Medicine (ZJU-2) and Chest X-ray14	Random split	No	Expert readers	No	X-ray	Pneumothorax
Wang et al. 2019	(a) AlexNet; (b) GoogLeNet; (c) ResNet	No	7580	Nodules	LIDC-IDRI	Random split	No	Expert readers	No	CT	Nodules
Wang et al. 2019	ResNet152	No	25,596	X-rays	Chest X-ray14	Random split	No	Routine clinical reports	No	X-ray	(a) Atelectasis; (b) cardiomegaly; (c) effusion; (d) infiltration; (e) mass; (f) nodule; (g) pneumonia; (h) pneumothorax; (i) consolidation; (j) oedema; (k) emphysema; (l) fibrosis; (m) pleural thickening; (n) hernia; (o) abnormal chest X-ray
Xie et al. 2018	LeNet-5	No	1972	Nodules	LIDC-IDRI	Random split	No	Expert readers	No	CT	Nodules
Xie et al. 2019	ResNet50	No	1945	Nodules	LIDC-IDRI	Tenfold cross validation	No	Expert readers	No	CT	Nodules
Yates et al. 2018	Inception-v3	No	5505	X-rays	Chest X-ray14 + Indiana University	Random split	No	Routine clinical reports	No	X-ray	Abnormal chest X-ray
Ye et al. 2019	(a) AlexNet; (b) GoogLeNet; (c) Res-Net150	No	(a) 321; (b) 321; (c) 593	(a) Nodules; (b) nodules; (c) regions of interest	(a, b) LIDC-IDRI; (c) private	Random split	No	Expert readers	No	CT	(a, b) Nodules; (c) ground glass opacity
Zech et al. 2018^75^	CNN	No	(a) 30,450; (b) 3807	X-rays	(a) Mount Sinai and Chest X-ray14; (b) Indiana University Network for Patient Care	Random split	Yes	Expert readers	No	X-ray	Pneumonia
Zhang et al. 2018	3D DCNN	No	1186	Nodules	LUNA16	NR	No	Expert readers	No	CT	Nodules
Zhang et al. 2019	Voxel-level-1D CNN	No	67	Nodules	Stony Brook University Hospital	Twofold cross validation	No	Histopathology	No	CT	Nodules
Zhang et al. 2019	3D deep dual path network	No	1004	Nodules	LIDC/IDRI	Tenfold cross validation	No	Expert readers	No	CT	Nodules
Zhang C et al. 2019	3D CNN	Yes	50	Images	Guangdong Lung Cancer Institute	Random split	Yes	Histopathology, follow up	Yes	CT	Nodules
Zhang et al. 2019^63^	Mask R-CNN	No	134	Slices	Shenzhen Hospital	Random split	No	Expert readers	No	CT/PET	Lung cancer
Zhang S et al. 2019	Le-Net5	No	762	Nodules	LIDC/IDRI	Random split	No	Expert readers	No	CT	Nodules
Zhang T et al. 2017	Deep Belief Network	No	1664	Nodules	LIDC-IDRI	Random split	No	Expert readers	No	CT	Nodules
Zhao X et al. 2018	Agile CNN	No	743	Nodules	LIDC-IDRI	Random split	No	Expert readers	No	CT	Nodules
Zhao X et al. 2019	(a) AlexNet; (b) GoogLeNet; (c) ResNet; (d) VifarNet	No	2028	Nodules	LIDC-IDRI	Random split	No	Expert readers	No	CT	Nodules
Zheng et al. 2019	CNN	No	1186	Nodules	LIDC-IDRI	Random split	No	Expert readers	No	CT	Nodules
Zhou et al. 2019	Inception-v3 and ResNet50	No	600	Images	Chest X-ray8	Random split	No	Routine clinical reports	No	X-ray	Cardiomegaly

**Table 4:** Characteristics of breast imaging studies.

Study	Model	Prospective?	Test Set	Population	Test datasets	Type of internal validation	External validation	Reference standard	AI vs clinician?	Imaging modality	Body system/disease
Abdelsamea et al. 2019	CNN	No	118	Images	NR	Tenfold cross validation	No	NR	No	Mammogram	Breast cancer
Agnes et al. 2020	Multiscale all CNN	No	322	Images	mini-MIAS	Random split	No	Existing labels from dataset	No	Mammogram	Breast cancer
Akselrod-Ballin et al. 2017	Faster R-CNN	No	170	Images	Multicentre hospital data set	Random split	No	Expert reader	No	Mammogram	Breast cancer
Al-Antari et al. 2018	YOLO	No	410	Images	INbreast	Random split	No	Expert reader, histology,	No	Mammogram	Breast cancer
Al-Antari et al. 2018	DBN	No	150	Images	DDSM	Random split	No	Follow up, histology, expert reader	No	Mammogram	Breast cancer
Al-Masni et al. 2018	YOLO	No	120	Images	DDSM	Random split	No	Follow up, histology, expert reader	No	Mammogram	Breast cancer
Antropova et al. 2017	VGG-19	No	(a) 690; (b) 245; (c) 1125	(a) Lesions; (b) images; (c) lesions	Private	Random split	No	Histology	No	(a) MRI; (b) mammogram; (c) ultrasound	Breast cancer
Antropova et al. 2018	VGGNet	No	138	Lesions	University of Chicago	Random split	No	Histology	No	MRI	Breast cancer
Antropova et al. 2018	VGGNet	No	141	Lesions	University of Chicago	Random split	No	Histology	No	MRI	Breast cancer
Arevalo et al. 2016	CNN3	No	736	Images	Breast Cancer Digital Repository (BCDR), Portugal	Stratified Sampling	No	Histology	No	Mammogram	Breast cancer
Bandeira Diniz et al. 2018	CNN	No	(a) 200; (b) 288	Images	(a) DDSM Dense Breast; (b) DDSM Non Dense Breast	Random split	No	Follow up, histology, expert reader	No	Mammogram	Breast cancer
Becker et al. 2017^91^	dANN	No	70	Images	Breast Cancer Digital Repository (BCDR)	Random split	Yes	Expert reader	Yes	Mammogram	Breast cancer
Becker et al. 2018^62^	DNN	No	192	Lesions	Private	Random split	No	Follow up, histology	Yes	Ultrasound	Breast cancer
Bevilacqua et al. 2019	VGG-S	No	39	Images	NR	NR	No	NR	No	Digital breast tomosynthesis	Breast cancer
Byra et al. 2019^99^	VGG-19	No	(a) 150; (b) 163; (c) 100	Images	(a) Moores Cancer Center, University of California; (b) UDIAT (c) OASBUD	Random split	No	(a) Follow up, histology; (b) expert reader; (c) expert reader, histology, follow up	Yes	Ultrasound	Breast cancer
Cai et al. 2019	CNN	No	99	Images	SYSUCC and Foshan, China	Random split	No	Histology	No	Mammogram	Breast cancer
Cao et al. 2019	SSD300 + ZFNet	No	183	Lesions	Sichuan Provincial People’s Hospital	Random split	No	Expert consensus	No	Ultrasound	Breast cancer
Cao et al. 2019	NF-Net	No	272	Lesions	Sichuan Provincial People’s Hospital	Random split	No	Histology	No	Ultrasound	Breast cancer
Cheng et al. 2016	Stacked denoising autoencoder	No	520	Lesions	Taipei Veterans General Hospital	NR	No	Histology	No	Ultrasound	Breast Nodules
Chiao et al. 2019	Mask R-CNN	No	61	Images	China Medical University Hospital	Random split	No	Histology, routine clinical report	No	Ultrasound	Breast cancer
Choi et al. 2019^100^	CNN	No	253	Lesions	Samsung Medical Centre, Seoul	NR	No	Follow up, histology	Yes	Ultrasound	Breast cancer
Chougrad et al. 2018	Inception-v3	No	(a) 5316; (b) 600; (c) 200	Images	(a) DDSM; (b) Inbreast; (c) BCDR	Random split	No	(a) Follow up, histology, expert reader; (b) expert reader, histology; (c) clinical reports	No	Mammogram	Breast cancer
Ciritsis et al. 2019^92^	dCNN	No	(a) 101; (b) 43	Images	(a) Internal validation; (b) external validation	Random split	Yes	Follow up, histology	Yes	Ultrasound	Breast cancer
Cogan et al. 2019^93^	ResNet-101 Faster R-CNN	No	124	Images	INbreast	NA	Yes	Expert reader, histology,	No	Mammogram	Breast cancer
Dalmis et al. 2018	U-Net	No	66	Images	NR	Random split	No	Follow up, histology	No	MRI	Breast cancer
Dalmis et al. 2019^101^	DenseNet	No	576	Lesions	Raboud University Medical Center	NR	No	Follow up, histology	Yes	MRI	Breast cancer
Dhungel et al. 2017	CNN	No	82	Images	INbreast	Random split	No	Expert reader, histology,	No	Mammogram	Breast cancer
Duggento et al. 2019	CNN	No	378	Images	Curated Breast Imaging SubSet of DDSM (CBIS-DDSM)	Random split	No	Expert reader	No	Mammogram	Breast cancer
Fan et al. 2019	Faster R-CNN	No	182	Images	Fudan University Affiliated Cancer Centre	Random split	No	Histology	No	Digital breast tomosynthesis	Breast cancer
Fujioka et al. 2019^102^	GoogleNet	No	120	Lesions	Private	Random split	No	Follow up, histology	Yes	Ultrasound	Breast cancer
Gao et al. 2018	SD-CNN	No	(a) 49; (b) 89	(a) Lesions; (b) images	(a) Mayo Clinic Arizona; (b) Inbreast	NR	No	(a) Histology; (b) expert reader, histology	No	(a) Contrast enhanced digital mammogram; (b) mammogram	Breast cancer
Ha et al. 2019	CNN	No	60	Images	Columbia University Medical Center	Random split	No	Follow up, histology	No	Mammogram	DCIS
Han et al. 2017	GoogleNet	No	829	Lesions	Samsung Medical Centre, Seoul	Random split	No	Histology	No	Ultrasound	Breast cancer
Herent et al. 2019	ResNet50	No	168	Lesions	Journees Francophones de Radiologie 2018	Random split	No	NR	No	MRI	Breast cancer
Hizukuri et al. 2018	CNN	No	194	Images	Mie University Hospital	Random split	No	Follow up, histology	No	Ultrasound	Breast cancer
Huyng et al. 2016	AlexNet	No	607	Images	University of Chicago	NR	No	Histology	No	Mammogram	Breast cancer
Jadoon et al. 2016	CNN-DW	No	2976	Images	IRMA	NR	No	Histology	No	Mammogram	Breast cancer
Jiao et al. 2016	CNN	No	300	Images	DDSM	Random split	No	Follow up, histology, expert reader	No	Mammogram	Breast cancer
Jiao et al. 2018	(a) AlexNet; (b) parasitic metric learning layers	No	(a) 150; (b) 150	Images	DDSM	Random split	No	Follow up, histology, expert reader	No	Mammogram	Breast cancer
Jung et al. 2018	RetinaNet	No	(a) 410; (b) 222	Images	(a) Inbreast; (b) GURO	Random split	No	(a) Expert reader; (b) histology	No	Mammogram	Breast cancer
Kim et al. 2012^103^	ANN	No	70	Lesions	Kangwon National University College of Medicine	Random split	No	Expert consensus	Yes	Ultrasound	Breast cancer
Kim et al. 2018	ResNet	No	1238	Images	Yonsei University Health System	Random split	No	Follow up, histology	No	Mammogram	Breast cancer
Kim et al. 2018	VGG-16	No	340	Images	DDSM	Hold-out method	No	Follow up, histology, expert reader	No	Mammogram	Breast cancer
Kooi et al. 2017	CNN	No	18,182	Images	Netherlands screening database	Random split	No	Expert reader, histology,	No	Mammogram	Breast cancer
Kooi et al. 2017	CNN	No	1523	Images	Netherlands screening database	Random split	No	Expert reader, histology,	No	Mammogram	Breast cancer
Kooi T et al. 2017	CNN	No	1804	Images	Netherlands screening database	Hold-out method	No	Expert reader, histology,	No	Mammogram	Breast Cancer
Li et al. 2019	DenseNet-II	No	2042	Images	First Hospital of Shanxi Medical University	Tenfold cross validation	No	Expert reader	No	Mammogram	Breast cancer
Li et al. 2019	VGG-16	No	(a) 1854; (b) 1854	Images	Nanfang Hospital	Fivefold cross validation	No	Follow up, histology	No	(a) Digital breast tomosynthesis; (b) mammogram	Breast cancer
Lin et al. 2014	FCMNN	No	65	Images	Far Eastern Memorial Hospital, Taiwan	Tenfold cross validation	No	Histology	No	Ultrasound	Breast cancer
McKinney et al. 2020^94^	MobileNetV2 - ResNet-v2-50, ResNet-v1-50	No	(a) 25,856; (b) 3097	Images	(a) UK; (b) USA	Random split	Yes	Follow up, histology	Yes	Mammogram	Breast cancer
Mendel et al. 2018	VGG-19	No	(a) 78; (b) 78	Images	University of Chicago	Leave-one-out method	No	Follow up, histology	No	(a) Mammogram; (b) digital breast tomosynthesis	Breast cancer
Peng et al. 2016^95^	ANN	No	(a) 100; (b) 100	Images	(a) MIAS; (b) BancoWeb	Hold-out method	Yes	Expert reader	No	Mammogram	Breast cancer
Qi et al. 2019	Inception-Resnet-v2	No	1359	Images	West China Hospital, Sichuan University	Random split	No	Expert consensus	No	Ultrasound	Breast cancer
Qiu et al. 2017	CNN	No	140	Images	Private	Random split	No	Histology	No	Mammogram	Breast cancer
Ragab et al. 2019	AlexNet	No	(a) 676; (b) 1581	Images	(a) Digital database for screening mammography (DDSM); (b) Curated Breast Imaging SubSet of DDSM (CBIS-DDSM)	Random split	No	Follow up, histology, expert reader	No	Mammogram	Breast cancer
Ribli et al. 2018^96^	VGG-16	No	115	Images	INbreast	NA	Yes	Expert reader, histology	No	Mammogram	Breast cancer
Rodriguez-Ruiz et al. 2018^97^	CNN	No	240	Images	Two datasets combined	NA	Yes	Expert reader, histology, follow up	Yes	Mammogram	Breast cancer
Rodriguez-Ruiz et al. 2019^98^	CNN	No	2642	Images	Combined nine datasets	NA	Yes	Follow up, histology	Yes	Mammogram	Breast cancer
Samala et al. 2016	DCNN	No	94	Images	University of Michigan	Random split	No	Expert reader	No	Digital breast tomosynthesis	Breast cancer
Samala et al. 2017	DCNN	No	907	Images	DDSM + private	Random split	No	Expert reader	No	Mammogram	Breast cancer
Samala et al. 2018	DCNN	No	94	Images	University of Michigan	Random split	No	Expert reader	No	Digital breast tomosynthesis	Breast cancer
Samala et al. 2019	AlexNet	No	94	Images	University of Michigan	Random split	No	Expert reader	No	Digital breast tomosynthesis	Breast cancer
Shen et al. 2019	(a) VGG-16; (b) ResNet; (c) ResNet-VGG	No	(a) 376; (b) 376; (c) 107	Images	(a) Curated Breast Imaging SubSet of DDSM (CBIS-DDSM); (b) Curated Breast Imaging SubSet of DDSM (CBIS-DDSM); (c) Inbreast	Random split	No	(a) Histology; (b) histology; (c) expert reader	No	Mammogram	Breast cancer
Shin et al. 2019	VGG-16	No	(a) 600; (b) 40	Images	(a) Seoul National University Bundang Hospital; (b) UDIAT Diagnostic Centre of the Parc Taulí Corporation	Random split	No	(a) NR; (b) expert reader	No	Ultrasound	Breast cancer
Stoffel et al. 2018	CNN	No	33	Images	Private	Random split	No	Surgical confirmation	Yes	Ultrasound	Phyllodes tumour
Sun et al. 2017	CNN	No	758	Images	University of Texas at El Paso	Random split	No	Expert reader	No	Mammogram	Breast cancer
Tanaka et al. 2019	VGG-19, Resnet152	No	154	Lesions	Japan Association of Breast and Thyroid Sonology	Random split	No	Histology	No	Ultrasound	Breast cancer
Tao et al. 2019	RefineNet + DenseNet121	No	253	Lesions	Huaxi Hospital and China-Japan Friendship Hospital	Random split	No	Expert reader	No	Ultrasound	Breast cancer
Teare et al. 2017	Inception-v3	No	352	Images	DDSM + Zebra Mammography Dataset	Random split	No	Follow up, histology	No	Mammogram	Breast cancer
Truhn et al. 2018^104^	CNN	No	129	Lesions	RWTH Aachen University,	Random split	No	Follow up, histology	Yes	MRI	Breast cancer
Wang et al. 2016	Inception-v3	No	74	Images	Breast Cancer Digital Repository (BCDR)	Random split	No	Expert reader, histology	No	Mammogram	Breast cancer
Wang et al. 2016	Stacked autoencoder	No	204	Images	Sun Yat-sen University Cancer Center (Guangzhou, China) and Nanhai Affiliated Hospital of Southern Medical University (Foshan, China)	Hold-out method	No	Histology	No	Mammogram	Breast cancer
Wang et al. 2017	CNN	No	292	Images	University of Chicago	Random split	No	Histology	No	Mammogram	Breast cancer
Wang et al. 2018	DNN	No	292	Images	University of Chicago	Random split	No	Histology	No	Mammogram	Breast cancer
Wu et al. 2019^105^	ResNet-22	No	(a) 401; (b) 1440	Images	NYU	Hold-out method	No	Histology	Yes	Mammogram	Breast cancer
Xiao et al. 2019	Inception-v3, ResNet50, Xception	No	206	Images	Third Affiliated Hospital of Sun Yat-sen University	Random split	No	Surgical confirmation, histology	No	Ultrasound	Breast cancer
Yala et al. 2019^106^	ResNet18	No	26,540	Images	Massachusetts General Hospital, Harvard Medical School,	Random split	No	Clinical reports, follow up, histology	Yes	Mammogram	Breast cancer
Yala et al. 2019^111^	ResNet18	No	8751	Images	Massachusetts General Hospital, Harvard Medical School,	Random split	No	Clinical reports, follow up, histology	No	Mammogram	Breast cancer
Yap et al. 2018	FCN-AlexNet	No	(a) 306; (b) 163	Lesions	(a) Private; (b) UDIAT	NR	No	Expert reader	No	Ultrasound	Breast cancer
Yap et al. 2019	FCN-8s	No	94	Lesions	Two datasets combined	NR	No	Expert reader	No	Ultrasound	Breast cancer
Yousefi et al. 2018	DCNN	No	28	Images	MGH	Random split	No	Expert consensus	No	Digital breast tomosynthesis	Breast cancer
Zhou et al. 2019^107^	3D DenseNet	No	307	Lesions	Private	Random split	No	Follow up, histology	Yes	MRI	Breast cancer

### Ophthalmology imaging

Eighty-two studies with 143 separate patient cohorts reported diagnostic accuracy data for DL in ophthalmology (see Table 2 and Supplementary References 1). Optical coherence tomography (OCT) and retinal fundus photographs (RFP) were the two imaging modalities performed in this speciality with four main pathologies being diagnosed—diabetic retinopathy (DR), age-related macular degeneration (AMD), glaucoma and retinopathy of prematurity (ROP).

Only eight studies^14–21^ used prospectively collected data and 29 (refs. ^14,15,17,18,21–45^) studies validated algorithms on external datasets. No studies provided a prespecified sample size calculation. Twenty-five studies^17,28,29,35,37,39,40,44–61^ compared algorithm performance against healthcare professionals. Reference standards, definitions of disease and threshold for diagnosis varied greatly as did the method of internal validation used. There was high heterogeneity across all studies (see Table 2).

*Diabetic retinopathy:* Twenty-five studies with 48 different patient cohorts reported diagnostic accuracy data for all, referable or vision-threatening DR on RFP. Twelve studies and 16 cohorts reported on diabetic macular oedema (DME) or early DR on OCT scans. AUC was 0.939 (95% CI 0.920–0.958) for RFP versus 1.00 (95% CI 0.999–1.000) for OCT.

*Age-related macular degeneration:* Twelve studies reported diagnostic accuracy data for features of varying severity of AMD on RFP (14 cohorts) and 11 studies in OCT (21 cohorts). AUC was 0.963 (95% CI 0.948–0.979) for RFP versus 0.969 (95% CI 0.955–0.983) for OCT.

*Glaucoma:* Seventeen studies with 30 patient cohorts reported diagnostic accuracy for features of glaucomatous optic neuropathy, optic discs or suspect glaucoma on RFP and five studies with 6 cohorts on OCT. AUC was 0.933 (95% CI 0.924–0.942) for RFP and 0.964 (95% CI 0.941–0.986) for OCT. One study^34^ with six cohorts on RFP provided contingency tables. When averaging across the cohorts, the pooled sensitivity was 0.94 (95% CI 0.92–0.96) and pooled specificity was 0.95 (95% CI 0.91–0.97). The AUC of the summary receiver-operating characteristic (SROC) curve was 0.98 (95% CI 0.96–0.99)—see Supplementary Fig. 1.

*Retinopathy of prematurity:* Three studies reported diagnostic accuracy for identifying plus diseases in ROP from RFP. Sensitivity was 0.960 (95% CI 0.913—1.008) and specificity was 0.907 (95% CI 0.907–1.066). AUC was only reported in two studies so was not pooled.

*Others:* Eight other studies reported on diagnostic accuracy in ophthalmology either using different imaging modalities (ocular images and visual fields) or for identifying other diagnoses (pseudopapilloedema, retinal vein occlusion and retinal detachment). These studies were not included in the meta-analysis.

### Respiratory imaging

One hundred and fifteen studies with 244 separate patient cohorts report on diagnostic accuracy of DL on respiratory disease (see Table 3 and Supplementary References 2). Lung nodules were largely identified on CT scans, whereas chest X-rays (CXR) were used to diagnose a wide spectrum of conditions from simply being ‘abnormal’ to more specific diagnoses, such as pneumothorax, pneumonia and tuberculosis.

Only two studies^62,63^ used prospectively collected data and 13 (refs. ^63–75^) studies validated algorithms on external data. No studies provided a prespecified sample size calculation. Twenty-one^54,63–67,70,72,76–88^ studies compared algorithm performance against healthcare professionals. Reference standards varied greatly as did the method of internal validation used. There was high heterogeneity across all studies (see Table 3).

*Lung nodules:* Fifty-six studies with 74 separate patient cohorts reported diagnostic accuracy for identifying lung nodules on CT scans on a per lesion basis, compared with nine studies and 14 patient cohorts on CXR. AUC was 0.937 (95% CI 0.924–0.949) for CT versus 0.884 (95% CI 0.842–0.925) for CXR. Seven studies reported on diagnostic accuracy for identifying lung nodules on CT scans on a per scan basis, these were not included in the meta-analysis.

*Lung cancer or mass:* Six studies with nine patient cohorts reported diagnostic accuracy for identifying mass lesions or lung cancer on CT scans compared with eight studies and ten cohorts on CXR. AUC was 0.887 (95% CI 0.847–0.928) for CT versus 0.864 (95% CI 0.827–0.901) for CXR.

*Abnormal Chest X-ray:* Twelve studies reported diagnostic accuracy for abnormal CXR with 13 different patient cohorts. AUC was 0.917 (95% CI 0.869–0.966), sensitivity was 0.873 (95% CI 0.762–0.985) and specificity was 0.894 (95% CI 0.860–0.929).

*Pneumothorax:* Ten studies reported diagnostic accuracy for pneumothorax on CXR with 14 different patient cohorts. AUC was 0.910 (95% CI 0.863–0.957), sensitivity was 0.718 (95% CI 0.433–1.004) and specificity was 0.918 (95% CI 0.870–0.965). Five patient cohorts from two studies^73,89^ provided contingency tables with raw diagnostic accuracy. When averaging across the cohorts, the pooled sensitivity was 0.70 (95% CI 0.45–0.87) and pooled specificity was 0.94 (95% CI 0.90–0.97). The AUC of the SROC curve was 0.94 (95% CI 0.92–0.96)—see Supplementary Fig. 2.

*Pneumonia:* Ten studies reported diagnostic accuracy for pneumonia on CXR with 15 different patient cohorts. AUC was 0.845 (95% CI 0.782–0.907), sensitivity was 0.951 (95% CI 0.936–0.965) and specificity was 0.716 (95% CI 0.480–0.953).

*Tuberculosis:* Six studies reported diagnostic accuracy for tuberculosis on CXR with 17 different patient cohorts. AUC was 0.979 (95% CI 0.978–0.981), sensitivity was 0.998 (95% CI 0.997–0.999) and specificity was 1.000 (95% CI 0.999–1.000). Four patient cohorts from one study^90^ provided contingency tables with raw diagnostic accuracy. When averaging across the cohorts, the pooled sensitivity was 0.95 (95% CI 0.91–0.97) and pooled specificity was 0.97 (95% CI 0.93–0.99). The AUC of the SROC curve was 0.97 (95% CI 0.96–0.99)—see Supplementary Fig. 3.

X-ray imaging was also used to identify atelectasis, pleural thickening, fibrosis, emphysema, consolidation, hiatus hernia, pulmonary oedema, infiltration, effusion, mass and cardiomegaly. CT imaging was also used to diagnose COPD, ground glass opacity and interstitial lung disease, but these were not included in the meta-analysis.

### Breast imaging

Eighty-two studies with 100 separate patient cohorts report on diagnostic accuracy of DL on breast disease (see Table 4 and Supplementary References 3). The four imaging modalities of mammography (MMG), digital breast tomosynthesis (DBT), ultrasound and magnetic resonance imaging (MRI) were used to diagnose breast cancer.

No studies used prospectively collected data and eight^91–98^ studies validated algorithms on external data. No studies provided a prespecified sample size calculation. Sixteen studies^62,91,92,94,97–107^ compared algorithm performance against healthcare professionals. Reference standards varied greatly as did the method of internal validation used. There was high heterogeneity across all studies (see Table 4).

*Breast cancer:* Forty-eight studies with 59 separate patient cohorts reported diagnostic accuracy for identifying breast cancer on MMG (AUC 0.873 [95% CI 0.853–0.894]), 22 studies and 25 patient cohorts on ultrasound (AUC 0.909 [95% CI 0.881–0.936]), and eight studies on MRI (AUC 0.868 [95% CI 0.850–0.886]) and DBT (AUC 0.908 [95% CI 0.880–0.937]).

### Other specialities

Our literature search also identified 224 studies in other medical specialities reporting on diagnostic accuracy of DL algorithms to identify disease. These included large numbers of studies in the fields of neurology/neurosurgery (78), gastroenterology/hepatology (24) and urology (25). Out of the 224 studies, only 55 compared algorithm performance against healthcare professionals, although 80% of studies in the field of dermatology did (see Supplementary References 4, Supplementary Table 1 and Supplementary Fig. 4).

### Variation of reporting

A key finding of our review was the large degree of variation in methodology, reference standards, terminology and reporting among studies in all specialities. The most common variables amongst DL studies in medical imaging include issues with the quality and size of datasets, metrics used to report performance and methods used for validation (see Table 5). Only eight studies in ophthalmology imaging^14,21,32,33,43,55,108,109^, ten studies in respiratory imaging^64,66,70,72,75,79,82,87,89,110^ and six studies in breast imaging^62,91,97,104,106,111^ mentioned adherence to the STARD-2015 guidelines or had a STARD flow diagram in the manuscript.

**Table 5:** Variation in DL imaging studies.

*Data*	
Image pre-processing, augmentation and preparation	Are data augmentation techniques such as cropping, padding and flipping used? Is there quality control of the images being used to train the algorithm? I.e., were poor quality images excluded. Were relevant images manually selected?
**Study design**	**Retrospective or prospective data collection.**
**Image eligibility**	**How are images chosen for inclusion in the study?** **Were the data from private or open-access repositories?**
Training, validation, test sets	Are each of the three sets independent of each other, without overlap? Does data from the same patient appear in multiple datasets?
**Datasets**	**Are the datasets used single or multicentre?** **Is a public or open-source dataset used?**
**Size of datasets**	**Wide variation in size of datasets for training and testing.** **Is the size of the datasets justified?** **Are sample size statistical considerations applied for the test set?**
Use of ‘external’ test sets for final reporting	Is an independent test set used for ‘external validation’? Is the independent test set constructed using an unenriched representative sample?
Multi-vendor images	Are images from different scanners and vendors included in the datasets to enhance generalisability? Are imaging acquisition parameters described?
*Algorithm*	
**Index test**	**Was sufficient detail given on the algorithm to allow replication and independent validation?** **What type of algorithm was used? E.g., CNN, Autoencoder, SVM.** **Was the algorithm made publicly or commercially available?** **Was the construct or architecture of the algorithm made available?**
Additional AI algorithmic information	Is the algorithm a static model or is it continuously evolving?
Demonstrate how algorithm makes decisions	Is there a specific design for end-user interpretability, e.g., saliency or probability maps
*Methods*	
Transfer learning	Was transfer learning used for training and validation?
Cross validation	Was k-fold cross validation used during training to reduce the effects of randomness in dataset splits?
**Reference standard**	**Is the reference standard used of high quality and widely accepted in the field?** **What was the rationale for choosing the reference standard?**
**Additional clinical information**	**Was additional clinical information given to healthcare professionals to simulate normal clinical process?**
Performance benchmarking	What was performance of algorithm benchmarked to? What is expertise level and level of consensus of healthcare professionals if used?
*Results*	
**Raw diagnostic accuracy data**	**Are raw diagnostic accuracy data reported in a contingency table demonstrating TP, FP, FN, TN?**
**Metrics for estimating diagnostic accuracy performance**	**Which diagnostic accuracy metrics reported? Sensitivity, Sensitivity, PPV, NPV, Accuracy, AUROC**
**Unit of assessment**	**Which unit of assessment reported, e.g., per patient, per scan or per lesion.**

Rows in bold are part of STARD-2015 criteria.

Funnel plots were produced for the diagnostic accuracy outcome measure with the largest number of patient cohorts in each medical speciality, in order to detect bias in the studies included^112^ (see Supplementary Figs. 5–7). These demonstrate that there is high risk of bias in studies detecting lung nodules on CT scans and detecting DR on RFP, but not for detecting breast cancer on MMG.

### Assessment of the validity and applicability of the evidenc

**Fig. 2:**
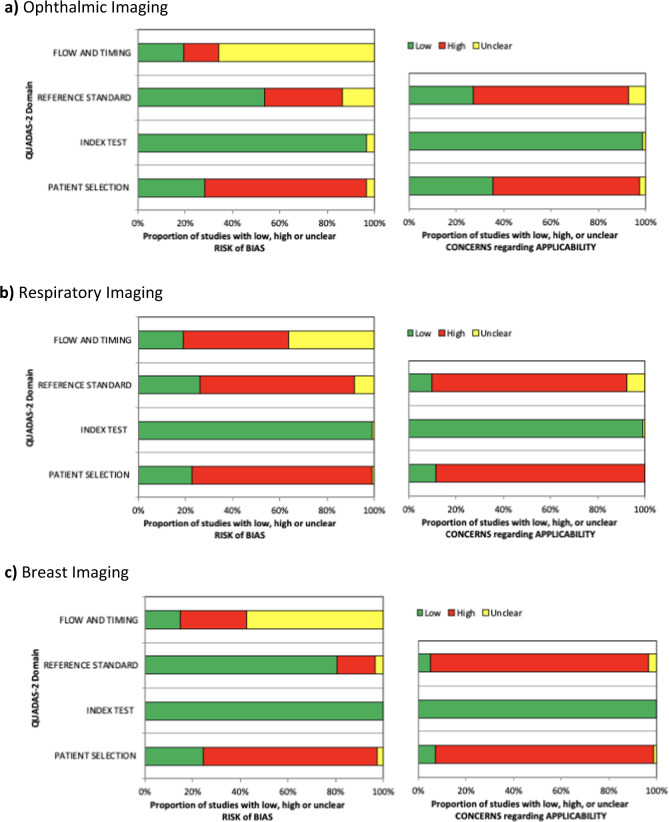
QUADAS-2 summary plots. Risk of bias and applicability concerns summary about each QUADAS-2 domain presented as percentages across the 82 included studies in ophthalmic imaging (**a**), 115 in respiratory imaging (**b**) and 82 in breast imaging (**c**).

The overall risk of bias and applicability using Quality Assessment of Diagnostic Accuracies Studies 2 (QUADAS-2) led to a majority of studies in all specialities being classified as high risk, particularly with major deficiencies in regard to patient selection, flow and timing and applicability of the reference standard (see Fig. 2). For the patient selection domain, a high or unclear risk of bias was seen in 59/82 (72%) of ophthalmic studies, 89/115 (77%) of respiratory studies and 62/82 (76%) or breast studies. These were mostly related to a case–control study design and sampling issues. For the flow and timing domain, a high or unclear risk of bias was seen in 66/82 (80%) of ophthalmic studies, 93/115 (81%) of respiratory studies and 70/82 (85%) of breast studies. This was largely due to missing information about patients not receiving the index test or whether all patients received the same reference standard. For the reference standard domain, concerns regarding applicability was seen in 60/82 (73%) of ophthalmic studies, 104/115 (90%) of respiratory studies and 78/82 (95%) of breast studies. This was mostly due to reference standard inconsistencies if the index test was validated on external datasets.

## Discussion

This study sought to (1) quantify the diagnostic accuracy of DL algorithms to identify specific pathology across distinct radiological modalities, and (2) appraise the variation in study reporting of DL-based radiological diagnosis. The findings of our speciality-specific meta-analysis suggest that DL algorithms generally have a high and clinically acceptable diagnostic accuracy in identifying disease. High diagnostic accuracy with analogous DL approaches was identified in all specialities despite different workflows, pathology and imaging modalities, suggesting that DL algorithms can be deployed across different areas in radiology. However, due to high heterogeneity and variance between studies, there is considerable uncertainty around estimates of diagnostic accuracy in this meta-analysis.

In ophthalmology, the findings suggest features of diseases, such as DR, AMD and glaucoma can be identified with a high sensitivity, specificity and AUC, using DL on both RFP and OCT scans. In general, we found higher sensitivity, specificity, accuracy and AUC with DL on OCT scans over RFP for DR, AMD and glaucoma. Only sensitivity was higher for DR on RFP over OCT.

In respiratory medicine, our findings suggest that DL has high sensitivity, specificity and AUC to identify chest pathology on CT scans and CXR. DL on CT had higher sensitivity and AUC for detecting lung nodules; however, we found a higher specificity, PPV and *F*1 score on CXR. For diagnosing cancer or lung mass, DL on CT had a higher sensitivity than CXR.

In breast cancer imaging, our findings suggest that DL generally has a high diagnostic accuracy to identify breast cancer on mammograms, ultrasound and DBT. The performance was found to be very similar for these modalities. In MRI, however, the diagnostic accuracy was lower; this may be due to small datasets and the use of 2D images. The utilisation of larger databases and multiparametric MRI may increase the diagnostic accuracy^113^.

Extensive variation in the methodology, data interpretability, terminology and outcome measures could be explained by a lack of consensus in how to conduct and report DL studies. The STARD-2015 checklist^114^, designed for reporting of diagnostic accuracy studies is not fully applicable to clinical DL studies^115^. The variation in reporting makes it very difficult to formally evaluate the performance of algorithms. Furthermore, differences in reference standards, grader capabilities, disease definitions and thresholds for diagnosis make direct comparison between studies and algorithms very difficult. This can only be improved with well-designed and executed studies that explicitly address questions concerning transparency, reproducibility, ethics and effectiveness^116^ and specific reporting standards for AI studies^115,117^.

The QUADAS-2 (ref. ^118^) assessment tool was used to systematically evaluate the risk of bias and any applicability concerns of the diagnostic accuracy studies. Although this tool was not designed for DL diagnostic accuracy studies, the evaluation allowed us to judge that a majority of studies in this field are at risk of bias or concerning for applicability. Of particular concern was the applicability of reference standards and patient selection.

Despite our results demonstrating that DL algorithms have a high diagnostic accuracy in medical imaging, it is currently difficult to determine if they are clinically acceptable or applicable. This is partially due to the extensive variation and risk of bias identified in the literature to date. Furthermore, the definition of what threshold is acceptable for clinical use and tolerance for errors varies greatly across diseases and clinical scenarios^119^.

### Limitations in the literature

#### Dataset

There are broad methodological deficiencies among the included studies. Most studies were performed using retrospectively collected data, using reference standards and labels that were not intended for the purposes of DL analysis. Minimal prospective studies and only two randomised studies^109,120^, evaluating the performance of DL algorithms in clinical settings were identified in the literature. Proper acquisition of test data is essential to interpret model performance in a real-world clinical setting. Poor quality reference standards may result in the decreased model performance due to suboptimal data labelling in the validation set^28^, which could be a barrier to understanding the true capabilities of the model on the test set. This is symptomatic of the larger issue that there is a paucity of gold-standard, prospectively collected, representative datasets for the purposes of DL model testing. However, as there are many advantages to using retrospectively collected data, the resourceful use of retrospective or synthetic data with the use of labels of varying modality and quality represent important areas of research in DL^121^.

#### Study methodology

Many studies did not undertake external validation of the algorithm in a separate test set and relied upon results from the internal validation data; the same dataset used to train the algorithm initially. This may lead to an overestimation of the diagnostic accuracy of the algorithm. The problem of overfitting has been well described in relation to machine learning algorithms^122^. True demonstration of the performance of these algorithms can only be assumed if they are externally validated on separate test sets with previously unseen data that are representative of the target population.

Surprisingly, few studies compared the diagnostic accuracy of DL algorithms against expert human clinicians for medical imaging. This would provide a more objective standard that would enable better comparison of models across studies. Furthermore, application of the same test dataset for diagnostic performance assessment of DL algorithms versus healthcare professionals was identified in only select studies^13^. This methodological deficiency limits the ability to gauge the clinical applicability of these algorithms into clinical practice. Similarly, this issue can extend to model-versus-model comparisons. Specific methods of model training or model architecture may not be described well enough to permit emulation for comparison^123^. Thus, standards for model development and comparison against controls will be needed as DL architectures and techniques continue to develop and are applied in medical contexts.

#### Reporting

There was varying terminology and a lack of transparency used in DL studies with regards to the validation or test sets used. The term ‘validation’ was identified as being used interchangeably to either describe an external test set for the final algorithm or for an internal dataset that is used to fine tune the model prior to ‘testing’. Furthermore, the inconsistent terminology led to difficulties understanding whether an independent external test set was used to test diagnostic performance^13^.

Crucially, we found broad variation in the metrics used as outcomes for the performance of the DL algorithms in the literature. Very few studies reported true positives, false positives, true negatives and false negatives in a contingency table as should be the minimum for diagnostic accuracy studies^114^. Moreover, some studies only reported metrics, such as dice coefficient, *F*1 score, competition performance metric and Top-1 accuracy that are often used in computer science, but may be unfamiliar to clinicians^13^. Metrics such as AUC, sensitivity, specificity, PPV and NPV should be reported, as these are more widely understood by healthcare professionals. However, it is noted that NPV and PPV are dependent on the underlying prevalence of disease and as many test sets are artificially constructed or balanced, then reporting the NPV or PPV may not be valid. The wide range of metrics reported also leads to difficulty in comparing the performance of algorithms on similar datasets.

### Study strengths and limitations

This systematic review and meta-analysis statistically appraises pooled data collected from 279 studies. It is the largest study to date examining the diagnostic accuracy of DL on medical imaging. However, our findings must be viewed in consideration of several limitations. Firstly, as we believe that many studies have methodological deficiencies or are poorly reported, these studies may not be a reliable source for evaluating diagnostic accuracy. Consequently, the estimates of diagnostic performance provided in our meta-analysis are uncertain and may represent an over-estimation of the true accuracy. Secondly, we did not conduct a quality assessment for the transparency of reporting in this review. This was because current guidelines to assess diagnostic accuracy reporting standards (STARD-2015^114^) were not designed for DL studies and are not fully applicable to the specifics and nuances of DL research^115^. Thirdly, due to the nature of DL studies, we were not able to perform classical statistical comparison of measures of diagnostic accuracy between different imaging modalities. Fourthly, we were unable to separate each imaging modality into different subsets, to enable comparison across subsets and allow the heterogeneity and variance to be broken down. This was because our study aimed to provide an overview of the literature in each specific speciality, and it was beyond the scope of this review to examine each modality individually. The inherent differences in imaging technology, patient populations, pathologies and study designs meant that attempting to derive common lessons across the board did not always offer easy comparisons. Finally, our review concentrated on DL for speciality-specific medical imaging, and therefore it may not be appropriate to generalise our findings to other forms of medical imaging or AI studies.

### Future work

For the quality of DL research to flourish in the future, we believe that the adoption of the following recommendations are required as a starting point.

#### Availability of large, open-source, diverse anonymised datasets with annotations

This can be achieved through governmental support and will enable greater reproducibility of DL models^124^.

#### Collaboration with academic centres to utilise their expertise in pragmatic trial design and methodology^125^

Rather than classical trials, novel experimental and quasi-experimental methods to evaluate DL have been proposed and should be evaluated^126^. This may include ongoing evaluation of algorithms once in clinical practice, as they continue to learn and adapt to the population that they are implemented in.

#### Creation of AI-specific reporting standards

A major reason for the difficulties encountered in evaluating the performance of DL on medical imaging are largely due to inconsistent and haphazard reporting. Although DL is widely considered as a ‘predictive’ model (where TRIPOD may be applied) the majority of AI interventions close to translation currently published are predominantly in the field of diagnostics (with specifics on index tests, reference standards and true/false positive/negatives and summary diagnostic scores, centred directly in the domain of STARD). Existing reporting guidelines for diagnostic accuracy studies (STARD)^114^, prediction models (TRIPOD)^127^, randomised trials (CONSORT)^128^ and interventional trial protocols (SPIRIT)^129^ do not fully cover DL research due to specific considerations in methodology, data and interpretation required for these studies. As such, we applaud the recent publication of the CONSORT-AI^117^ and SPIRIT-AI^130^ guidelines, and await AI-specific amendments of the TRIPOD-AI^131^ and STARD-AI^115^ statements (which we are convening). We trust that when these are published, studies being conducted will have a framework that enables higher quality and more consistent reporting.

#### Development of specific tools for determining the risk of study bias and applicability

An update to the QUADAS-2 tool taking into account the nuances of DL diagnostic accuracy research should be considered.

#### Updated specific ethical and legal framework

Outdated policies need to be updated and key questions answered in terms of liability in cases of medical error, doctor and patient understanding, control over algorithms and protection of medical data^132^. The World Health Organisation^133^ and others have started to develop guidelines and principles to regulate the use of AI. These regulations will need to be adapted by each country to fit their own political and healthcare context^134^. Furthermore, these guidelines will need to proactively and objectively evaluate technology to ensure best practices are developed and implemented in an evidence-based manner^135^.

## Conclusion

DL is a rapidly developing field that has great potential in all aspects of healthcare, particularly radiology. This systematic review and meta-analysis appraised the quality of the literature and provided pooled diagnostic accuracy for DL techniques in three medical specialities. While the results demonstrate that DL currently has a high diagnostic accuracy, it is important that these findings are assumed in the presence of poor design, conduct and reporting of studies, which can lead to bias and overestimating the power of these algorithms. The application of DL can only be improved with standardised guidance around study design and reporting, which could help clarify clinical utility in the future. There is an immediate need for the development of AI-specific STARD and TRIPOD statements to provide robust guidance around key issues in this field before the potential of DL in diagnostic healthcare is truly realised in clinical practice.

## Methods

This systematic review was conducted in accordance with the guidelines for the ‘Preferred Reporting Items for Systematic Reviews and Meta-Analyses’ extension for diagnostic accuracy studies statement (PRISMA-DTA)^136^.

### Eligibility criteria

Studies that report upon the diagnostic accuracy of DL algorithms to investigate pathology or disease on medical imaging were sought. The primary outcome was various diagnostic accuracy metrics. Secondary outcomes were study design and quality of reporting.

### Data sources and searches

Electronic bibliographic searches were conducted in Medline and EMBASE up to 3rd January 2020. MESH terms and all-field search terms were searched for ‘neural networks’ (DL or convolutional or cnn) and ‘imaging’ (magnetic resonance or computed tomography or OCT or ultrasound or X-ray) and ‘diagnostic accuracy metrics’ (sensitivity or specificity or AUC). For the full search strategy, please see Supplementary Methods 1. The search included all study designs. Further studies were identified through manual searches of bibliographies and citations until no further relevant studies were identified. Two investigators (R.A. and V.S.) independently screened titles and abstracts, and selected all relevant citations for full-text review. Disagreement regarding study inclusion was resolved by discussion with a third investigator (H.A.).

### Inclusion criteria

Studies that comprised a diagnostic accuracy assessment of a DL algorithm on medical imaging in human populations were eligible. Only studies that stated either diagnostic accuracy raw data, or sensitivity, specificity, AUC, NPV, PPV or accuracy data were included in the meta-analysis. No limitations were placed on the date range and the last search was performed in January 2020.

### Exclusion criteria

Articles were excluded if the article was not written in English. Abstracts, conference articles, pre-prints, reviews and meta-analyses were not considered because an aim of this review was to appraise the methodology, reporting standards and quality of primary research studies being published in peer-reviewed journals. Studies that investigated the accuracy of image segmentation or predicting disease rather than identification or classification were excluded.

### Data extraction and quality assessment

Two investigators (R.A. and V.S.) independently extracted demographic and diagnostic accuracy data from the studies, using a predefined electronic data extraction spreadsheet. The data fields were chosen subsequent to an initial scoping review and were, in the opinion of the investigators, sufficient to fulfil the aims of this review. Data were extracted on (i) first author, (ii) year of publication, (iii) type of neural network, (iv) population, (v) dataset—split into training, validation and test sets, (vi) imaging modality, (vii) body system/disease, (viii) internal/external validation methods, (ix) reference standard, (x) diagnostic accuracy raw data—true and false positives and negatives, (xi) percentages of AUC, accuracy, sensitivity, specificity, PPV, NPV and other metrics reported.

Three investigators (R.A., V.S. and GM) assessed study methodology using the QUADAS-2 checklist to evaluate the risk of bias and any applicability concerns of the studies^118^.

### Data synthesis and analysis

A bivariate model for diagnostic meta-analysis was used to calculate summary estimates of sensitivity, specificity and AUC data^137^. Independent proportion and their differences were calculated and pooled through DerSimonian and Laird random-effects modelling^138^. This considered both between-study and within-study variances that contributed to study weighting. Study-specific estimates and 95% CIs were computed and represented on forest plots. Heterogeneity between studies was assessed using *I*^*2*^ (25–49% was considered to be low heterogeneity, 50–74% was moderate and >75% was high heterogeneity). Where raw diagnostic accuracy data were available, the SROC model was used to evaluate the relationship between sensitivity and specificity^139^. We utilised Stata version 15 (Stata Corp LP, College Station, TX, USA) for all statistical analyses.

We chose to appraise the performance of DL algorithms to identify individual disease or pathology patterns on different imaging modalities in isolation, e.g., identifying lung nodules on a thoracic CT scan. We felt that combining imaging modalities and diagnoses would add heterogeneity and variation to the analysis. Meta-analysis was only performed where there were greater than or equal to three patient cohorts, reporting for each specific pathology and imaging modality. This study is registered with PROSPERO, CRD42020167503.

### Reporting summary

Further information on research design is available in the Nature Research Reporting Summary linked to this article.

## Supplementary information

Supplementary Information

Reporting Summary

## Data Availability

The authors declare that all the data included in this study are available within the paper and its Supplementary Information files.
