# Supplementary material for: Diagnostic accuracy of deep learning in medical imaging: a systematic review and meta-analysis
Source: NPJ Digit Med. 2021 Apr 7;4:65. doi: 10.1038/s41746-021-00438-z (PMC8027892; doi:10.1038/s41746-021-00438-z)
Supplement: Supplementary file 1 — Supplementary Information [file 41746_2021_438_MOESM1_ESM.pdf]

Supplementary Information

Diagnostic accuracy of deep learning in medical imaging: a systematic review and meta-analysis

Supplementary Methods 1: Literature search strategies
Supplementary References : Full list of included studies in ophthalmic imaging
Supplementary References 2: Full list of included studies in respiratory imaging
Supplementary References 3: Full list of included studies in breast imaging
Supplementary References 4: Full list of included studies in all other specialities
Supplementary Table 1: Characteristics of included studies from all other specialities
Supplementary Figure 1: Summary ROC curve for six different patient cohorts to diagnose glaucoma on retinal fundus photographs from Liu et al. 2019
Supplementary Figure 2: Summary ROC curve for five different patient cohorts to diagnose pneumothorax on CXR from Taylor et al. 2018 and Park et al. 2019
Supplementary Figure 3: Summary ROC curve for four different patient cohorts to diagnose tuberculosis on CXR from Lakhani et al. 2017
Supplementary Figure 4: Histogram demonstrating number of studies comparing algorithm performance against health-care professionals in each speciality
Supplementary Figure 5: Funnel Plot for all cohorts reporting on the sensitivity of deep learning to identify lung nodules on CT scans
Supplementary Figure 6: Funnel Plot for all cohorts reporting on the AUC of deep learning to identify breast cancer on mammography
Supplementary Figure 7: Funnel Plot for all cohorts reporting on the sensitivity of deep learning to identify diabetic retinopathy on retinal fundus photography
Supplementary Figure 8: Pooled AUC for diagnosing features of diabetic retinopathy on retinal fundus photography
Supplementary Figure 9: Pooled sensitivity for diagnosing features of diabetic retinopathy on retinal fundus photography
Supplementary Figure 10: Pooled specificity for diagnosing features of diabetic retinopathy on retinal fundus photography
Supplementary Figure 11: Pooled accuracy for diagnosing features of diabetic retinopathy on retinal fundus photography
Supplementary Figure 12: Pooled PPV for diagnosing features of diabetic retinopathy on retinal fundus photography
Supplementary Figure 13 – Pooled NPV for diagnosing features of diabetic retinopathy on retinal fundus photography
Supplementary Figure 14 – Pooled AUC for diagnosing features of age-related macular degeneration on retinal fundus photography
Supplementary Figure 15 – Pooled sensitivity for diagnosing features of age-related macular degeneration on retinal fundus photography
Supplementary Figure 16 – Pooled specificity for diagnosing features of age-related macular degeneration on retinal fundus photography
Supplementary Figure 17 – Pooled accuracy for diagnosing features of age-related macular degeneration on retinal fundus photography
Supplementary Figure 18 – Pooled AUC for diagnosing features of glaucoma on retinal fundus photography
Supplementary Figure 19 – Pooled sensitivity for diagnosing features of glaucoma on retinal fundus photography
Supplementary Figure 20 – Pooled specificity for diagnosing features of glaucoma on retinal fundus photography
Supplementary Figure 21 – Pooled accuracy for diagnosing features of glaucoma on retinal fundus photography

Supplementary Figure 22 – Pooled sensitivity for diagnosing features of plus disease in retinopathy of prematurity on retinal fundus photography
Supplementary Figure 23 – Pooled specificity for diagnosing features of plus disease in retinopathy of prematurity on retinal fundus photography
Supplementary Figure 24 – Pooled AUC for diagnosing features of diabetic retinopathy on OCT scans
Supplementary Figure 25 – Pooled sensitivity for diagnosing features of diabetic retinopathy on OCT scans
Supplementary Figure 26– Pooled specificity for diagnosing features of diabetic retinopathy on OCT scans
Supplementary Figure 27 – Pooled accuracy for diagnosing features of diabetic retinopathy on OCT scans
Supplementary Figure 28 – Pooled AUC for diagnosing features of age-related macular degeneration on OCT scans
Supplementary Figure 29 – Pooled sensitivity for diagnosing features of age-related macular degeneration on OCT scans
Supplementary Figure 30 – Pooled specificity for diagnosing features of age-related macular degeneration on OCT scans
Supplementary Figure 31 – Pooled accuracy for diagnosing features of age-related macular degeneration on OCT scans
Supplementary Figure 32 – Pooled AUC for diagnosing features of glaucoma on OCT scans
Supplementary Figure 33 – Pooled AUC for diagnosing lung nodules on CT scans
Supplementary Figure 34 – Pooled sensitivity for diagnosing lung nodules on CT scans
Supplementary Figure 35 – Pooled specificity for diagnosing lung nodules on CT scans
Supplementary Figure 36 – Pooled accuracy for diagnosing lung nodules on CT scans
Supplementary Figure 37 – Pooled PPV for diagnosing lung nodules on CT scans
Supplementary Figure 38 – Pooled F1 score for diagnosing lung nodules on CT scans
Supplementary Figure 39 – Pooled AUC for diagnosing lung cancer on CT scans
Supplementary Figure 40 – Pooled sensitivity for diagnosing lung cancer on CT scans
Supplementary Figure 41 – Pooled specificity for diagnosing lung cancer on CT scans
Supplementary Figure 42 – Pooled accuracy for diagnosing lung cancer on CT scans
Supplementary Figure 43 – Pooled AUC for diagnosing abnormal Chest X-rays
Supplementary Figure 44 – Pooled sensitivity for diagnosing abnormal Chest X-rays
Supplementary Figure 45 – Pooled specificity for diagnosing abnormal Chest X-rays
Supplementary Figure 46 – Pooled accuracy for diagnosing abnormal Chest X-rays
Supplementary Figure 47 – Pooled PPV for diagnosing abnormal Chest X-rays
Supplementary Figure 48 – Pooled F1 score for diagnosing abnormal Chest X-rays
Supplementary Figure 49 – Pooled AUC for diagnosing atelectasis on Chest X-ray
Supplementary Figure 50 – Pooled AUC for diagnosing cardiomegaly on Chest X-ray
Supplementary Figure 51 – Pooled AUC for diagnosing consolidation on Chest X-ray
Supplementary Figure 52 – Pooled sensitivity for diagnosing consolidation on Chest X-ray
Supplementary Figure 53 – Pooled specificity for diagnosing consolidation on Chest X-ray
Supplementary Figure 54 – Pooled accuracy for diagnosing consolidation on Chest X-ray
Supplementary Figure 55 – Pooled AUC for diagnosing edema on Chest X-ray
Supplementary Figure 56 – Pooled AUC for diagnosing effusion on Chest X-ray
Supplementary Figure 57 – Pooled AUC for diagnosing emphysema on Chest X-ray
Supplementary Figure 58 – Pooled AUC for diagnosing fibrosis on Chest X-ray
Supplementary Figure 59 – Pooled AUC for diagnosing hiatus hernia on Chest X-ray
Supplementary Figure 60 – Pooled AUC for diagnosing infiltration on Chest X-ray
Supplementary Figure 61 – Pooled AUC for diagnosing a mass on Chest X-ray
Supplementary Figure 62 – Pooled sensitivity for diagnosing a mass on Chest X-ray
Supplementary Figure 63 – Pooled AUC for diagnosing lung nodules on Chest X-ray
Supplementary Figure 64 – Pooled sensitivity for diagnosing lung nodules on Chest X-ray
Supplementary Figure 65 – Pooled specificity for diagnosing lung nodules on Chest X-ray
Supplementary Figure 66 – Pooled PPV for diagnosing lung nodules on Chest X-ray
Supplementary Figure 67 – Pooled F1 score for diagnosing lung nodules on Chest X-ray

Supplementary Figure 68 – Pooled AUC for diagnosing pleural thickening on Chest X-ray
Supplementary Figure 69 – Pooled AUC for diagnosing pneumonia on Chest X-ray
Supplementary Figure 70 – Pooled sensitivity for diagnosing pneumonia on Chest X-ray
Supplementary Figure 71 – Pooled specificity for diagnosing pneumonia on Chest X-ray
Supplementary Figure 72 – Pooled accuracy for diagnosing pneumonia on Chest X-ray
Supplementary Figure 73 – Pooled PPV for diagnosing pneumonia on Chest X-ray
Supplementary Figure 74 – Pooled F1 score for diagnosing pneumonia on Chest X-ray
Supplementary Figure 75 – Pooled AUC for diagnosing pneumothorax on Chest X-ray
Supplementary Figure 76 – Pooled sensitivity for diagnosing pneumothorax on Chest X-ray
Supplementary Figure 77 – Pooled specificity for diagnosing pneumothorax on Chest X-ray
Supplementary Figure 78 – Pooled PPV for diagnosing pneumothorax on Chest X-ray
Supplementary Figure 79 – Pooled AUC for diagnosing tuberculosis on Chest X-ray
Supplementary Figure 80 – Pooled sensitivity for diagnosing tuberculosis on Chest X-ray
Supplementary Figure 81 – Pooled specificity for diagnosing tuberculosis on Chest X-ray
Supplementary Figure 82– Pooled accuracy for diagnosing tuberculosis on Chest X-ray
Supplementary Figure 83 – Pooled AUC for diagnosing breast cancer on mammogram
Supplementary Figure 84 – Pooled sensitivity for diagnosing breast cancer on mammogram
Supplementary Figure 85 – Pooled specificity for diagnosing breast cancer on mammogram
Supplementary Figure 86 – Pooled accuracy for diagnosing breast cancer on mammogram
Supplementary Figure 87 – Pooled AUC for diagnosing breast cancer on ultrasound
Supplementary Figure 88 – Pooled sensitivity for diagnosing breast cancer on ultrasound
Supplementary Figure 89 – Pooled specificity for diagnosing breast cancer on ultrasound
Supplementary Figure 90 – Pooled accuracy for diagnosing breast cancer on ultrasound
Supplementary Figure 91 – Pooled F1 score for diagnosing breast cancer on ultrasound
Supplementary Figure 92 – Pooled PPV for diagnosing breast cancer on ultrasound
Supplementary Figure 93 – Pooled NPV for diagnosing breast cancer on ultrasound
Supplementary Figure 94 – Pooled AUC for diagnosing breast cancer on MRI
Supplementary Figure 95 – Pooled sensitivity for diagnosing breast cancer on MRI
Supplementary Figure 96 – Pooled specificity for diagnosing breast cancer on MRI
Supplementary Figure 97 – Pooled AUC for diagnosing breast cancer on digital breast tomosynthesis
Supplementary Figure 98 – Pooled sensitivity for diagnosing breast cancer on digital breast tomosynthesis
Supplementary Figure 99 – Pooled accuracy for diagnosing breast cancer on digital breast tomosynthesis

Supplementary Methods 1: Literature search strategies

We show the search strategy for a) Medline and b) EMBASE

a) Medline

1 artificial intelligence/ or machine learning/ or deep learning/ or supervised machine learning/ or unsupervised machine learning/ or "neural networks (computer)"/
2 (deep learning or convolutional or cnn or neural network*).mp. [mp=title, abstract, original title, name of substance word, subject heading word, floating sub-heading word, keyword heading word, organism supplementary concept word, protocol supplementary concept word, rare disease supplementary concept word, unique identifier, synonyms]
3 1 or 2
4 Magnetic Resonance Imaging/
5 exp Tomography, X-Ray Computed/
6 Tomography, Optical Coherence/
7 exp Radiography/
8 exp Ultrasonography/
9 (oct or sonogra* or Optical Coherence Tomography or mri or magnetic resonance or ct or computed tomography or ultrasound* or xray or x-ray or mammogra* or radiograph* or photograph*).mp. [mp=title, abstract, original title, name of substance word, subject heading word, floating sub-heading word, keyword heading word, organism supplementary concept word, protocol supplementary concept word, rare disease supplementary concept word, unique identifier, synonyms]
10 4 or 5 or 6 or 7 or 8 or 9
11 exp "sensitivity and specificity"/ or exp roc curve/
12 (area under the curve or roc or auc or accurac* or sensitivity or specificity or diagnostic accuracy).mp. [mp=title, abstract, original title, name of substance word, subject heading word, floating sub-heading word, keyword heading word, organism supplementary concept word, protocol supplementary concept word, rare disease supplementary concept word, unique identifier, synonyms]
13 11 or 12
14 3 and 10 and 13

b) EMBASE

1 artificial neural network/
2 (deep learning or convolutional or cnn or neural network*).mp. [mp=title, abstract, heading word, drug trade name, original title, device manufacturer, drug manufacturer, device trade name, keyword, floating subheading word, candidate term word]
3 1 or 2
4 exp nuclear magnetic resonance imaging/
5 optical coherence tomography/
6 exp radiography/
7 exp computer assisted tomography/
8 ultrasound/
9 echography/
10 (oct or Optical Coherence Tomography or mri or magnetic resonance or ct or computed tomography or ultrasound* or xray or x-ray or mammogra* or radiograph* or photograph*).mp. [mp=title, abstract, heading word, drug trade name, original title, device manufacturer, drug manufacturer, device trade name, keyword, floating subheading word, candidate term word]
11 4 or 5 or 6 or 7 or 8 or 9 or 10
12 exp "sensitivity and specificity"/
13 exp area under the curve/
14 (area under the curve or roc or auc or accurac* or sensitivity or specificity or diagnostic accuracy).mp. [mp=title, abstract, heading word, drug trade name, original title, device manufacturer, drug manufacturer, device trade name, keyword, floating subheading word, candidate term word]
15 12 or 13 or 14
16 3 and 11 and 15

Supplementary References 1: Full list of included studies in ophthalmic imaging

1. Abràmoff MD, Lavin PT, Birch M, Shah N, Folk JC. Pivotal trial of an autonomous AI-based diagnostic system for detection of diabetic retinopathy in primary care offices. Npj Digital Medicine. 2018;1(1):39.
2. Abramoff MD, Lou Y, Erginay A, et al. Improved Automated Detection of Diabetic Retinopathy on a Publicly Available Dataset Through Integration of Deep Learning. Investigative ophthalmology & visual science. 2016;57(13):5200-5206.
3. Ahn JM, Kim S, Ahn K-S, Cho S-H, Kim US. Accuracy of machine learning for differentiation between optic neuropathies and pseudopapilledema. BMC Ophthalmology. 2019;19(1):178.
4. Ahn JM, Kim S, Ahn KS, Cho SH, Lee KB, Kim US. A deep learning model for the detection of both advanced and early glaucoma using fundus photography. PloS One. 2018;13(11):e0207982.
5. Al-Aswad LA, Kapoor R, Chu CK, et al. Evaluation of a Deep Learning System For Identifying Glaucomatous Optic Neuropathy Based on Color Fundus Photographs. J Glaucoma. 2019;28(12):1029-1034.
6. Alqudah AM. AOCT-NET: a convolutional network automated classification of multiclass retinal diseases using spectral-domain optical coherence tomography images. Medical & biological engineering & computing. 2019.
7. Arcadu F, Benmansour F, Maunz A, et al. Deep Learning Predicts OCT Measures of Diabetic Macular Thickening From Color Fundus PhotographsDeep Learning and Diabetic Macular Edema. Investigative ophthalmology & visual science. 2019;60(4):852-857.
8. Asaoka R, Murata H, Hirasawa K, et al. Using Deep Learning and Transfer Learning to Accurately Diagnose Early-Onset Glaucoma From Macular Optical Coherence Tomography Images. American Journal of Ophthalmology. 2019;198:136-145.
9. Asaoka R, Murata H, Iwase A, Araie M. Detecting Preperimetric Glaucoma with Standard Automated Perimetry Using a Deep Learning Classifier. Ophthalmology. 2016;123(9):1974-1980.
10. Asaoka R, Tanito M, Shibata N, et al. Validation of a Deep Learning Model to Screen for Glaucoma Using Images from Different Fundus Cameras and Data Augmentation. Ophthalmology Glaucoma. 2019;2(4):224-231.
11. Bellemo V, Lim ZW, Lim G, et al. Artificial intelligence using deep learning to screen for referable and vision-threatening diabetic retinopathy in Africa: a clinical validation study. The Lancet Digital Health. 2019;1(1):e35-e44.
12. Bhatia KK, Graham MS, Terry L, et al. DISEASE CLASSIFICATION OF MACULAR OPTICAL COHERENCE TOMOGRAPHY SCANS USING DEEP LEARNING SOFTWARE: Validation on Independent, Multicenter Data. Retina. 2019.
13. Brown JM, Campbell JP, Beers A, et al. Automated diagnosis of plus disease in retinopathy of prematurity using deep convolutional neural networks. JAMA ophthalmology. 2018;136(7):803-810.
14. Burlina P, Billings S, Joshi N, Albayda J. Automated diagnosis of myositis from muscle ultrasound: Exploring the use of machine learning and deep learning methods. PLOS ONE. 2017;12(8):e0184059.
15. Burlina P, Joshi N, Pacheco KD, Freund DE, Kong J, Bressler NM. Utility of Deep Learning Methods for Referability Classification of Age-Related Macular Degeneration. JAMA ophthalmology. 2018;136(11):1305-1307.
16. Burlina P, Pacheco KD, Joshi N, Freund DE, Bressler NM. Comparing humans and deep learning performance for grading AMD: A study in using universal deep features and transfer learning for automated AMD analysis. Computers in Biology and Medicine. 2017;82:80-86.
17. Burlina PM, Joshi N, Pacheco KD, Freund DE, Kong J, Bressler NM. Use of deep learning for detailed severity characterization and estimation of 5-year risk among patients with age-related macular degeneration. JAMA ophthalmology. 2018;136(12):1359-1366.
18. Burlina PM, Joshi N, Pekala M, Pacheco KD, Freund DE, Bressler NM. Automated Grading of Age-Related Macular Degeneration From Color Fundus Images Using Deep Convolutional Neural Networks. JAMA ophthalmology. 2017;135(11):1170-1176.
19. Chan GCY, Kamble R, Muller H, Shah SAA, Tang TB, Meriaudeau F. Fusing Results of Several Deep Learning Architectures for Automatic Classification of Normal and Diabetic Macular Edema in Optical Coherence Tomography. Conference proceedings : Annual International Conference of the IEEE Engineering in Medicine and Biology Society IEEE Engineering in Medicine and Biology Society Annual Conference. 2018;2018:670-673.
20. Choi JY, Yoo TK, Seo JG, Kwak J, Um TT, Rim TH. Multi-categorical deep learning neural network to classify retinal images: A pilot study employing small database. PLOS ONE. 2017;12(11):e0187336.
21. Christopher M, Belghith A, Bowd C, et al. Performance of Deep Learning Architectures and Transfer Learning for Detecting Glaucomatous Optic Neuropathy in Fundus Photographs. Scientific Reports. 2018;8(1):16685.

22. Das V, Dandapat S, Bora PK. Multi-scale deep feature fusion for automated classification of macular pathologies from OCT images. Biomedical Signal Processing and Control. 2019;54:101605.
23. De Fauw J, Ledsam JR, Romera-Paredes B, et al. Clinically applicable deep learning for diagnosis and referral in retinal disease. Nature medicine. 2018;24(9):1342-1350.
24. ElTanboly A, Ismail M, Shalaby A, et al. A computer-aided diagnostic system for detecting diabetic retinopathy in optical coherence tomography images. Medical Physics. 2017;44(3):914-923.
25. Gargeya R, Leng T. Automated Identification of Diabetic Retinopathy Using Deep Learning. Ophthalmology. 2017;124(7):962-969.
26. Gómez-Valverde JJ, Antón A, Fatti G, et al. Automatic glaucoma classification using color fundus images based on convolutional neural networks and transfer learning. Biomedical Optics Express. 2019;10(2):892-913.
27. Grassmann F, Mengelkamp J, Brandl C, et al. A Deep Learning Algorithm for Prediction of Age-Related Eye Disease Study Severity Scale for Age-Related Macular Degeneration from Color Fundus Photography. Ophthalmology. 2018;125(9):1410-1420.
28. Gulshan V, Peng L, Coram M, et al. Development and validation of a deep learning algorithm for detection of diabetic retinopathy in retinal fundus photographs. Jama. 2016;316(22):2402-2410.
29. Gulshan V, Rajan RP, Widner K, et al. Performance of a Deep-Learning Algorithm vs Manual Grading for Detecting Diabetic Retinopathy in India. JAMA ophthalmology. 2019.
30. Hwang DK, Hsu CC, Chang KJ, et al. Artificial intelligence-based decision-making for age-related macular degeneration. Theranostics. 2019;9(1):232-245.
31. Jammal AA, Thompson AC, Mariottoni EB, et al. Human Versus Machine: Comparing a Deep Learning Algorithm to Human Gradings for Detecting Glaucoma on Fundus Photographs. American Journal of Ophthalmology. 2019.
32. Kanagasingam Y, Xiao D, Vignarajan J, Preetham A, Tay-Kearney M-L, Mehrotra A. Evaluation of Artificial Intelligence-Based Grading of Diabetic Retinopathy in Primary Care. JAMA network open. 2018;1(5):e182665-e182665.
33. Karri SP, Chakraborty D, Chatterjee J. Transfer learning based classification of optical coherence tomography images with diabetic macular edema and dry age-related macular degeneration. Biomed Opt Express. 2017;8(2):579-592.
34. Keel S, Lee PY, Scheetz J, et al. Feasibility and patient acceptability of a novel artificial intelligence-based screening model for diabetic retinopathy at endocrinology outpatient services: a pilot study. Scientific Reports. 2018;8(1):4330.
35. Keel S, Li Z, Scheetz J, et al. Development and validation of a deep-learning algorithm for the detection of neovascular age-related macular degeneration from colour fundus photographs. Clinical & Experimental Ophthalmology. 2019;47(8):1009-1018.
36. Keel S, Wu J, Lee PY, Scheetz J, He M. Visualizing Deep Learning Models for the Detection of Referable Diabetic Retinopathy and Glaucoma. JAMA ophthalmology. 2018.
37. Kermany DS, Goldbaum M, Cai W, et al. Identifying Medical Diagnoses and Treatable Diseases by Image-Based Deep Learning. Cell. 2018;172(5):1122-1131.e1129.
38. Krause J, Gulshan V, Rahimy E, et al. Grader Variability and the Importance of Reference Standards for Evaluating Machine Learning Models for Diabetic Retinopathy. Ophthalmology. 2018;125(8):1264-1272.
39. Lee CS, Baughman DM, Lee AY. Deep Learning Is Effective for Classifying Normal versus Age-Related Macular Degeneration OCT Images. Ophthalmology Retina. 2017;1(4):322-327.
40. Lee J, Kim Y, Kim JH, Park KH. Screening Glaucoma With Red-free Fundus Photography Using Deep Learning Classifier and Polar Transformation. J Glaucoma. 2019;28(3):258-264.
41. Li F, Chen H, Liu Z, Zhang X, Wu Z. Fully automated detection of retinal disorders by image-based deep learning. Graefe's Archive for Clinical and Experimental Ophthalmology. 2019;257(3):495-505.
42. Li F, Chen H, Liu Z, et al. Deep learning-based automated detection of retinal diseases using optical coherence tomography images. Biomed Opt Express. 2019;10(12):6204-6226.
43. Li F, Liu Z, Chen H, Jiang M, Zhang X, Wu Z. Automatic Detection of Diabetic Retinopathy in Retinal Fundus Photographs Based on Deep Learning Algorithm. Transl Vis Sci Technol. 2019;8(6):4.
44. Li F, Wang Z, Qu G, et al. Automatic differentiation of Glaucoma visual field from non-glaucoma visual filed using deep convolutional neural network. BMC medical imaging. 2018;18(1):35-35.
45. Li X, Shen L, Shen M, Tan F, Qiu CS. Deep learning based early stage diabetic retinopathy detection using optical coherence tomography. Neurocomputing. 2019;369:134-144.
46. Li Z, He Y, Keel S, Meng W, Chang RT, He M. Efficacy of a Deep Learning System for Detecting Glaucomatous Optic Neuropathy Based on Color Fundus Photographs. Ophthalmology. 2018;125(8):1199-1206.

47. Li Z, Keel S, Liu C, et al. An Automated Grading System for Detection of Vision-Threatening Referable Diabetic Retinopathy on the Basis of Color Fundus Photographs. Diabetes Care. 2018;41(12):2509-2516.
48. Lin H, Li R, Liu Z, et al. Diagnostic Efficacy and Therapeutic Decision-making Capacity of an Artificial Intelligence Platform for Childhood Cataracts in Eye Clinics: A Multicentre Randomized Controlled Trial. EclinicalMedicine. 2019;9:52-59.
49. Liu H, Li L, Wormstone IM, et al. Development and Validation of a Deep Learning System to Detect Glaucomatous Optic Neuropathy Using Fundus Photographs. JAMA ophthalmology. 2019;137(12):1353-1360.
50. Liu S, Graham SL, Schulz A, et al. A Deep Learning-Based Algorithm Identifies Glaucomatous Discs Using Monoscopic Fundus Photographs. Ophthalmology Glaucoma. 2018;1(1):15-22.
51. Long E, Lin H, Liu Z, et al. An artificial intelligence platform for the multihospital collaborative management of congenital cataracts. Nature Biomedical Engineering. 2017;1:0024.
52. MacCormick IJC, Williams BM, Zheng Y, et al. Accurate, fast, data efficient and interpretable glaucoma diagnosis with automated spatial analysis of the whole cup to disc profile. PLOS ONE. 2019;14(1):e0209409.
53. Maetschke S, Antony B, Ishikawa H, Wollstein G, Schuman J, Garnavi R. A feature agnostic approach for glaucoma detection in OCT volumes. PLOS ONE. 2019;14(7):e0219126.
54. Matsuba S, Tabuchi H, Ohsugi H, et al. Accuracy of ultra-wide-field fundus ophthalmoscopy-assisted deep learning, a machine-learning technology, for detecting age-related macular degeneration. International ophthalmology. 2018:1-7.
55. Medeiros FA, Jammal AA, Thompson AC. From Machine to Machine: An OCT-Trained Deep Learning Algorithm for Objective Quantification of Glaucomatous Damage in Fundus Photographs. Ophthalmology. 2019;126(4):513-521.
56. Motozawa N, An G, Takagi S, et al. Optical Coherence Tomography-Based Deep-Learning Models for Classifying Normal and Age-Related Macular Degeneration and Exudative and Non-Exudative Age-Related Macular Degeneration Changes. Ophthalmol Ther. 2019;8(4):527-539.
57. Muhammad H, Fuchs TJ, De Cuir N, et al. Hybrid Deep Learning on Single Wide-field Optical Coherence tomography Scans Accurately Classifies Glaucoma Suspects. J Glaucoma. 2017;26(12):1086-1094.
58. Nagasato D, Tabuchi H, Masumoto H, et al. Automated detection of a nonperfusion area caused by retinal vein occlusion in optical coherence tomography angiography images using deep learning. PloS One. 2019;14(11):e0223965.
59. Nagasato D, Tabuchi H, Ohsugi H, et al. Deep-learning classifier with ultrawide-field fundus ophthalmoscopy for detecting branch retinal vein occlusion. International journal of ophthalmology. 2019;12(1):94-99.
60. Nagasawa T, Tabuchi H, Masumoto H, et al. Accuracy of ultrawide-field fundus ophthalmoscopy-assisted deep learning for detecting treatment-I proliferative diabetic retinopathy. Int Ophthalmol. 2019.
61. Ohsugi H, Tabuchi H, Enno H, Ishitobi N. Accuracy of deep learning, a machine-learning technology, using ultra–wide-field fundus ophthalmoscopy for detecting rhegmatogenous retinal detachment. Scientific reports. 2017;7(1):9425.
62. Peng Y, Dharssi S, Chen Q, et al. DeepSeeNet: A Deep Learning Model for Automated Classification of Patient-based Age-related Macular Degeneration Severity from Color Fundus Photographs. Ophthalmology. 2019;126(4):565-575.
63. Perdomo O, Rios H, Rodríguez FJ, et al. Classification of diabetes-related retinal diseases using a deep learning approach in optical coherence tomography. Computer Methods and Programs in Biomedicine. 2019;178:181-189.
64. Phan S, Satoh Si, Yoda Y, et al. Evaluation of deep convolutional neural networks for glaucoma detection. Japanese Journal of Ophthalmology. 2019.
65. Phene S, Dunn RC, Hammel N, et al. Deep Learning and Glaucoma Specialists: The Relative Importance of Optic Disc Features to Predict Glaucoma Referral in Fundus Photographs. Ophthalmology. 2019;126(12):1627-1639.
66. Prahs P, Radeck V, Mayer C, et al. OCT-based deep learning algorithm for the evaluation of treatment indication with anti-vascular endothelial growth factor medications. Graefe's archive for clinical and experimental ophthalmology = Albrecht von Graefes Archiv fur klinische und experimentelle Ophthalmologie. 2018;256(1):91-98.
67. Raju M, Pagidimarri V, Barreto R, Kadam A, Kasivajjala V, Aswath A. Development of a Deep Learning Algorithm for Automatic Diagnosis of Diabetic Retinopathy. Paper presented at: MedInfo2017.
68. Ramachandran N, Hong SC, Sime MJ, Wilson GA. Diabetic retinopathy screening using deep neural network. Clin Exp Ophthalmol. 2018;46(4):412-416.

69. Raumviboonsuk P, Krause J, Chotcomwongse P, et al. Deep learning versus human graders for classifying diabetic retinopathy severity in a nationwide screening program. Npj Digital Medicine. 2019;2(1):25.
70. Redd TK, Campbell JP, Brown JM, et al. Evaluation of a deep learning image assessment system for detecting severe retinopathy of prematurity. British Journal of Ophthalmology. 2019;103(5):580-584.
71. Rogers TW, Jaccard N, Carbonaro F, et al. Evaluation of an AI system for the automated detection of glaucoma from stereoscopic optic disc photographs: the European Optic Disc Assessment Study. Eye. 2019;33(11):1791-1797.
72. Sandhu HS, Eltanboly A, Shalaby A, Keynton RS, Schaal S, El-Baz A. Automated Diagnosis and Grading of Diabetic Retinopathy Using Optical Coherence Tomography. Investigative ophthalmology & visual science. 2018;59(7):3155-3160.
73. Sayres R, Taly A, Rahimy E, et al. Using a Deep Learning Algorithm and Integrated Gradients Explanation to Assist Grading for Diabetic Retinopathy. Ophthalmology. 2019;126(4):552-564.
74. Shibata N, Tanito M, Mitsuhashi K, et al. Development of a deep residual learning algorithm to screen for glaucoma from fundus photography. Sci Rep. 2018;8(1):14665.
75. Stevenson CH, Hong SC, Ogbuehi KC. Development of an artificial intelligence system to classify pathology and clinical features on retinal fundus images. Clinical & Experimental Ophthalmology. 2019;47(4):484-489.
76. Ting DSW, Cheung CY, Lim G, et al. Development and Validation of a Deep Learning System for Diabetic Retinopathy and Related Eye Diseases Using Retinal Images From Multiethnic Populations With Diabetes. Jama. 2017;318(22):2211-2223.
77. Ting DSW, Cheung CY, Nguyen Q, et al. Deep learning in estimating prevalence and systemic risk factors for diabetic retinopathy: a multi-ethnic study. Npj Digital Medicine. 2019;2(1):24.
78. Treder M, Lauermann JL, Eter N. Automated detection of exudative age-related macular degeneration in spectral domain optical coherence tomography using deep learning. Graefe's Archive for Clinical and Experimental Ophthalmology. 2018;256(2):259-265.
79. Van Grinsven MJ, van Ginneken B, Hoyng CB, Theelen T, Sánchez CI. Fast convolutional neural network training using selective data sampling: Application to 8aemorrhage detection in color fundus images. IEEE transactions on medical imaging. 2016;35(5):1273-1284.
80. Verbraak FD, Abramoff MD, Bausch GCF, et al. Diagnostic Accuracy of a Device for the Automated Detection of Diabetic Retinopathy in a Primary Care Setting. Diabetes Care. 2019;42(4):651.
81. Xu K, Feng D, Mi H. Deep Convolutional Neural Network-Based Early Automated Detection of Diabetic Retinopathy Using Fundus Image. Molecules. 2017;22(12).
82. Yang W-H, Zheng B, Wu M-N, et al. An Evaluation System of Fundus Photograph-Based Intelligent Diagnostic Technology for Diabetic Retinopathy and Applicability for Research. Diabetes Ther. 2019;10(5):1811-1822.
83. Yoo TK, Choi JY, Seo JG, Ramasubramanian B, Selvaperumal S, Kim DW. The possibility of the combination of OCT and fundus images for improving the diagnostic accuracy of deep learning for age-related macular degeneration: a preliminary experiment. Medical & biological engineering & computing. 2019;57(3):677-687.
84. Zhang Y, Wang L, Wu Z, et al. Development of an Automated Screening System for Retinopathy of Prematurity Using a Deep Neural Network for Wide-Angle Retinal Images. IEEE Access. 2019;7:10232-10241.
85. Zheng C, Xie X, Huang L, et al. Detecting glaucoma based on spectral domain optical coherence tomography imaging of peripapillary retinal nerve fiber layer: a comparison study between hand-crafted features and deep learning model. Graefe's archive for clinical and experimental ophthalmology = Albrecht von Graefes Archiv fur klinische und experimentelle Ophthalmologie. 2019.

Supplementary References 2: Full list of included studies in respiratory imaging

1. Abiyev RH, Ma, #x, aitah MKS. Deep Convolutional Neural Networks for Chest Diseases Detection. Journal of Healthcare Engineering. 2018;2018:11.
2. Al-Shabi M, Lan BL, Chan WY, Ng KH, Tan M. Lung nodule classification using deep Local-Global networks. Int J Comput Assist Radiol Surg. 2019.
3. Alakwaa W, Nassef M, Badr A. Lung cancer detection and classification with 3D convolutional neural network (3D-CNN). Lung Cancer. 2017;8(8):409.
4. Ali I, Hart GR, Gunabushanam G, et al. Lung Nodule Detection via Deep Reinforcement Learning. Front Oncol. 2018;8:108.
5. Annarumma M, Withey SJ, Bakewell RJ, Pesce E, Goh V, Montana G. Automated Triaging of Adult Chest Radiographs with Deep Artificial Neural Networks. Radiology. 2019;291(1):196-202.
6. Ardila D, Kiraly AP, Bharadwaj S, et al. End-to-end lung cancer screening with three-dimensional deep learning on low-dose chest computed tomography. Nature Medicine. 2019.
7. Baltruschat IM, Nickisch H, Grass M, Knopp T, Saalbach A. Comparison of Deep Learning Approaches for Multi-Label Chest X-Ray Classification. Sci Rep. 2019;9(1):6381.
8. Bar Y, Diamant I, Wolf L, Lieberman S, Konen E, Greenspan H. Chest pathology identification using deep feature selection with non-medical training. Computer Methods in Biomechanics and Biomedical Engineering: Imaging & Visualization. 2018;6(3):259-263.

9. Becker AS, Blüthgen C, Phi van VD, et al. Detection of tuberculosis patterns in digital photographs of chest X-ray images using Deep Learning: feasibility study. Int J Tuberc Lung Dis. 2018;22(3):328-335.
10. Behzadi-khormouji H, Rostami H, Salehi S, et al. Deep learning, reusable and problem-based architectures for detection of consolidation on chest X-ray images. Computer Methods and Programs in Biomedicine. 2020;185:105162.
11. Beig N, Khorrami M, Alilou M, et al. Perinodular and Intranodular Radiomic Features on Lung CT Images Distinguish Adenocarcinomas from Granulomas. Radiology. 2019;290(3):783-792.
12. Causey JL, Zhang J, Ma S, et al. Highly accurate model for prediction of lung nodule malignancy with CT scans. Scientific reports. 2018;8(1):9286.
13. Cha MJ, Chung MJ, Lee JH, Lee KS. Performance of Deep Learning Model in Detecting Operable Lung Cancer With Chest Radiographs. J Thorac Imaging. 2019;34(2):86-91.
14. Chae KJ, Jin GY, Ko SB, et al. Deep Learning for the Classification of Small (≤2 cm) Pulmonary Nodules on CT Imaging: A Preliminary Study. Academic Radiology.
15. Chen G, Zhang J, Zhuo D, Pan Y, Pang C. Identification of pulmonary nodules via CT images with hierarchical fully convolutional networks. Medical & biological engineering & computing. 2019;57(7):1567-1580.
16. Cheng J-Z, Ni D, Chou Y-H, et al. Computer-Aided Diagnosis with Deep Learning Architecture: Applications to Breast Lesions in US Images and Pulmonary Nodules in CT Scans. 2016;6:24454.
17. Cicero M, Bilbily A, Colak E, et al. Training and Validating a Deep Convolutional Neural Network for Computer-Aided Detection and Classification of Abnormalities on Frontal Chest Radiographs. Invest Radiol. 2017;52(5):281-287.
18. Ciompi F, Chung K, van Riel SJ, et al. Towards automatic pulmonary nodule management in lung cancer screening with deep learning. Scientific Reports. 2017;7:46479.
19. Correa M, Zimic M, Barrientos F, et al. Automatic classification of pediatric pneumonia based on lung ultrasound pattern recognition. PLOS ONE. 2018;13(12):e0206410.
20. da Silva GL, da Silva Neto OP, Silva AC, de Paiva AC, Gattass M. Lung nodules diagnosis based on evolutionary convolutional neural network. Multimedia Tools and Applications. 2017;76(18):19039-19055.
21. da Silva GLF, Valente TLA, Silva AC, de Paiva AC, Gattass M. Convolutional neural network-based PSO for lung nodule false positive reduction on CT images. Comput Methods Programs Biomed. 2018;162:109-118.
22. Dai Y, Yan S, Zheng B, Song C. Incorporating automatically learned pulmonary nodule attributes into a convolutional neural network to improve accuracy of benign-malignant nodule classification. Phys Med Biol. 2018;63(24):245004.
23. Dou Q, Chen H, Yu L, Qin J, Heng PA. Multilevel Contextual 3-D CNNs for False Positive Reduction in Pulmonary Nodule Detection. IEEE Transactions on Biomedical Engineering. 2017;64(7):1558-1567.
24. Dunnmon JA, Yi D, Langlotz CP, Ré C, Rubin DL, Lungren MP. Assessment of Convolutional Neural Networks for Automated Classification of Chest Radiographs. Radiology. 2018;290(2):537-544.
25. Gao M, Bagci U, Lu L, et al. Holistic classification of CT attenuation patterns for interstitial lung diseases via deep convolutional neural networks. Comput Methods Biomech Biomed Eng Imaging Vis. 2018;6(1):1-6.

26. Gong L, Jiang S, Yang Z, Zhang G, Wang L. Automated pulmonary nodule detection in CT images using 3D deep squeeze-and-excitation networks. Int J Comput Assist Radiol Surg. 2019.
27. Gonzalez Y, Shen C, Li B, Klages P, Jia X. Dose reduction in X-ray computed tomography via a deep-learning approach. Medical Physics. 2017;44 (6):3011.
28. Gruetzemacher R, Gupta A, Paradice D. 3D deep learning for detecting pulmonary nodules in CT scans. J Am Med Inform Assoc. 2018;25(10):1301-1310.
29. Gu Y, Lu X, Yang L, et al. Automatic lung nodule detection using a 3D deep convolutional neural network combined with a multi-scale prediction strategy in chest CTs. Comput Biol Med. 2018;103:220-231.
30. Hamidian S, Sahiner B, Petrick N, Pezeshk A. 3D Convolutional Neural Network for Automatic Detection of Lung Nodules in Chest CT. Proc SPIE Int Soc Opt Eng. 2017;10134.
31. Han G, Liu X, Zheng G, Wang M, Huang S. Automatic recognition of 3D GGO CT imaging signs through the fusion of hybrid resampling and layer-wise fine-tuning CNNs. Medical & biological engineering & computing. 2018;56(12):2201-2212.
32. Heo SJ, Kim Y, Yun S, et al. Deep Learning Algorithms with Demographic Information Help to Detect Tuberculosis in Chest Radiographs in Annual Workers' Health Examination Data. Int J Environ Res Public Health. 2019;16(2).
33. Hua K-L, Hsu C-H, Hidayati SC, Cheng W-H, Chen Y-J. Computer-aided classification of lung nodules on computed tomography images via deep learning technique. OncoTargets and therapy. 2015;8:2015-2022.
34. Huang W, Xue Y, Wu Y. A CAD system for pulmonary nodule prediction based on deep three-dimensional convolutional neural networks and ensemble learning. PloS One. 2019;14(7):e0219369.
35. Huang X, Sun W, Tseng T-L, Li C, Qian W. Fast and fully-automated detection and segmentation of pulmonary nodules in thoracic CT scans using deep convolutional neural networks. Computerized Medical Imaging and Graphics. 2019;74:25-36.
36. Hussein S, Kandel P, Bolan CW, Wallace MB, Bagci U. Lung and Pancreatic Tumor Characterization in the Deep Learning Era: Novel Supervised and Unsupervised Learning Approaches. IEEE Transactions on Medical Imaging. 2019;38(8):1777-1787.
37. Hwang EJ, Nam JG, Lim WH, et al. Deep Learning for Chest Radiograph Diagnosis in the Emergency Department. Radiology. 2019;293(3):573-580.
38. Hwang EJ, Park S, Jin K-N, et al. Development and Validation of a Deep Learning–Based Automated Detection Algorithm for Major Thoracic Diseases on Chest Radiographs. JAMA Network Open. 2019;2(3):e191095-e191095.
39. Hwang EJ, Park S, Jin K-N, et al. Development and Validation of a Deep Learning–based Automatic Detection Algorithm for Active Pulmonary Tuberculosis on Chest Radiographs. Clinical Infectious Diseases. 2018.
40. Jiang H, Ma H, Qian W, Gao M, Li Y. An Automatic Detection System of Lung Nodule Based on Multi-Group Patch-Based Deep Learning Network. IEEE Journal of Biomedical and Health Informatics. 2017;14.
41. Jin H, Li Z, Tong R, Lin L. A deep 3D residual CNN for false-positive reduction in pulmonary nodule detection. Med Phys. 2018;45(5):2097-2107.
42. Jung H, Kim B, Lee I, Lee J, Kang J. Classification of lung nodules in CT scans using three-dimensional deep convolutional neural networks with a checkpoint ensemble method. BMC Med Imaging. 2018;18(1):48.
43. Kang G, Liu K, Hou B, Zhang N. 3D multi-view convolutional neural networks for lung nodule classification. PloS one. 2017;12(11):e0188290.
44. Kermany DS, Goldbaum M, Cai W, et al. Identifying Medical Diagnoses and Treatable Diseases by Image-Based Deep Learning. Cell. 2018;172(5):1122-1131.e1129.
45. Kim B-C, Yoon JS, Choi J-S, Suk H-I. Multi-scale gradual integration CNN for false positive reduction in pulmonary nodule detection. Neural Networks. 2019;115:1-10.
46. Lakhani P, Sundaram B. Deep learning at chest radiography: Automated classification of pulmonary tuberculosis by using convolutional neural networks. Radiology. 2017;284(2):574-582.
47. Li L, Liu Z, Huang H, Lin M, Luo D. Evaluating the performance of a deep learning-based computer-aided diagnosis (DL-CAD) system for detecting and characterizing lung nodules: Comparison with the performance of double reading by radiologists. Thorac Cancer. 2019;10(2):183-192.
48. Li W, Cao P, Zhao D, Wang J. Pulmonary Nodule Classification with Deep Convolutional Neural Networks on Computed Tomography Images. Comput Math Methods Med. 2016;2016:6215085-6215085.
49. Li X, Thrall JH, Digumarthy SR, et al. Deep learning-enabled system for rapid pneumothorax screening on chest CT. European Journal of Radiology. 2019;120:108692.

50. Liang CH, Liu YC, Wu MT, Garcia-Castro F, Alberich-Bayarri A, Wu FZ. Identifying pulmonary nodules or masses on chest radiography using deep learning: external validation and strategies to improve clinical practice. Clinical radiology. 2020;75(1):38-45.
51. Liang G, Zheng L. A transfer learning method with deep residual network for pediatric pneumonia diagnosis. Comput Methods Programs Biomed. 2019.
52. Liu H, Cao H, Song E, et al. A cascaded dual-pathway residual network for lung nodule segmentation in CT images. Phys Med. 2019;63:112-121.
53. Liu H, Wang L, Nan Y, Jin F, Wang Q, Pu J. SDFN: Segmentation-based deep fusion network for thoracic disease classification in chest X-ray images. Comput Med Imaging Graph. 2019;75:66-73.
54. Liu S, Xie Y, Jirapatnakul A, Reeves AP. Pulmonary nodule classification in lung cancer screening with three-dimensional convolutional neural networks. Journal of Medical Imaging. 2017;4(4):041308.
55. Majkowska A, Mittal S, Steiner DF, et al. Chest Radiograph Interpretation with Deep Learning Models: Assessment with Radiologist-adjudicated Reference Standards and Population-adjusted Evaluation. Radiology. 2019:191293.
56. Monkam P, Qi S, Xu M, Han F, Zhao X, Qian W. CNN models discriminating between pulmonary micro-nodules and non-nodules from CT images. BioMedical Engineering OnLine. 2018;17(1):96.
57. Nam JG, Park S, Hwang EJ, et al. Development and Validation of Deep Learning–based Automatic Detection Algorithm for Malignant Pulmonary Nodules on Chest Radiographs. Radiology. 2018;290(1):218-228.
58. Naqi SM, Sharif M, Jaffar A. Lung nodule detection and classification based on geometric fit in parametric form and deep learning. Neural Computing and Applications. 2020;32(9):4629-4647.
59. Nasrullah N, Sang J, Alam MS, Mateen M, Cai B, Hu H. Automated Lung Nodule Detection and Classification Using Deep Learning Combined with Multiple Strategies. Sensors (Basel, Switzerland). 2019;19(17):3722.
60. Nibali A, He Z, Wollersheim D. Pulmonary nodule classification with deep residual networks. Int J Comput Assist Radiol Surg. 2017;12(10):1799-1808.
61. Nishio M, Sugiyama O, Yakami M, et al. Computer-aided diagnosis of lung nodule classification between benign nodule, primary lung cancer, and metastatic lung cancer at different image size using deep convolutional neural network with transfer learning. PloS One. 2018;13(7):e0200721.
62. Onishi Y, Teramoto A, Tsujimoto M, et al. Automated Pulmonary Nodule Classification in Computed Tomography Images Using a Deep Convolutional Neural Network Trained by Generative Adversarial Networks. BioMed research international. 2019;2019:6051939-6051939.
63. Onishi Y, Teramoto A, Tsujimoto M, et al. Multiplanar analysis for pulmonary nodule classification in CT images using deep convolutional neural network and generative adversarial networks. International Journal of Computer Assisted Radiology and Surgery. 2019.
64. Park S, Lee SM, Kim N, et al. Application of deep learning-based computer-aided detection system: detecting pneumothorax on chest radiograph after biopsy. Eur Radiol. 2019;29(10):5341-5348.
65. Park S, Lee SM, Lee KH, et al. Deep learning-based detection system for multiclass lesions on chest radiographs: comparison with observer readings. European Radiology. 2019.
66. Pasa F, Golkov V, Pfeiffer F, Cremers D, Pfeiffer D. Efficient Deep Network Architectures for Fast Chest X-Ray Tuberculosis Screening and Visualization. Scientific Reports. 2019;9(1):6268.
67. Patel BN, Rosenberg L, Willcox G, et al. Human–machine partnership with artificial intelligence for chest radiograph diagnosis. Npj Digital Medicine. 2019;2(1):111.
68. Paul R, Hall L, Goldgof D, Schabath M, Gillies R. Predicting Nodule Malignancy using a CNN Ensemble Approach. Proc Int Jt Conf Neural Netw. 2018;2018.
69. Pesce E, Joseph Withey S, Ypsilantis PP, Bakewell R, Goh V, Montana G. Learning to detect chest radiographs containing pulmonary lesions using visual attention networks. Med Image Anal. 2019;53:26-38.
70. Pezeshk A, Hamidian S, Petrick N, Sahiner B. 3-D Convolutional Neural Networks for Automatic Detection of Pulmonary Nodules in Chest CT. IEEE Journal of Biomedical and Health Informatics. 2019;23(5):2080-2090.
71. Qin ZZ, Sander MS, Rai B, et al. Using artificial intelligence to read chest radiographs for tuberculosis detection: A multi-site evaluation of the diagnostic accuracy of three deep learning systems. Sci Rep. 2019;9(1):15000.
72. Rajpurkar P, Irvin J, Ball RL, et al. Deep learning for chest radiograph diagnosis: A retrospective comparison of the CheXNeXt algorithm to practicing radiologists. PLOS Medicine. 2018;15(11):e1002686.
73. Ren Y, Tsai M-Y, Chen L, et al. A manifold learning regularization approach to enhance 3D CT image-based lung nodule classification. International Journal of Computer Assisted Radiology and Surgery. 2019.

74. Sahu P, Yu D, Dasari M, Hou F, Qin H. A Lightweight Multi-Section CNN for Lung Nodule Classification and Malignancy Estimation. IEEE Journal of Biomedical and Health Informatics. 2019;23(3):960-968.
75. Schwyzer M, Ferraro DA, Muehlematter UJ, et al. Automated detection of lung cancer at ultralow dose PET/CT by deep neural networks – Initial results. Lung Cancer. 2018;126:170-173.
76. Setio AAA, Ciompi F, Litjens G, et al. Pulmonary Nodule Detection in CT Images: False Positive Reduction Using Multi-View Convolutional Networks. IEEE Transactions on Medical Imaging. 2016;35(5):1160-1169.
77. Shaffie A, Soliman A, Fraiwan L, et al. A Generalized Deep Learning-Based Diagnostic System for Early Diagnosis of Various Types of Pulmonary Nodules. Technol Cancer Res Treat. 2018;17:1533033818798800.
78. Shen W, Zhou M, Yang F, et al. Multi-crop Convolutional Neural Networks for lung nodule malignancy suspiciousness classification. Pattern Recognition. 2017;61:663-673.
79. Sim Y, Chung MJ, Kotter E, et al. Deep Convolutional Neural Network–based Software Improves Radiologist Detection of Malignant Lung Nodules on Chest Radiographs. Radiology. 2020;294(1):199-209.
80. Singh R, Kalra MK, Nitiwarangkul C, et al. Deep learning in chest radiography: Detection of findings and presence of change. PloS One. 2018;13(10):e0204155.
81. Song Q, Zhao L, Luo X, Dou X. Using Deep Learning for Classification of Lung Nodules on Computed Tomography Images. J Healthc Eng. 2017;2017:8314740.
82. Stephen O, Sain M, Maduh UJ, Jeong D-U. An Efficient Deep Learning Approach to Pneumonia Classification in Healthcare. Journal of Healthcare Engineering. 2019;2019:7.
83. Sun W, Zheng B, Qian W. Automatic feature learning using multichannel ROI based on deep structured algorithms for computerized lung cancer diagnosis. Computers in biology and medicine. 2017;89:530-539.
84. Tan J, Huo Y, Liang Z, Li L. Expert knowledge-infused deep learning for automatic lung nodule detection. J Xray Sci Technol. 2019;27(1):17-35.
85. Taylor AG, Mielke C, Mongan J. Automated detection of moderate and large pneumothorax on frontal chest X-rays using deep convolutional neural networks: A retrospective study. PloS medicine. 2018;15(11):e1002697-e1002697.
86. Teramoto A, Fujita H, Yamamuro O, Tamaki T. Automated detection of pulmonary nodules in PET/CT images: Ensemble false-positive reduction using a convolutional neural network technique. Medical Physics. 2016;43(6Part1):2821-2827.
87. Toğaçar M, Ergen B, Cömert Z. A Deep Feature Learning Model for Pneumonia Detection Applying a Combination of mRMR Feature Selection and Machine Learning Models. IRBM. 2019.
88. Toğaçar M, Ergen B, Cömert Z. Detection of lung cancer on chest CT images using minimum redundancy maximum relevance feature selection method with convolutional neural networks. Biocybernetics and Biomedical Engineering. 2020;40(1):23-39.
89. Tran GS, Nghiem TP, Nguyen VT, Luong CM, Burie J-C. Improving Accuracy of Lung Nodule Classification Using Deep Learning with Focal Loss. Journal of healthcare engineering. 2019;2019:5156416-5156416.
90. Tu X, Xie M, Gao J, et al. Automatic categorization and scoring of solid, part-solid and non-solid pulmonary nodules in CT images with convolutional neural network. Scientific reports. 2017;7(1):8533.
91. Uthoff J, Stephens MJ, Newell Jr JD, et al. Machine learning approach for distinguishing malignant and benign lung nodules utilizing standardized perinodular parenchymal features from CT. Medical Physics.0(0).
92. Walsh SLF, Calandriello L, Silva M, Sverzellati N. Deep learning for classifying fibrotic lung disease on high-resolution computed tomography: a case-cohort study. The Lancet Respiratory Medicine. 2018;6(11):837-845.
93. Wang C, Elazab A, Jia F, Wu J, Hu Q. Automated chest screening based on a hybrid model of transfer learning and convolutional sparse denoising autoencoder. Biomedical engineering online. 2018;17(1):63-63.
94. Wang C, Elazab A, Wu J, Hu Q. Lung nodule classification using deep feature fusion in chest radiography. Comput Med Imaging Graph. 2017;57:10-18.
95. Wang H, Jia H, Lu L, Xia Y. Thorax-Net: An Attention Regularized Deep Neural Network for Classification of Thoracic Diseases on Chest Radiography. IEEE J Biomed Health Inform. 2019.
96. Wang Q, Shen F, Shen L, Huang J, Sheng W. Lung Nodule Detection in CT Images Using a Raw Patch-Based Convolutional Neural Network. Journal of digital imaging. 2019.
97. Wang S, Wang R, Zhang S, et al. 3D convolutional neural network for differentiating pre-invasive lesions from invasive adenocarcinomas appearing as ground-glass nodules with diameters </=3 cm using HRCT. Quant Imaging Med Surg. 2018;8(5):491-499.

98. Wang Y, Sun LL, Jin Q. Enhanced Diagnosis of Pneumothorax with an Improved Real-time Augmentation for Imbalanced Chest X-rays Data Based on DCNN. IEEE/ACM Transactions on Computational Biology and Bioinformatics. 2019:1-1.
99. Xie Y, Xia Y, Zhang J, et al. Knowledge-based Collaborative Deep Learning for Benign-Malignant Lung Nodule Classification on Chest CT. IEEE Trans Med Imaging. 2019;38(4):991-1004.
100. Xie Y, Zhang J, Xia Y, Fulham M, Zhang Y. Fusing texture, shape and deep model-learned information at decision level for automated classification of lung nodules on chest CT. Information Fusion. 2018;42:102-110.
101. Yates EJ, Yates LC, Harvey H. Machine learning "red dot": open-source, cloud, deep convolutional neural networks in chest radiograph binary normality classification. Clinical radiology. 2018;73(9):827-831.
102. Ye W, Gu W, Guo X, et al. Detection of pulmonary ground-glass opacity based on deep learning computer artificial intelligence. Biomed Eng Online. 2019;18(1):6.
103. Zech JR, Badgeley MA, Liu M, Costa AB, Titano JJ, Oermann EK. Variable generalization performance of a deep learning model to detect pneumonia in chest radiographs: A cross-sectional study. PLOS Medicine. 2018;15(11):e1002683.
104. Zhang C, Sun X, Dang K, et al. Toward an Expert Level of Lung Cancer Detection and Classification Using a Deep Convolutional Neural Network. Oncologist. 2019;24(9):1159-1165.
105. Zhang G, Yang Z, Gong L, Jiang S, Wang L. Classification of benign and malignant lung nodules from CT images based on hybrid features. Physics in Medicine & Biology. 2019;64(12):125011.
106. Zhang J, Xia Y, Zeng H, Zhang Y. NODULe: Combining constrained multi-scale LoG filters with densely dilated 3D deep convolutional neural network for pulmonary nodule detection. Neurocomputing. 2018;317:159-167.
107. Zhang R, Cheng C, Zhao X, Li X. Multiscale Mask R-CNN-Based Lung Tumor Detection Using PET Imaging. Mol Imaging. 2019;18:1536012119863531-1536012119863531.
108. Zhang S, Han F, Liang Z, et al. An investigation of CNN models for differentiating malignant from benign lesions using small pathologically proven datasets. Computerized Medical Imaging and Graphics. 2019;77:101645.
109. Zhang S, Sun F, Wang N, et al. Computer-Aided Diagnosis (CAD) of Pulmonary Nodule of Thoracic CT Image Using Transfer Learning. Journal of digital imaging. 2019;32(6):995-1007.
110. Zhang T, Zhao J, Luo J, Qiang Y. Deep Belief Network for Lung Nodules Diagnosed in CT Imaging. International Journal of Performability Engineering. 2017;13(8).
111. Zhao X, Liu L, Qi S, Teng Y, Li J, Qian W. Agile convolutional neural network for pulmonary nodule classification using CT images. Int J Comput Assist Radiol Surg. 2018;13(4):585-595.
112. Zhao X, Qi S, Zhang B, et al. Deep CNN models for pulmonary nodule classification: model modification, model integration, and transfer learning. J Xray Sci Technol. 2019.
113. Zheng S, Guo J, Cui X, Veldhuis RNJ, Oudkerk M, van Ooijen PMA. Automatic Pulmonary Nodule Detection in CT Scans Using Convolutional Neural Networks Based on Maximum Intensity Projection. IEEE Trans Med Imaging. 2019.
114. Zhou S, Zhang X, Zhang R. Identifying Cardiomegaly in ChestX-ray8 Using Transfer Learning. Stud Health Technol Inform. 2019;264:482-486.

Supplementary References 3: Full list of included studies in breast imaging

1. Abdelsamea MM, Mohamed MH, Bamatraf M. Automated Classification of Malignant and Benign Breast Cancer Lesions Using Neural Networks on Digitized Mammograms. Cancer Inform. 2019;18:1176935119857570-1176935119857570.
2. Agnes SA, Anitha J, Pandian SIA, Peter JD. Classification of Mammogram Images Using Multiscale all Convolutional Neural Network (MA-CNN). Journal of Medical Systems. 2019;44(1):30.
3. Akselrod-Ballin A, Karlinsky L, Alpert S, Hashoul S, Ben-Ari R, Barkan E. A CNN based method for automatic mass detection and classification in mammograms. Computer Methods in Biomechanics and Biomedical Engineering: Imaging & Visualization. 2019;7(3):242-249.
4. Al-Antari MA, Al-Masni MA, Choi MT, Han SM, Kim TS. A fully integrated computer-aided diagnosis system for digital X-ray mammograms via deep learning detection, segmentation, and classification. Int J Med Inform. 2018;117:44-54.
5. Al-antari MA, Al-masni MA, Park S-U, et al. An Automatic Computer-Aided Diagnosis System for Breast Cancer in Digital Mammograms via Deep Belief Network. Journal of Medical and Biological Engineering. 2018;38(3):443-456.
6. Al-masni MA, Al-antari MA, Park J-M, et al. Simultaneous detection and classification of breast masses in digital mammograms via a deep learning YOLO-based CAD system. Computer Methods and Programs in Biomedicine. 2018;157:85-94.
7. Antropova N, Abe H, Giger ML. Use of clinical MRI maximum intensity projections for improved breast lesion classification with deep convolutional neural networks. Journal of medical imaging (Bellingham, Wash). 2018;5(1):014503-014503.
8. Antropova N, Huynh B, Li H, Giger ML. Breast lesion classification based on dynamic contrast-enhanced magnetic resonance images sequences with long short-term memory networks. J Med Imaging (Bellingham). 2019;6(1):011002.
9. Antropova N, Huynh BQ, Giger ML. A deep feature fusion methodology for breast cancer diagnosis demonstrated on three imaging modality datasets. Medical Physics. 2017;44(10):5162-5171.
10. Arevalo J, González FA, Ramos-Pollán R, Oliveira JL, Guevara Lopez MA. Representation learning for mammography mass lesion classification with convolutional neural networks. Computer Methods and Programs in Biomedicine. 2016;127:248-257.
11. Bandeira Diniz JO, Bandeira Diniz PH, Azevedo Valente TL, Corrêa Silva A, de Paiva AC, Gattass M. Detection of mass regions in mammograms by bilateral analysis adapted to breast density using similarity indexes and convolutional neural networks. Computer Methods and Programs in Biomedicine. 2018;156:191-207.
12. Becker AS, Marcon M, Ghafoor S, Wurnig MC, Frauenfelder T, Boss A. Deep Learning in Mammography: Diagnostic Accuracy of a Multipurpose Image Analysis Software in the Detection of Breast Cancer. Invest Radiol. 2017;52(7):434-440.
13. Becker AS, Mueller M, Stoffel E, Marcon M, Ghafoor S, Boss A. Classification of breast cancer in ultrasound imaging using a generic deep learning analysis software: a pilot study. Br J Radiol. 2018;91(1083):20170576.
14. Bevilacqua V, Brunetti A, Guerriero A, Trotta GF, Telegrafo M, Moschetta M. A performance comparison between shallow and deeper neural networks supervised classification of tomosynthesis breast lesions images. Cognitive Systems Research. 2019;53:3-19.
15. Byra M, Galperin M, Ojeda-Fournier H, et al. Breast mass classification in sonography with transfer learning using a deep convolutional neural network and color conversion. Med Phys. 2019;46(2):746-755.
16. Cai H, Huang Q, Rong W, et al. Breast Microcalcification Diagnosis Using Deep Convolutional Neural Network from Digital Mammograms. Comput Math Methods Med. 2019;2019:2717454.
17. Cao Z, Duan L, Yang G, Yue T, Chen Q. An experimental study on breast lesion detection and classification from ultrasound images using deep learning architectures. BMC Medical Imaging. 2019;19(1):51.
18. Cao Z, Yang G, Chen Q, Chen X, Lv F. Breast tumor classification through learning from noisy labeled ultrasound images. Medical Physics. 2019;n/a(n/a).
19. Cheng J-Z, Ni D, Chou Y-H, et al. Computer-Aided Diagnosis with Deep Learning Architecture: Applications to Breast Lesions in US Images and Pulmonary Nodules in CT Scans. 2016;6:24454.
20. Chiao J-Y, Chen K-Y, Liao KY-K, Hsieh P-H, Zhang G, Huang T-C. Detection and classification the breast tumors using mask R-CNN on sonograms. Medicine. 2019;98(19):e15200.
21. Choi JS, Han B-K, Ko ES, et al. Effect of a Deep Learning Framework-Based Computer-Aided Diagnosis System on the Diagnostic Performance of Radiologists in Differentiating between Malignant and Benign Masses on Breast Ultrasonography. Korean J Radiol. 2019;20(5):749-758.
22. Chougrad H, Zouaki H, Alheyane O. Deep Convolutional Neural Networks for breast cancer screening. Computer Methods and Programs in Biomedicine. 2018;157:19-30.

23. Ciritsis A, Rossi C, Eberhard M, Marcon M, Becker AS, Boss A. Automatic classification of ultrasound breast lesions using a deep convolutional neural network mimicking human decision-making. Eur Radiol. 2019;29(10):5458-5468.
24. Cogan T, Cogan M, Tamil L. RAMS: Remote and automatic mammogram screening. Comput Biol Med. 2019;107:18-29.
25. Dalmis MU, Gubern-Mérida A, Vreemann S, et al. Artificial Intelligence–Based Classification of Breast Lesions Imaged With a Multiparametric Breast MRI Protocol With Ultrafast DCE-MRI, T2, and DWI. Investigative Radiology. 2019;54(6):325-332.
26. Dalmış MU, Vreemann S, Kooi T, Mann RM, Karssemeijer N, Gubern-Mérida A. Fully automated detection of breast cancer in screening MRI using convolutional neural networks. Journal of medical imaging (Bellingham, Wash). 2018;5(1):014502-014502.
27. Dhungel N, Carneiro G, Bradley AP. A deep learning approach for the analysis of masses in mammograms with minimal user intervention. Med Image Anal. 2017;37:114-128.
28. Duggento A, Aiello M, Cavaliere C, et al. An Ad Hoc Random Initialization Deep Neural Network Architecture for Discriminating Malignant Breast Cancer Lesions in Mammographic Images. Contrast Media & Molecular Imaging. 2019;2019:9.
29. Fan M, Li Y, Zheng S, Peng W, Tang W, Li L. Computer-aided detection of mass in digital breast tomosynthesis using a faster region-based convolutional neural network. Methods. 2019;166:103-111.
30. Fujioka T, Kubota K, Mori M, et al. Distinction between benign and malignant breast masses at breast ultrasound using deep learning method with convolutional neural network. Jpn J Radiol. 2019;37(6):466-472.
31. Gao F, Wu T, Li J, et al. SD-CNN: A shallow-deep CNN for improved breast cancer diagnosis. Computerized Medical Imaging and Graphics. 2018;70:53-62.
32. Ha R, Mutasa S, Sant EPV, et al. Accuracy of Distinguishing Atypical Ductal Hyperplasia From Ductal Carcinoma In Situ With Convolutional Neural Network–Based Machine Learning Approach Using Mammographic Image Data. American Journal of Roentgenology. 2019;212(5):1166-1171.
33. Han S, Kang HK, Jeong JY, et al. A deep learning framework for supporting the classification of breast lesions in ultrasound images. Phys Med Biol. 2017;62(19):7714-7728.
34. Herent P, Schmauch B, Jehanno P, et al. Detection and characterization of MRI breast lesions using deep learning. Diagn Interv Imaging. 2019;100(4):219-225.
35. Hizukuri A, Nakayama R. Computer-Aided Diagnosis Scheme for Determining Histological Classification of Breast Lesions on Ultrasonographic Images Using Convolutional Neural Network. Diagnostics (Basel). 2018;8(3):48.
36. Huynh BQ, Li H, Giger ML. Digital mammographic tumor classification using transfer learning from deep convolutional neural networks. J Med Imaging (Bellingham). 2016;3(3):034501.
37. Jadoon MM, Zhang Q, Haq IU, Butt S, Jadoon A. Three-Class Mammogram Classification Based on Descriptive CNN Features. BioMed Research International. 2017;2017:11.
38. Jiao Z, Gao X, Wang Y, Li J. A deep feature based framework for breast masses classification. Neurocomputing. 2016;197:221-231.
39. Jiao Z, Gao X, Wang Y, Li J. A parasitic metric learning net for breast mass classification based on mammography. Pattern Recognition. 2018;75:292-301.
40. Jung H, Kim B, Lee I, et al. Detection of masses in mammograms using a one-stage object detector based on a deep convolutional neural network. PLOS ONE. 2018;13(9):e0203355.
41. Kim E-K, Kim H-E, Han K, et al. Applying Data-driven Imaging Biomarker in Mammography for Breast Cancer Screening: Preliminary Study. Scientific Reports. 2018;8(1):2762.
42. Kim SM, Han H, Park JM, et al. A Comparison of Logistic Regression Analysis and an Artificial Neural Network Using the BI-RADS Lexicon for Ultrasonography in Conjunction with Introbserver Variability. Journal of digital imaging. 2012;25(5):599-606.
43. Kim ST, Lee JH, Lee H, Ro YM. Visually interpretable deep network for diagnosis of breast masses on mammograms. Phys Med Biol. 2018;63(23):235025.
44. Kooi T, Karssemeijer N. Classifying symmetrical differences and temporal change for the detection of malignant masses in mammography using deep neural networks. Journal of Medical Imaging. 2017;4(4):1-9, 9.
45. Kooi T, van Ginneken B, Karssemeijer N, den Heeten A. Discriminating solitary cysts from soft tissue lesions in mammography using a pretrained deep convolutional neural network. Med Phys. 2017;44(3):1017-1027.
46. Kooi T, Litjens G, van Ginneken B, et al. Large scale deep learning for computer aided detection of mammographic lesions. Med Image Anal. 2017;35:303-312.
47. Li H, Zhuang S, Li D-a, Zhao J, Ma Y. Benign and malignant classification of mammogram images based on deep learning. Biomedical Signal Processing and Control. 2019;51:347-354.
48. Li X, Qin G, He Q, et al. Digital breast tomosynthesis versus digital mammography: integration of image modalities enhances deep learning-based breast mass classification. European Radiology. 2019.

49. Lin C, Hou Y, Chen T, Chen K. Breast Nodules Computer-Aided Diagnostic System Design Using Fuzzy Cerebellar Model Neural Networks. IEEE Transactions on Fuzzy Systems. 2014;22(3):693-699.
50. McKinney SM, Sieniek M, Godbole V, et al. International evaluation of an AI system for breast cancer screening. Nature. 2020;577(7788):89-94.
51. Mendel K, Li H, Sheth D, Giger M. Transfer Learning From Convolutional Neural Networks for Computer-Aided Diagnosis: A Comparison of Digital Breast Tomosynthesis and Full-Field Digital Mammography. Acad Radiol. 2019;26(6):735-743.
52. Peng W, Mayorga RV, Hussein EMA. An automated confirmatory system for analysis of mammograms. Computer Methods and Programs in Biomedicine. 2016;125:134-144.
53. Qi X, Zhang L, Chen Y, et al. Automated diagnosis of breast ultrasonography images using deep neural networks. Medical Image Analysis. 2019;52:185-198.
54. Qiu Y, Yan S, Gundreddy RR, et al. A new approach to develop computer-aided diagnosis scheme of breast mass classification using deep learning technology. J Xray Sci Technol. 2017;25(5):751-763.
55. Ragab DA, Sharkas M, Marshall S, Ren J. Breast cancer detection using deep convolutional neural networks and support vector machines. PeerJ. 2019;7:e6201-e6201.
56. Ribli D, Horváth A, Unger Z, Pollner P, Csabai I. Detecting and classifying lesions in mammograms with Deep Learning. Scientific Reports. 2018;8(1):4165.
57. Rodríguez-Ruiz A, Krupinski E, Mordang J-J, et al. Detection of Breast Cancer with Mammography: Effect of an Artificial Intelligence Support System. Radiology. 2018;290(2):305-314.
58. Rodriguez-Ruiz A, Lång K, Gubern-Merida A, et al. Stand-Alone Artificial Intelligence for Breast Cancer Detection in Mammography: Comparison With 101 Radiologists. JNCI: Journal of the National Cancer Institute. 2019;111(9):916-922.
59. Samala RK, Chan H-P, Hadjiiski L, Helvie MA, Wei J, Cha K. Mass detection in digital breast tomosynthesis: Deep convolutional neural network with transfer learning from mammography. Medical Physics. 2016;43(12):6654-6666.
60. Samala RK, Chan HP, Hadjiiski LM, Helvie MA, Cha KH, Richter CD. Multi-task transfer learning deep convolutional neural network: application to computer-aided diagnosis of breast cancer on mammograms. Phys Med Biol. 2017;62(23):8894-8908.
61. Samala RK, Chan HP, Hadjiiski LM, Helvie MA, Richter C, Cha K. Evolutionary pruning of transfer learned deep convolutional neural network for breast cancer diagnosis in digital breast tomosynthesis. Phys Med Biol. 2018;63(9):095005.
62. Samala RK, Heang-Ping C, Hadjiiski L, Helvie MA, Richter CD, Cha KH. Breast Cancer Diagnosis in Digital Breast Tomosynthesis: Effects of Training Sample Size on Multi-Stage Transfer Learning Using Deep Neural Nets. IEEE Trans Med Imaging. 2019;38(3):686-696.
63. Seung Yeon S, Soochahn L, Il Dong Y, Sun Mi K, Kyoung Mu L. Joint Weakly and Semi-Supervised Deep Learning for Localization and Classification of Masses in Breast Ultrasound Images. IEEE Trans Med Imaging. 2019;38(3):762-774.
64. Shen L, Margolies LR, Rothstein JH, Fluder E, McBride R, Sieh W. Deep Learning to Improve Breast Cancer Detection on Screening Mammography. Scientific Reports. 2019;9(1):12495.
65. Stoffel E, Becker AS, Wurnig MC, et al. Distinction between phyllodes tumor and fibroadenoma in breast ultrasound using deep learning image analysis. Eur J Radiol Open. 2018;5:165-170.
66. Sun W, Tseng T-L, Zhang J, Qian W. Enhancing deep convolutional neural network scheme for breast cancer diagnosis with unlabeled data. Computerized Medical Imaging and Graphics. 2017;57:4-9.
67. Tanaka H, Chiu S-W, Watanabe T, Kaoku S, Yamaguchi T. Computer-aided diagnosis system for breast ultrasound images using deep learning. Ultrasound in Medicine & Biology. 2019;45:S4.
68. Tao C, Chen K, Han L, et al. New one-step model of breast tumor locating based on deep learning. J Xray Sci Technol. 2019;27(5):839-856.
69. Teare P, Fishman M, Benzaquen O, Toledano E, Elnekave E. Malignancy Detection on Mammography Using Dual Deep Convolutional Neural Networks and Genetically Discovered False Color Input Enhancement. Journal of digital imaging. 2017;30(4):499-505.
70. Truhn D, Schrading S, Haarburger C, Schneider H, Merhof D, Kuhl C. Radiomic versus Convolutional Neural Networks Analysis for Classification of Contrast-enhancing Lesions at Multiparametric Breast MRI. Radiology. 2019;290(2):290-297.
71. Wang H, Feng J, Zhang Z, et al. Breast mass classification via deeply integrating the contextual information from multi-view data. Pattern Recognition. 2018;80:42-52.
72. Wang J, Nishikawa RM, Yang Y. Global detection approach for clustered microcalcifications in mammograms using a deep learning network. J Med Imaging (Bellingham). 2017;4(2):024501.
73. Wang J, Yang X, Cai H, Tan W, Jin C, Li L. Discrimination of Breast Cancer with Microcalcifications on Mammography by Deep Learning. Scientific Reports. 2016;6:27327.

74. Wang J, Yang Y. A context-sensitive deep learning approach for microcalcification detection in mammograms. Pattern Recognition. 2018;78:12-22.
75. Wu N, Phang J, Park J, et al. Deep Neural Networks Improve Radiologists' Performance in Breast Cancer Screening. IEEE Transactions on Medical Imaging. 2019:1-1.
76. Xiao T, Liu L, Li K, Qin W, Yu S, Li Z. Comparison of Transferred Deep Neural Networks in Ultrasonic Breast Masses Discrimination. Biomed Res Int. 2018;2018:4605191.
77. Yala A, Lehman C, Schuster T, Portnoi T, Barzilay R. A Deep Learning Mammography-based Model for Improved Breast Cancer Risk Prediction. Radiology. 2019;292(1):60-66.
78. Yala A, Schuster T, Miles R, Barzilay R, Lehman C. A Deep Learning Model to Triage Screening Mammograms: A Simulation Study. Radiology. 2019;293(1):38-46.
79. Yap MH, Goyal M, Osman FM, et al. Breast ultrasound lesions recognition: end-to-end deep learning approaches. J Med Imaging (Bellingham). 2019;6(1):011007.
80. Yap MH, Pons G, Marti J, et al. Automated Breast Ultrasound Lesions Detection Using Convolutional Neural Networks. IEEE J Biomed Health Inform. 2018;22(4):1218-1226.
81. Yousefi M, Krzyzak A, Suen CY. Mass detection in digital breast tomosynthesis data using convolutional neural networks and multiple instance learning. Comput Biol Med. 2018;96:283-293.
82. Zhou J, Luo LY, Dou Q, et al. Weakly supervised 3D deep learning for breast cancer classification and localization of the lesions in MR images. J Magn Reson Imaging. 2019;50(4):1144-1151.

Supplementary References 4: Full list of included studies in all other specialities

1. Adams M, Chen W, Holcdorf D, McCusker MW, Howe PD, Gaillard F. Computer vs human: Deep learning versus perceptual training for the detection of neck of femur fractures. Journal of Medical Imaging and Radiation Oncology. 2019;63(1):27-32.
2. Akhavan Aghdam M, Sharifi A, Pedram MM. Combination of rs-fMRI and sMRI Data to Discriminate Autism Spectrum Disorders in Young Children Using Deep Belief Network. Journal of digital imaging. 2018;31(6):895-903.
3. Aldoj N, Lukas S, Dewey M, Penzkofer T. Semi-automatic classification of prostate cancer on multi-parametric MR imaging using a multi-channel 3D convolutional neural network. Eur Radiol. 2019.
4. Alkadi R, Taher F, El-Baz A, Werghi N. A Deep Learning-Based Approach for the Detection and Localization of Prostate Cancer in T2 Magnetic Resonance Images. Journal of digital imaging. 2019;32(5):793-807.
5. Amoroso N, Diacono D, Fanizzi A, et al. Deep learning reveals Alzheimer's disease onset in MCI subjects: Results from an international challenge. J Neurosci Methods. 2018;302:3-9.
6. Arbabshirani MR, Fornwalt BK, Mongelluzzo GJ, et al. Advanced machine learning in action: identification of intracranial 18aemorrhage on computed tomography scans of the head with clinical workflow integration. Npj Digital Medicine. 2018;1(1):9.
7. Ariji Y, Fukuda M, Kise Y, et al. Contrast-enhanced computed tomography image assessment of cervical lymph node metastasis in patients with oral cancer by using a deep learning system of artificial intelligence. Oral Surg Oral Med Oral Pathol Oral Radiol. 2019;127(5):458-463.
8. Ariji Y, Sugita Y, Nagao T, et al. CT evaluation of extranodal extension of cervical lymph node metastases in patients with oral squamous cell carcinoma using deep learning classification. Oral Radiol. 2019.
9. Ariji Y, Yanashita Y, Kutsuna S, et al. Automatic detection and classification of radiolucent lesions in the mandible on panoramic radiographs using a deep learning object detection technique. Oral Surgery, Oral Medicine, Oral Pathology and Oral Radiology. 2019;128(4):424-430.
10. Atici MA, Sagiroglu S, Celtikci P, et al. A Novel Deep Learning Algorithm for the Automatic Detection of High-Grade Gliomas on T2-Weighted Magnetic Resonance Images: A Preliminary Machine Learning Study. Turk Neurosurg. 2019.
11. Azizi S, Bayat S, Yan P, et al. Deep Recurrent Neural Networks for Prostate Cancer Detection: Analysis of Temporal Enhanced Ultrasound. IEEE Transactions on Medical Imaging. 2018;37(12):2695-2703.
12. Azizi S, Bayat S, Yan P, et al. Detection and grading of prostate cancer using temporal enhanced ultrasound: combining deep neural networks and tissue mimicking simulations. Int J Comput Assist Radiol Surg. 2017;12(8):1293-1305.
13. Azizi S, Van Woudenberg N, Sojoudi S, et al. Toward a real-time system for temporal enhanced ultrasound-guided prostate biopsy. Int J Comput Assist Radiol Surg. 2018;13(8):1201-1209.
14. Akatsuka J, Yamamoto Y, Sekine T, et al. Illuminating Clues of Cancer Buried in Prostate MR Image: Deep Learning and Expert Approaches. Biomolecules. 2019;9(11):673.
15. Badgeley MA, Zech JR, Oakden-Rayner L, et al. Deep learning predicts hip fracture using confounding patient and healthcare variables. Npj Digital Medicine. 2019;2(1):31.
16. Basaia S, Agosta F, Wagner L, et al. Automated classification of Alzheimer's disease and mild cognitive impairment using a single MRI and deep neural networks. NeuroImage Clinical. 2019;21:101645.
17. Bi X, Li S, Xiao B, Li Y, Wang G, Ma X. Computer aided Alzheimer's disease diagnosis by an unsupervised deep learning technology. Neurocomputing. 2019.
18. Bi X-a, Jiang Q, Sun Q, Shu Q, Liu Y. Analysis of Alzheimer's Disease Based on the Random Neural Network Cluster in fMRI. Frontiers in Neuroinformatics. 2018;12(60).
19. Bien N, Rajpurkar P, Ball RL, et al. Deep-learning-assisted diagnosis for knee magnetic resonance imaging: Development and retrospective validation of MRNet. PloS Med. 2018;15(11):e1002699.
20. Bijay Dev KM, Jogi PS, Niyas S, Vinayagamani S, Kesavadas C, Rajan J. Automatic detection and localization of Focal Cortical Dysplasia lesions in MRI using fully convolutional neural network. Biomedical Signal Processing and Control. 2019;52:218-225.
21. Blanc-Durand P, Van Der Gucht A, Schaefer N, Itti E, Prior JO. Automatic lesion detection and segmentation of 18F-FET PET in gliomas: A full 3D U-Net convolutional neural network study. PLOS ONE. 2018;13(4):e0195798.
22. Blau N, Klang E, Kiryati N, Amitai M, Portnoy O, Mayer A. Fully automatic detection of renal cysts in abdominal CT scans. Int J Comput Assist Radiol Surg. 2018;13(7):957-966.
23. Böhle M, Eitel F, Weygandt M, Ritter K. Layer-Wise Relevance Propagation for Explaining Deep Neural Network Decisions in MRI-Based Alzheimer's Disease Classification. Frontiers in Aging Neuroscience. 2019;11(194).

24. Brinker TJ, Hekler A, Enk AH, et al. A convolutional neural network trained with dermoscopic images performed on par with 145 dermatologists in a clinical melanoma image classification task. European Journal of Cancer. 2019;111:148-154.
25. Brinker TJ, Hekler A, Enk AH, et al. Deep learning outperformed 136 of 157 dermatologists in a head-to-head dermoscopic melanoma image classification task. Eur J Cancer. 2019;113:47-54.
26. Buda M, Wildman-Tobriner B, Hoang JK, et al. Management of Thyroid Nodules Seen on US Images: Deep Learning May Match Performance of Radiologists. Radiology. 2019;292(3):695-701.
27. Burlina P, Billings S, Joshi N, Albayda J. Automated diagnosis of myositis from muscle ultrasound: Exploring the use of machine learning and deep learning methods. PLOS ONE. 2017;12(8):e0184059.
28. Byra M, Styczynski G, Szmigielski C, et al. Transfer learning with deep convolutional neural network for liver steatosis assessment in ultrasound images. Int J Comput Assist Radiol Surg. 2018;13(12):1895-1903.
29. Cai J, Xing F, Batra A, et al. Texture analysis for muscular dystrophy classification in MRI with improved class activation mapping. Pattern Recognition. 2019;86:368-375.
30. Cao R, Bajgiran AM, Mirak SA, et al. Joint Prostate Cancer Detection and Gleason Score Prediction in mp-MRI via FocalNet. IEEE Transactions on Medical Imaging. 2019;38(11):2496-2506.
31. Cao W, An X, Cong L, Lyu C, Zhou Q, Guo R. Application of Deep Learning in Quantitative Analysis of 2-Dimensional Ultrasound Imaging of Nonalcoholic Fatty Liver Disease. Journal of Ultrasound in Medicine. 2020;39(1):51-59.
32. Ceschin R, Zahner A, Reynolds W, et al. A computational framework for the detection of subcortical brain dysmaturation in neonatal MRI using 3D Convolutional Neural Networks. NeuroImage. 2018;178:183-197.
33. Chang PD, Kuoy E, Grinband J, et al. Hybrid 3D/2D Convolutional Neural Network for Hemorrhage Evaluation on Head CT. AJNR Am J Neuroradiol. 2018;39(9):1609-1616.
34. Chang PD, Wong TT, Rasiej MJ. Deep Learning for Detection of Complete Anterior Cruciate Ligament Tear. Journal of digital imaging. 2019;32(6):980-986.
35. Charron O, Lallement A, Jarnet D, Noblet V, Clavier J-B, Meyer P. Automatic detection and segmentation of brain metastases on multimodal MR images with a deep convolutional neural network. Computers in Biology and Medicine. 2018;95:43-54.
36. Chee CG, Kim Y, Kang Y, et al. Performance of a Deep Learning Algorithm in Detecting Osteonecrosis of the Femoral Head on Digital Radiography: A Comparison With Assessments by Radiologists. American Journal of Roentgenology. 2019;213(1):155-162.
37. Chen PJ, Lin MC, Lai MJ, Lin JC, Lu HH, Tseng VS. Accurate Classification of Diminutive Colorectal Polyps Using Computer-Aided Analysis. Gastroenterology. 2018;154(3):568-575.
38. Chen Q, Hu S, Long P, Lu F, Shi Y, Li Y. A Transfer Learning Approach for Malignant Prostate Lesion Detection on Multiparametric MRI. Technology in cancer research & treatment. 2019;18:1533033819858363-1533033819858363.
39. Chen W, Liu B, Peng S, Sun J, Qiao X. Computer-Aided Grading of Gliomas Combining Automatic Segmentation and Radiomics. International Journal of Biomedical Imaging. 2018;2018:11.
40. Cheng C-T, Ho T-Y, Lee T-Y, et al. Application of a deep learning algorithm for detection and visualization of hip fractures on plain pelvic radiographs. European radiology. 2019;29(10):5469-5477.
41. Cheng PM, Tejura TK, Tran KN, Whang G. Detection of high-grade small bowel obstruction on conventional radiography with convolutional neural networks. Abdom Radiol (NY). 2018;43(5):1120-1127.
42. Cheng PM, Tran KN, Whang G, Tejura TK. Refining Convolutional Neural Network Detection of Small-Bowel Obstruction in Conventional Radiography. American Journal of Roentgenology. 2018;212(2):342-350.
43. Chi J, Walia E, Babyn P, Wang J, Groot G, Eramian M. Thyroid Nodule Classification in Ultrasound Images by Fine-Tuning Deep Convolutional Neural Network. Journal of digital imaging. 2017;30(4):477-486.
44. Chilamkurthy S, Ghosh R, Tanamala S, et al. Deep learning algorithms for detection of critical findings in head CT scans: a retrospective study. Lancet. 2018;392(10162):2388-2396.
45. Cho J, Park K-S, Karki M, et al. Improving Sensitivity on Identification and Delineation of Intracranial Hemorrhage Lesion Using Cascaded Deep Learning Models. Journal of digital imaging. 2019;32(3):450-461.
46. Choi JW, Cho YJ, Lee S, et al. Using a Dual-Input Convolutional Neural Network for Automated Detection of Pediatric Supracondylar Fracture on Conventional Radiography. Invest Radiol. 2020;55(2):101-110.
47. Choi KJ, Jang JK, Lee SS, et al. Development and Validation of a Deep Learning System for Staging Liver Fibrosis by Using Contrast Agent–enhanced CT Images in the Liver. Radiology. 2018;289(3):688-697.
48. Chung SW, Han SS, Lee JW, et al. Automated detection and classification of the proximal humerus fracture by using deep learning algorithm. Acta orthopaedica. 2018;89(4):468-473.
49. Codella NC, Nguyen Q-B, Pankanti S, et al. Deep learning ensembles for melanoma recognition in dermoscopy images. IBM Journal of Research and Development. 2017;61(4/5):5: 1-5: 15.

50. Corral JE, Hussein S, Kandel P, Bolan CW, Bagci U, Wallace MB. Deep Learning to Classify Intraductal Papillary Mucinous Neoplasms Using Magnetic Resonance Imaging. Pancreas. 2019;48(6):805-810.
51. Couteaux V, Si-Mohamed S, Nempont O, et al. Automatic knee meniscus tear detection and orientation classification with Mask-RCNN. Diagnostic and Interventional Imaging. 2019;100(4):235-242.
52. Coy H, Hsieh K, Wu W, et al. Deep learning and radiomics: the utility of Google TensorFlow Inception in classifying clear cell renal cell carcinoma and oncocytoma on multiphasic CT. Abdom Radiol (NY). 2019;44(6):2009-2020.
53. Cui R, Liu M. RNN-based longitudinal analysis for diagnosis of Alzheimer's disease. Computerized Medical Imaging and Graphics. 2019;73:1-10.
54. Cui R, Liu M. Hippocampus Analysis by Combination of 3-D DenseNet and Shapes for Alzheimer's Disease Diagnosis. IEEE J Biomed Health Inform. 2019;23(5):2099-2107.
55. Dai Y, Tao Z, Wang Y, Zhao Y, Hou J. The Research of Multi-Modality Parkinson's Disease Image Based on Cross-Layer Convolutional Neural Network. Journal of Medical Imaging and Health Informatics. 2019;9(7):1440-1447.
56. Das A, Acharya UR, Panda SS, Sabut S. Deep learning based liver cancer detection using watershed transform and Gaussian mixture model techniques. Cognitive Systems Research. 2019;54:165-175.
57. Debats OA, Litjens GJS, Huisman HJ. Lymph node detection in MR Lymphography: false positive reduction using multi-view convolutional neural networks. PeerJ. 2019;7:e8052.
58. Deepak S, Ameer PM. Brain tumor classification using deep CNN features via transfer learning. Computers in Biology and Medicine. 2019;111:103345.
59. Derkatch S, Kirby C, Kimelman D, Jozani MJ, Davidson JM, Leslie WD. Identification of Vertebral Fractures by Convolutional Neural Networks to Predict Nonvertebral and Hip Fractures: A Registry-based Cohort Study of Dual X-ray Absorptiometry. Radiology. 2019;293(2):405-411.
60. Ding L, Liu G-W, Zhao B-C, et al. Artificial intelligence system of faster region-based convolutional neural network surpassing senior radiologists in evaluation of metastatic lymph nodes of rectal cancer. Chin Med J (Engl). 2019;132(4):379-387.
61. Ding Y, Sohn JH, Kawczynski MG, et al. A Deep Learning Model to Predict a Diagnosis of Alzheimer Disease by Using (18)F-FDG PET of the Brain. Radiology. 2019;290(2):456-464.
62. Diniz PHB, Valente TLA, Diniz JOB, et al. Detection of white matter lesion regions in MRI using SLIC0 and convolutional neural network. Computer Methods and Programs in Biomedicine. 2018;167:49-63.
63. Duan H, Huang Y, Liu L, Dai H, Chen L, Zhou L. Automatic detection on intracranial aneurysm from digital subtraction angiography with cascade convolutional neural networks. BioMedical Engineering OnLine. 2019;18(1):110.
64. Duc NT, Ryu S, Qureshi MNI, Choi M, Lee KH, Lee B. 3D-Deep Learning Based Automatic Diagnosis of Alzheimer's Disease with Joint MMSE Prediction Using Resting-State fMRI. Neuroinformatics. 2019.
65. Duraisamy B, Shanmugam JV, Annamalai J. Alzheimer disease detection from structural MR images using FCM based weighted probabilistic neural network. Brain Imaging Behav. 2019;13(1):87-110.
66. Eitel F, Soehler E, Bellmann-Strobl J, et al. Uncovering convolutional neural network decisions for diagnosing multiple sclerosis on conventional MRI using layer-wise relevance propagation. NeuroImage: Clinical. 2019;24:102003.
67. Ekert T, Krois J, Meinhold L, et al. Deep Learning for the Radiographic Detection of Apical Lesions. Journal of Endodontics. 2019;45(7):917-922.e915.
68. England JR, Gross JS, White EA, Patel DB, England JT, Cheng PM. Detection of Traumatic Pediatric Elbow Joint Effusion Using a Deep Convolutional Neural Network. AJR Am J Roentgenol. 2018;211(6):1361-1368.
69. Esteva A, Kuprel B, Novoa RA, et al. Dermatologist-level classification of skin cancer with deep neural networks. Nature. 2017;542(7639):115-118.
70. Faron A, Sichtermann T, Teichert N, et al. Performance of a Deep-Learning Neural Network to Detect Intracranial Aneurysms from 3D TOF-MRA Compared to Human Readers. Clin Neuroradiol. 2019.
71. Frid-Adar M, Diamant I, Klang E, Amitai M, Goldberger J, Greenspan H. GAN-based synthetic medical image augmentation for increased CNN performance in liver lesion classification. Neurocomputing. 2018;321:321-331.
72. Fujisawa Y, Otomo Y, Ogata Y, et al. Deep-learning-based, computer-aided classifier developed with a small dataset of clinical images surpasses board-certified dermatologists in skin tumour diagnosis. The British journal of dermatology. 2019;180(2):373-381.
73. Gan K, Xu D, Lin Y, et al. Artificial intelligence detection of distal radius fractures: a comparison between the convolutional neural network and professional assessments. Acta orthopaedica. 2019;90(4):394-400.

74. Gao X, Wang X. Performance of deep learning for differentiating pancreatic diseases on contrast-enhanced magnetic resonance imaging: A preliminary study. Diagnostic and Interventional Imaging. 2019.
75. Gao X, Wang X. Deep learning for World Health Organization grades of pancreatic neuroendocrine tumors on contrast-enhanced magnetic resonance images: a preliminary study. Int J Comput Assist Radiol Surg. 2019;14(11):1981-1991.
76. Gao XW, Hui R, Tian Z. Classification of CT brain images based on deep learning networks. Computer Methods and Programs in Biomedicine. 2017;138:49-56.
77. Gao Y, Zhang ZD, Li S, et al. Deep neural network-assisted computed tomography diagnosis of metastatic lymph nodes from gastric cancer. Chin Med J (Engl). 2019.
78. Ghafoorian M, Karssemeijer N, Heskes T, et al. Deep multi-scale location-aware 3D convolutional neural networks for automated detection of lacunes of presumed vascular origin. NeuroImage: Clinical. 2017;14:391-399.
79. Ginat DT. Analysis of head CT scans flagged by deep learning software for acute intracranial 21aemorrhage. Neuroradiology. 2019.
80. Gorji HT, Kaabouch N. A Deep Learning approach for Diagnosis of Mild Cognitive Impairment Based on MRI Images. Brain Sci. 2019;9(9):217.
81. Grovik E, Yi D, Iv M, Tong E, Rubin D, Zaharchuk G. Deep learning enables automatic detection and segmentation of brain metastases on multisequence MRI. J Magn Reson Imaging. 2020;51(1):175-182.
82. Guan Q, Wang Y, Du J, et al. Deep learning based classification of ultrasound images for thyroid nodules: a large scale of pilot study. Ann Transl Med. 2019;7(7):137.
83. Haenssle HA, Fink C, Schneiderbauer R, et al. Man against machine: diagnostic performance of a deep learning convolutional neural network for dermoscopic melanoma recognition in comparison to 58 dermatologists. Annals of Oncology. 2018:mdy166-mdy166.
84. Hallac RR, Lee J, Pressler M, Seaward JR, Kane AA. Identifying Ear Abnormality from 2D Photographs Using Convolutional Neural Networks. Scientific Reports. 2019;9(1):18198.
85. Hamm CA, Wang CJ, Savic LJ, et al. Deep learning for liver tumor diagnosis part I: development of a convolutional neural network classifier for multi-phasic MRI. Eur Radiol. 2019;29(7):3338-3347.
86. Han S, Hwang SI, Lee HJ. The Classification of Renal Cancer in 3-Phase CT Images Using a Deep Learning Method. Journal of digital imaging. 2019;32(4):638-643.
87. Han SS, Kim MS, Lim W, Park GH, Park I, Chang SE. Classification of the Clinical Images for Benign and Malignant Cutaneous Tumors Using a Deep Learning Algorithm. Journal of Investigative Dermatology. 2018;138(7):1529-1538.
88. Han SS, Moon IJ, Lim W, et al. Keratinocytic Skin Cancer Detection on the Face Using Region-Based Convolutional Neural Network. JAMA Dermatology. 2019.
89. Harris RJ, Kim S, Lohr J, et al. Classification of Aortic Dissection and Rupture on Post-contrast CT Images Using a Convolutional Neural Network. Journal of digital imaging. 2019;32(6):939-946.
90. Hosny KM, Kassem MA, Foaud MM. Classification of skin lesions using transfer learning and augmentation with Alex-net. PloS One. 2019;14(5):e0217293.
91. Hosseini-Asl E, Ghazal M, Mahmoud A, et al. Alzheimer's disease diagnostics by a 3D deeply supervised adaptable convolutional network. Front Biosci (Landmark Ed). 2018;23:584-596.
92. Huang Y, Xu J, Zhou Y, Tong T, Zhuang X. Diagnosis of Alzheimer's Disease via Multi-Modality 3D Convolutional Neural Network. Front Neurosci. 2019;13:509.
93. Hussein S, Kandel P, Bolan CW, Wallace MB, Bagci U. Lung and Pancreatic Tumor Characterization in the Deep Learning Era: Novel Supervised and Unsupervised Learning Approaches. IEEE Transactions on Medical Imaging. 2019;38(8):1777-1787.
94. Ishioka J, Matsuoka Y, Uehara S, et al. Computer-aided diagnosis of prostate cancer on magnetic resonance imaging using a convolutional neural network algorithm. BJU international. 2018;122(3):411-417.
95. Abdelaziz Ismael SA, Mohammed A, Hefny H. An enhanced deep learning approach for brain cancer MRI images classification using residual networks. Artificial Intelligence in Medicine. 2020;102:101779.
96. Jain R, Jain N, Aggarwal A, Hemanth DJ. Convolutional neural network based Alzheimer's disease classification from magnetic resonance brain images. Cognitive Systems Research. 2019;57:147-159.
97. Kann BH, Hicks DF, Payabvash S, et al. Multi-Institutional Validation of Deep Learning for Pretreatment Identification of Extranodal Extension in Head and Neck Squamous Cell Carcinoma. Journal of Clinical Oncology. 2019:JCO.19.02031.
98. Ker J, Singh SP, Bai Y, Rao J, Lim T, Wang L. Image Thresholding Improves 3-Dimensional Convolutional Neural Network Diagnosis of Different Acute Brain Hemorrhages on Computed Tomography Scans. Sensors (Basel, Switzerland). 2019;19(9):2167.
99. Kim DH, MacKinnon T. Artificial intelligence in fracture detection: transfer learning from deep convolutional neural networks. Clinical radiology. 2018;73(5):439-445.

100. Kim DH, Wit H, Thurston M. Artificial intelligence in the diagnosis of Parkinson's disease from ioflupane-123 single-photon emission computed tomography dopamine transporter scans using transfer learning. Nuclear Medicine Communications. 2018;39(10):887-893.
101. Kim H-G, Lee KM, Kim EJ, Lee JS. Improvement diagnostic accuracy of sinusitis recognition in paranasal sinus X-ray using multiple deep learning models. Quantitative imaging in medicine and surgery. 2019;9(6):942-951.
102. Kim K, Kim S, Lee YH, Lee SH, Lee HS, Kim S. Performance of the deep convolutional neural network based magnetic resonance image scoring algorithm for differentiating between tuberculous and pyogenic spondylitis. Scientific Reports. 2018;8(1):13124.
103. Kim S, Yoon H, Lee M-J, et al. Performance of deep learning-based algorithm for detection of ileocolic intussusception on abdominal radiographs of young children. Scientific Reports. 2019;9(1):19420.
104. Kim T, Heo J, Jang D-K, et al. Machine learning for detecting moyamoya disease in plain skull radiography using a convolutional neural network. EbioMedicine. 2019;40:636-642.
105. Kim Y, Lee KJ, Sunwoo L, et al. Deep Learning in Diagnosis of Maxillary Sinusitis Using Conventional Radiography. Invest Radiol. 2019;54(1):7-15.
106. Kise Y, Ikeda H, Fujii T, et al. Preliminary study on the application of deep learning system to diagnosis of Sjogren's syndrome on CT images. Dentomaxillofac Radiol. 2019;48(6):20190019.
107. Kise Y, Shimizu M, Ikeda H, et al. Usefulness of a deep learning system for diagnosing Sjögren's syndrome using ultrasonography images. Dentomaxillofacial Radiology. 2019:20190348.
108. Kitamura G, Chung CY, Moore BE. Ankle Fracture Detection Utilizing a Convolutional Neural Network Ensemble Implemented with a Small Sample, De Novo Training, and Multiview Incorporation. Journal of digital imaging. 2019;32(4):672-677.
109. Ko SY, Lee JH, Yoon JH, et al. Deep convolutional neural network for the diagnosis of thyroid nodules on ultrasound. Head & Neck. 2019;41(4):885-891.
110. Kuo W, Häne C, Mukherjee P, Malik J, Yuh EL. Expert-level detection of acute intracranial 22aemorrhage on head computed tomography using deep learning. Proceedings of the National Academy of Sciences. 2019;116(45):22737-22745.
111. Kutlu H, Avci E. A Novel Method for Classifying Liver and Brain Tumors Using Convolutional Neural Networks, Discrete Wavelet Transform and Long Short-Term Memory Networks. Sensors (Basel). 2019;19(9).
112. Le MH, Chen J, Wang L, et al. Automated diagnosis of prostate cancer in multi-parametric MRI based on multimodal convolutional neural networks. Physics in Medicine & Biology. 2017;62(16):6497-6514.
113. Lee H, Hong H, Kim J, Jung DC. Deep feature classification of angiomyolipoma without visible fat and renal cell carcinoma in abdominal contrast-enhanced CT images with texture image patches and hand-crafted feature concatenation. Med Phys. 2018;45(4):1550-1561.
114. Lee H, Yune S, Mansouri M, et al. An explainable deep-learning algorithm for the detection of acute intracranial haemorrhage from small datasets. Nature Biomedical Engineering. 2019;3(3):173-182.
115. Lee JH, Baek JH, Kim JH, et al. Deep Learning-Based Computer-Aided Diagnosis System for Localization and Diagnosis of Metastatic Lymph Nodes on Ultrasound: A Pilot Study. Thyroid. 2018;28(10):1332-1338.
116. Lee JH, Ha EJ, Kim JH. Application of deep learning to the diagnosis of cervical lymph node metastasis from thyroid cancer with CT. European Radiology. 2019;29(10):5452-5457.
117. Lee JH, Kim DH, Jeong SN. Diagnosis of cystic lesions using panoramic and cone beam computed tomographic images based on deep learning neural network. Oral Dis. 2020;26(1):152-158.
118. Lee JS, Adhikari S, Liu L, Jeong HG, Kim H, Yoon SJ. Osteoporosis detection in panoramic radiographs using a deep convolutional neural network-based computer-assisted diagnosis system: a preliminary study. Dentomaxillofac Radiol. 2018:20170344.
119. Li F, Tran L, Thung K, Ji S, Shen D, Li J. A Robust Deep Model for Improved Classification of AD/MCI Patients. IEEE Journal of Biomedical and Health Informatics. 2015;19(5):1610-1616.
120. Li H, Weng J, Shi Y, et al. An improved deep learning approach for detection of thyroid papillary cancer in ultrasound images. Scientific Reports. 2018;8(1):6600.
121. Li J, Wu Y, Shen N, et al. A fully automatic computer-aided diagnosis system for hepatocellular carcinoma using convolutional neural networks. Biocybernetics and Biomedical Engineering. 2020;40(1):238-248.
122. Li L, Song X, Guo Y, et al. Deep Convolutional Neural Networks for Automatic Detection of Orbital Blowout Fractures. J Craniofac Surg. 2019.
123. Li X, Zhang S, Zhang Q, et al. Diagnosis of thyroid cancer using deep convolutional neural network models applied to sonographic images: a retrospective, multicohort, diagnostic study. The Lancet Oncology. 2019;20(2):193-201.

124. Lim ZV, Akram F, Ngo CP, et al. Automated grading of acne vulgaris by deep learning with convolutional neural networks. Skin Res Technol. 2019.
125. Lin W, Tong T, Gao Q, et al. Convolutional Neural Networks-Based MRI Image Analysis for the Alzheimer's Disease Prediction From Mild Cognitive Impairment. Frontiers in Neuroscience. 2018;12(777).
126. Lindsey R, Daluiski A, Chopra S, et al. Deep neural network improves fracture detection by clinicians. Proceedings of the National Academy of Sciences of the United States of America. 2018;115(45):11591-11596.
127. Liu F, Zhou Z, Samsonov A, et al. Deep Learning Approach for Evaluating Knee MR Images: Achieving High Diagnostic Performance for Cartilage Lesion Detection. Radiology. 2018;289(1):160-169.
128. Liu J, Wang D, Lu L, et al. Detection and diagnosis of colitis on computed tomography using deep convolutional neural networks. Med Phys. 2017;44(9):4630-4642.
129. Liu M, Cheng D, Wang K, Wang Y. Multi-Modality Cascaded Convolutional Neural Networks for Alzheimer's Disease Diagnosis. Neuroinformatics. 2018;16(3-4):295-308.
130. Liu M, Cheng D, Yan W. Classification of Alzheimer's Disease by Combination of Convolutional and Recurrent Neural Networks Using FDG-PET Images. Front Neuroinform. 2018;12:35.
131. Liu SL, Li S, Guo YT, et al. Establishment and application of an artificial intelligence diagnosis system for pancreatic cancer with a faster region-based convolutional neural network. Chin Med J (Engl). 2019;132(23):2795-2803.
132. Liu T, Guo Q, Lian C, et al. Automated detection and classification of thyroid nodules in ultrasound images using clinical-knowledge-guided convolutional neural networks. Medical Image Analysis. 2019;58:101555.
133. Lu D, Popuri K, Ding GW, Balachandar R, Beg MF. Multimodal and Multiscale Deep Neural Networks for the Early Diagnosis of Alzheimer's Disease using structural MR and FDG-PET images. Sci Rep. 2018;8(1):5697.
134. Lu Y, Yu Q, Gao Y, et al. Identification of Metastatic Lymph Nodes in MR Imaging with Faster Region-Based Convolutional Neural Networks. Cancer Research. 2018;78(17):5135-5143.
135. Ma J, Wu F, Jiang T, Zhu J, Kong D. Cascade convolutional neural networks for automatic detection of thyroid nodules in ultrasound images. Med Phys. 2017;44(5):1678-1691.
136. Ma J, Wu F, Zhu J, Xu D, Kong D. A pre-trained convolutional neural network based method for thyroid nodule diagnosis. Ultrasonics. 2017;73:221-230.
137. Ma L, Ma C, Liu Y, Wang X. Thyroid Diagnosis from SPECT Images Using Convolutional Neural Network with Optimization. Comput Intell Neurosci. 2019;2019:6212759.
138. Maki S, Furuya T, Horikoshi T, et al. A Deep Convolutional Neural Network with Performance Comparable to Radiologists for Differentiating Between Spinal Schwannoma and Meningioma. Spine (Phila Pa 1976). 2019.
139. Maqsood M, Nazir F, Khan U, et al. Transfer Learning Assisted Classification and Detection of Alzheimer's Disease Stages Using 3D MRI Scans. Sensors (Basel, Switzerland). 2019;19(11):2645.
140. Maron RC, Weichenthal M, Utikal JS, et al. Systematic outperformance of 112 dermatologists in multiclass skin cancer image classification by convolutional neural networks. European Journal of Cancer. 2019;119:57-65.
141. Ahammed Muneer KV, Rajendran VR, K PJ. Glioma Tumor Grade Identification Using Artificial Intelligent Techniques. Journal of Medical Systems. 2019;43(5):113.
142. Nakao T, Hanaoka S, Nomura Y, et al. Deep neural network-based computer-assisted detection of cerebral aneurysms in MR angiography. J Magn Reson Imaging. 2018;47(4):948-953.
143. Nguyen DT, Pham TD, Batchuluun G, Yoon HS, Park KR. Artificial Intelligence-Based Thyroid Nodule Classification Using Information from Spatial and Frequency Domains. J Clin Med. 2019;8(11).
144. Oh K, Chung Y-C, Kim KW, Kim W-S, Oh I-S. Classification and Visualization of Alzheimer's Disease using Volumetric Convolutional Neural Network and Transfer Learning. Scientific Reports. 2019;9(1):18150.
145. Olczak J, Fahlberg N, Maki A, et al. Artificial intelligence for 23aemorrha 23aemorrhage trauma radiographs. Acta orthopaedica. 2017;88(6):581-586.
146. Öman O, Mäkelä T, Salli E, Savolainen S, Kangasniemi M. 3D convolutional neural networks applied to CT angiography in the detection of acute ischemic stroke. European Radiology Experimental. 2019;3(1):8.
147. Ortiz A, Munilla J, Gorriz JM, Ramirez J. Ensembles of Deep Learning Architectures for the Early Diagnosis of the Alzheimer's Disease. Int J Neural Syst. 2016;26(7):1650025.
148. Ortiz-Ramon R, Valdes Hernandez MDC, Gonzalez-Castro V, et al. Identification of the presence of ischaemic stroke lesions by means of texture analysis on brain magnetic resonance images. Comput Med Imaging Graph. 2019;74:12-24.
149. Özyurt F, Sert E, Avcı D. An expert system for brain tumor detection: Fuzzy C-means with super resolution and convolutional neural network with extreme learning machine. Medical Hypotheses. 2020;134:109433.

150. Pang S, Ding T, Qiao S, et al. A novel YOLOv3-arch model for identifying cholelithiasis and classifying gallstones on CT images. PLOS ONE. 2019;14(6):e0217647.
151. Pang S, Wang S, Rodríguez-Patón A, Li P, Wang X. An artificial intelligent diagnostic system on mobile Android terminals for cholelithiasis by lightweight convolutional neural network. PLOS ONE. 2019;14(9):e0221720.
152. Park A, Chute C, Rajpurkar P, et al. Deep Learning-Assisted Diagnosis of Cerebral Aneurysms Using the HeadXNet Model. JAMA Netw Open. 2019;2(6):e195600.
153. Park VY, Han K, Seong YK, et al. Diagnosis of Thyroid Nodules: Performance of a Deep Learning Convolutional Neural Network Model vs. Radiologists. Scientific Reports. 2019;9(1):17843.
154. Phillips M, Marsden H, Jaffe W, et al. Assessment of Accuracy of an Artificial Intelligence Algorithm to Detect Melanoma in Images of Skin Lesions. JAMA Netw Open. 2019;2(10):e1913436.
155. Pinto dos Santos D, Brodehl S, Baeßler B, et al. Structured report data can be used to develop deep learning algorithms: a proof of concept in ankle radiographs. Insights into Imaging. 2019;10(1):93.
156. Podgorsak AR, Rava RA, Shiraz Bhurwani MM, et al. Automatic radiomic feature extraction using deep learning for angiographic parametric imaging of intracranial aneurysms. J Neurointerv Surg. 2019.
157. Poedjiastoeti W, Suebnukarn S. Application of Convolutional Neural Network in the Diagnosis of Jaw Tumors. Healthc Inform Res. 2018;24(3):236-241.
158. Pranata YD, Wang K-C, Wang J-C, et al. Deep learning and SURF for automated classification and detection of calcaneus fractures in CT images. Computer Methods and Programs in Biomedicine. 2019;171:27-37.
159. Qin P, Wu K, Hu Y, Zeng J, Chai X. Diagnosis of benign and malignant thyroid nodules using combined conventional ultrasound and ultrasound elasticity imaging. IEEE J Biomed Health Inform. 2019.
160. Dou Q, Yu L, Chen H, et al. 3D deeply supervised network for automated segmentation of volumetric medical images. Medical Image Analysis. 2017;41:40-54.
161. Ramzan F, Khan MUG, Rehmat A, et al. A Deep Learning Approach for Automated Diagnosis and Multi-Class Classification of Alzheimer's Disease Stages Using Resting-State fMRI and Residual Neural Networks. Journal of Medical Systems. 2019;44(2):37.
162. Roblot V, Giret Y, Bou Antoun M, et al. Artificial intelligence to diagnose meniscus tears on MRI. Diagnostic and Interventional Imaging. 2019;100(4):243-249.
163. Salvador R, Canales-Rodríguez E, Guerrero-Pedraza A, et al. Multimodal Integration of Brain Images for MRI-Based Diagnosis in Schizophrenia. Frontiers in Neuroscience. 2019;13(1203).
164. Schelb P, Kohl S, Radtke JP, et al. Classification of Cancer at Prostate MRI: Deep Learning versus Clinical PI-RADS Assessment. Radiology. 2019;293(3):607-617.
165. Schmauch B, Herent P, Jehanno P, et al. Diagnosis of focal liver lesions from ultrasound using deep learning. Diagn Interv Imaging. 2019;100(4):227-233.
166. Seetha J, Raja S. Brain Tumor Classification Using Convolutional Neural Networks. Biomedical and Pharmacology Journal. 2018;11:1457-1461.
167. Shen T, Jiang J, Lin W, et al. Use of Overlapping Group LASSO Sparse Deep Belief Network to Discriminate Parkinson's Disease and Normal Control. Front Neurosci. 2019;13:396.
168. Shi J, Zheng X, Li Y, Zhang Q, Ying S. Multimodal Neuroimaging Feature Learning With Multimodal Stacked Deep Polynomial Networks for Diagnosis of Alzheimer's Disease. IEEE J Biomed Health Inform. 2018;22(1):173-183.
169. Shinohara Y, Takahashi N, Lee Y, Ohmura T, Kinoshita T. Development of a deep learning model to identify hyperdense MCA sign in patients with acute ischemic stroke. Jpn J Radiol. 2019.
170. Sibille L, Seifert R, Avramovic N, et al. 18F-FDG PET/CT Uptake Classification in Lymphoma and Lung Cancer by Using Deep Convolutional Neural Networks. Radiology.0(0):191114.
171. Sichtermann T, Faron A, Sijben R, Teichert N, Freiherr J, Wiesmann M. Deep Learning-Based Detection of Intracranial Aneurysms in 3D TOF-MRA. AJNR Am J Neuroradiol. 2019;40(1):25-32.
172. Song J, Chai YJ, Masuoka H, et al. Ultrasound image analysis using deep learning algorithm for the diagnosis of thyroid nodules. Medicine. 2019;98(15):e15133-e15133.
173. Song W, Li S, Liu J, et al. Multitask Cascade Convolution Neural Networks for Automatic Thyroid Nodule Detection and Recognition. IEEE Journal of Biomedical and Health Informatics. 2019;23(3):1215-1224.
174. Song Y, Zhang YD, Yan X, et al. Computer-aided diagnosis of prostate cancer using a deep convolutional neural network from multiparametric MRI. J Magn Reson Imaging. 2018;48(6):1570-1577.
175. Stember JN, Chang P, Stember DM, et al. Convolutional Neural Networks for the Detection and Measurement of Cerebral Aneurysms on Magnetic Resonance Angiography. Journal of digital imaging. 2019;32(5):808-815.
176. Streba CT, Ionescu M, Gheonea DI, et al. Contrast-enhanced ultrasonography parameters in neural network diagnosis of liver tumors. World J Gastroenterol. 2012;18(32):4427-4434.

177. Suk H-I, Lee S-W, Shen D, Alzheimer's Disease Neuroimaging I. Hierarchical feature representation and multimodal fusion with deep learning for AD/MCI diagnosis. NeuroImage. 2014;101:569-582.
178. Suk HI, Lee SW, Shen D. Latent feature representation with stacked auto-encoder for AD/MCI diagnosis. Brain Struct Funct. 2015;220(2):841-859.
179. Sumathipala Y, Lay N, Turkbey B, Smith C, Choyke PL, Summers RM. Prostate cancer detection from multi-institution multiparametric MRIs using deep convolutional neural networks. J Med Imaging (Bellingham). 2018;5(4):044507.
180. Świetlik D, Białowąs J. Application of Artificial Neural Networks to Identify Alzheimer's Disease Using Cerebral Perfusion SPECT Data. International journal of environmental research and public health. 2019;16(7):1303.
181. Talo M, Baloglu UB, Yıldırım Ö, Rajendra Acharya U. Application of deep transfer learning for automated brain abnormality classification using MR images. Cognitive Systems Research. 2019;54:176-188.
182. Talo M, Yildirim O, Baloglu UB, Aydin G, Acharya UR. Convolutional neural networks for multi-class brain disease detection using MRI images. Computerized Medical Imaging and Graphics. 2019;78:101673.
183. Tekchandani H, Verma S, Londhe ND. Mediastinal lymph node malignancy detection in computed tomography images using fully convolutional network. Biocybernetics and Biomedical Engineering. 2020;40(1):187-199.
184. Thian YL, Li Y, Jagmohan P, Sia D, Chan VEY, Tan RT. Convolutional Neural Networks for Automated Fracture Detection and Localization on Wrist Radiographs. Radiology: Artificial Intelligence. 2019;1(1):e180001.
185. Titano JJ, Badgeley M, Schefflein J, et al. Automated deep-neural-network surveillance of cranial images for acute neurologic events. Nature Medicine. 2018;24(9):1337-1341.
186. Tiulpin A, Thevenot J, Rahtu E, Lehenkari P, Saarakkala S. Automatic Knee Osteoarthritis Diagnosis from Plain Radiographs: A Deep Learning-Based Approach. Scientific Reports. 2018;8(1):1727.
187. Togo R, Yamamichi N, Mabe K, et al. Detection of gastritis by a deep convolutional neural network from double-contrast upper gastrointestinal barium X-ray radiography. J Gastroenterol. 2019;54(4):321-329.
188. Tomita N, Cheung YY, Hassanpour S. Deep neural networks for automatic detection of osteoporotic vertebral fractures on CT scans. Comput Biol Med. 2018;98:8-15.
189. Trivizakis E, Manikis GC, Nikiforaki K, et al. Extending 2-D Convolutional Neural Networks to 3-D for Advancing Deep Learning Cancer Classification With Application to MRI Liver Tumor Differentiation. IEEE J Biomed Health Inform. 2019;23(3):923-930.
190. Tschandl P, Rosendahl C, Akay BN, et al. Expert-Level Diagnosis of Nonpigmented Skin Cancer by Combined Convolutional Neural Networks. JAMA Dermatol. 2019;155(1):58-65.
191. Ueda D, Yamamoto A, Nishimori M, et al. Deep Learning for MR Angiography: Automated Detection of Cerebral Aneurysms. Radiology. 2019;290(1):187-194.
192. Urakawa T, Tanaka Y, Goto S, Matsuzawa H, Watanabe K, Endo N. Detecting intertrochanteric hip fractures with 25aemorrhage25-level accuracy using a deep convolutional neural network. Skeletal radiology. 2019;48(2):239-244.
193. Ureten K, Erbay H, Maras HH. Detection of rheumatoid arthritis from hand radiographs using a convolutional neural network. Clin Rheumatol. 2019.
194. Varuna Shree N, Kumar TNR. Identification and classification of brain tumor MRI images with feature extraction using DWT and probabilistic neural network. Brain Informatics. 2018;5(1):23-30.
195. Wang H, Zhou Z, Li Y, et al. Comparison of machine learning methods for classifying mediastinal lymph node metastasis of non-small cell lung cancer from 18F-FDG PET/CT images. EJNMMI Research. 2017;7(1):11.
196. Wang J, Fang Z, Lang N, Yuan H, Su MY, Baldi P. A multi-resolution approach for spinal metastasis detection using deep Siamese neural networks. Comput Biol Med. 2017;84:137-146.
197. Wang L, Yang S, Yang S, et al. Automatic thyroid nodule recognition and diagnosis in ultrasound imaging with the YOLOv2 neural network. World Journal of Surgical Oncology. 2019;17(1):12.
198. Wang X, Yang W, Weinreb J, et al. Searching for prostate cancer by fully automated magnetic resonance imaging classification: deep learning versus non-deep learning. Sci Rep. 2017;7(1):15415.
199. Wang YM, Li Y, Cheng YS, et al. Deep Learning in Automated Region Proposal and Diagnosis of Chronic Otitis Media Based on Computed Tomography. Ear Hear. 2019.
200. Wang Z, Liu C, Cheng D, Wang L, Yang X, Cheng KT. Automated Detection of Clinically Significant Prostate Cancer in mp-MRI Images Based on an End-to-End Deep Neural Network. IEEE Trans Med Imaging. 2018;37(5):1127-1139.
201. Wee C-Y, Liu C, Lee A, Poh JS, Ji H, Qiu A. Cortical graph neural network for AD and MCI diagnosis and transfer learning across populations. NeuroImage: Clinical. 2019;23:101929.

202. Winkel DJ, Heye T, Weikert TJ, Boll DT, Stieltjes B. Evaluation of an AI-Based Detection Software for Acute Findings in Abdominal Computed Tomography Scans: Toward an Automated Work List Prioritization of Routine CT Examinations. Invest Radiol. 2019;54(1):55-59.
203. Xu H, Baxter JSH, Akin O, Cantor-Rivera D. Prostate cancer detection using residual networks. Int J Comput Assist Radiol Surg. 2019;14(10):1647-1650.
204. Xu L, Tetteh G, Lipkova J, et al. Automated Whole-Body Bone Lesion Detection for Multiple Myeloma on 68Ga-Pentixafor PET/CT Imaging Using Deep Learning Methods. Contrast Media & Molecular Imaging. 2018;2018:11.
205. Xue Y, Zhang R, Deng Y, Chen K, Jiang T. A preliminary examination of the diagnostic value of deep learning in hip osteoarthritis. PLOS ONE. 2017;12(6):e0178992.
206. Yan K, Wang X, Lu L, Summers RM. DeepLesion: automated mining of large-scale lesion annotations and universal lesion detection with deep learning. Journal of Medical Imaging. 2018;5(3):036501.
207. Yang J, Zhang K, Fan H, et al. Development and validation of deep learning algorithms for scoliosis screening using back images. Communications Biology. 2019;2(1):390.
208. Yang X, Liu C, Wang Z, et al. Co-trained convolutional neural networks for automated detection of prostate cancer in multi-parametric MRI. Med Image Anal. 2017;42:212-227.
209. Yang Y, Yan L-F, Zhang X, et al. Glioma Grading on Conventional MR Images: A Deep Learning Study With Transfer Learning. Frontiers in neuroscience. 2018;12:804-804.
210. Yao D, Calhoun VD, Fu Z, Du Y, Sui J. An ensemble learning system for a 4-way classification of Alzheimer's disease and mild cognitive impairment. Journal of Neuroscience Methods. 2018;302:75-81.
211. Yap J, Yolland W, Tschandl P. Multimodal skin lesion classification using deep learning. Exp Dermatol. 2018;27(11):1261-1267.
212. Yasaka K, Akai H, Abe O, Kiryu S. Deep Learning with Convolutional Neural Network for Differentiation of Liver Masses at Dynamic Contrast-enhanced CT: A Preliminary Study. Radiology. 2018;286(3):887-896.
213. Ye H, Gao F, Yin Y, et al. Precise diagnosis of intracranial 26aemorrhage and subtypes using a three-dimensional joint convolutional and recurrent neural network. Eur Radiol. 2019;29(11):6191-6201.
214. Yoo S, Gujrathi I, Haider MA, Khalvati F. Prostate Cancer Detection using Deep Convolutional Neural Networks. Scientific Reports. 2019;9(1):19518.
215. Yu C, Yang S, Kim W, et al. Acral melanoma detection using a convolutional neural network for dermoscopy images. PLOS ONE. 2018;13(3):e0193321.
216. Yu JS, Yu SM, Erdal BS, et al. Detection and localisation of hip fractures on anteroposterior radiographs with artificial intelligence: proof of concept. Clinical radiology. 2019.
217. Yu Q, Jiang T, Zhou A, Zhang L, Zhang C, Xu P. Computer-aided diagnosis of malignant or benign thyroid nodes based on ultrasound images. European Archives of Oto-Rhino-Laryngology. 2017;274(7):2891-2897.
218. Yuan Y, Qin W, Buyyounouski M, et al. Prostate cancer classification with multiparametric MRI transfer learning model. Med Phys. 2019;46(2):756-765.
219. Zhang S, Han F, Liang Z, et al. An investigation of CNN models for differentiating malignant from benign lesions using small pathologically proven datasets. Computerized Medical Imaging and Graphics. 2019;77:101645.
220. Zhou L, Zhang Z, Chen Y-C, Zhao Z-Y, Yin X-D, Jiang H-B. A Deep Learning-Based Radiomics Model for Differentiating Benign and Malignant Renal Tumors. Transl Oncol. 2019;12(2):292-300.
221. Zhu L-C, Ye Y-L, Luo W-H, et al. A model to discriminate malignant from benign thyroid nodules using artificial neural network. PloS one. 2013;8(12):e82211-e82211.
222. Zhu Y, Wang L, Liu M, et al. MRI-based prostate cancer detection with high-level representation and hierarchical classification. Med Phys. 2017;44(3):1028-1039.
223. Zreik M, Hamersvelt RWv, Wolterink JM, Leiner T, Viergever MA, Išgum I. A Recurrent CNN for Automatic Detection and Classification of Coronary Artery Plaque and Stenosis in Coronary CT Angiography. IEEE Transactions on Medical Imaging. 2019;38(7):1588-1598.

Supplementary Table 1: Characteristics of included studies from all other specialities

Study	Model	Imaging Modality	Speciality	Disease	AI vs clinician?
Adams et al. 2018	GoogLeNet	X-ray	Orthopaedics	Neck of femur fractures	Yes
Akhavan Aghdam et al. 2018	Deep Belief Network	MRI	Neurology/Neurosurgery	Autism Spectrum Disorders	No
Aldoj et al. 2019	3D CNN	MRI	Urology	Prostate Cancer	No
Alkadi et al. 2018	Deep Convolutional Encoder-Decoder	MRI	Urology	Prostate Cancer	No
Amoroso et al. 2018	DNN	MRI	Neurology/Neurosurgery	Alzheimer's Disease	No
Arbabshirani et al. 2018	3D-CNN	CT	Neurology/Neurosurgery	Intracranial haemorrhage	No
Ariji et al. 2019	DetectNet	Panoramic Radiographs	Maxillofacial Surgery	Radiolucent lesions of Mandible	No
Ariji et al. 2019	CNN	CT	Maxillofacial Surgery	Cervical lymph node metastasis	Yes
Ariji et al. 2019	AlexNet	CT	Maxillofacial Surgery	Cervical lymph node metastasis	Yes
Atici et al. 2019	CNN	MRI	Neurology/Neurosurgery	Glioma	No
Atsuka et al. 2019	Xception	MRI	Urology	Prostate Cancer	No
Azizi et al. 2017	CNN	Ultrasound	Urology	Prostate Cancer	No
Azizi et al. 2018	LSTM	Ultrasound	Urology	Prostate Cancer	No
Azizi et al. 2018	CNN	Ultrasound	Urology	Prostate Cancer	No
Badgeley et al. 2019	CNN	X-ray	Orthopaedics	Hip fractures	No
Basaia et al. 2019	CNN	MRI	Neurology/Neurosurgery	Alzheimer's Disease	No
Bi et al. 2018	Elman Neural Network	fMRI	Neurology/Neurosurgery	Alzheimer's Disease	No
Bi et al. 2019	CNN	MRI	Neurology/Neurosurgery	Alzheimer's Disease	No
Bien et al. 2018	MRNet	MRI	Orthopaedics	Anterior Cruciate Ligament Tear	Yes
Bijay Dev et al. 2019	U-net	MRI	Neurology/Neurosurgery	Focal Cortical Dysplasia	No
Blanc-Durand et al. 2018	3D U-net	PET	Neurology/Neurosurgery	Glioma	No
Blau et al. 2018	3D FCN	CT	Urology	Renal cysts	No
Bohle et al. 2019	CNN	MRI	Neurology/Neurosurgery	Alzheimer's Disease	No
Brinker et al. 2019	CNN	Dermascopic images	Dermatology	Melanoma	Yes
Brinker et al. 2019	CNN	Photographs	Dermatology	Melanoma	Yes
Buda et al. 2019	CNN	Ultrasound	Endocrine	Thyroid nodules	Yes
Burlina et al. 2017	DL-DCNN	Ultrasound	Rheumatology	Myositis	No
Byra et al. 2018	Inception-ResNet-v2	Ultrasound	Gastroenterology/Hepatology	Liver steatosis	No
Cai et al. 2019	CNN	MRI	Rheumatology	Muscular Dystrophy	No
Cao et al. 2019	CNN	Ultrasound	Gastroenterology/Hepatology	NAFLD	No
Cao et al. 2019	FocalNet	MRI	Urology	Prostate Cancer	Yes

Supplementary Table 1: Characteristics of included studies from all other specialities

Study	Model	Imaging Modality	Speciality	Disease	AI vs clinician?
Ceschin et al. 2018	3D CNN	MRI	Neurology/Neurosurgery	Subcortical brain dysmaturation	No
Chang et al. 2018	CNN	CT	Neurology/Neurosurgery	Intracranial haemorrhage	No
Chang et al. 2019	CNN	MRI	Orthopaedics	Anterior Cruciate Ligament Tear	No
Charron et al. 2018	DeepMedic	MRI	Neurology/Neurosurgery	Brain Metastases	No
Chee et al. 2019	ResNet	X-ray	Orthopaedics	Osteonecrosis of femoral head	Yes
Chen et al. 2018	DNN	Photographs	Gastroenterology/Hepatology	Colorectal polyps	Yes
Chen et al. 2018	3D CNN	MRI	Neurology/Neurosurgery	Glioma	No
Chen et al. 2019	VGG-13	MRI	Urology	Prostate Cancer	No
Cheng et al. 2018	InceptionV3	X-ray	Gastroenterology/Hepatology	Small bowel obstruction	No
Cheng et al. 2019	DCNN	X-ray	Orthopaedics	Hip fractures	No
Cheng et al. 2019	CNN	X-ray	GI Surgery	Small bowel obstruction	No
Chi et al. 2017	GoogLeNet	Ultrasound	Endocrine	Thyroid nodules	No
Chilamkurthy et al. 2018	CNN	CT	Neurology/Neurosurgery	Head CT abnormalities	No
Cho et al. 2019	CNN	CT	Neurology/Neurosurgery	Intracranial haemorrhage	No
Choi et al. 2019	CNN	CT	Gastroenterology/Hepatology	Liver fibrosis	Yes
Choi et al. 2019	ResNet-50	X-ray	Orthopaedics	Supracondylar fracture	Yes
Chung et al. 2018	CNN	X-ray	Orthopaedics	Proximal humerus fracture	Yes
Codella et al. 2017	U-net	Photographs	Dermatology	Melanoma	Yes
Corral et al. 2019	CNN	MRI	Gastroenterology/Hepatology	Intraductal Papillary Mucinous Neoplasia	No
Couteaux et al. 2019	Mask-RCNN	MRI	Orthopaedics	Knee meniscus tear	No
Coy et al. 2019	InceptionV3	CT	Urology	Renal tumours	No
Cui et al. 2018	3D DenseNet	MRI	Neurology/Neurosurgery	Alzheimer's Disease	No
Cui et al. 2019	RNN	MRI	Neurology/Neurosurgery	Alzheimer's Disease	No
Dai et al. 2019	CNN	MRI	Neurology/Neurosurgery	Parkinson's	No
Das et al. 2019	Watershed Gaussian based deep learning (WGDL)	CT	Gastroenterology/Hepatology	Liver Tumours	No
Debats et al. 2019	3D CNN	MRI	Oncology	Lymph node detection	No
Deepak et al. 2019	GoogLeNet	MRI	Neurology/Neurosurgery	Brain Tumours	No
Derkatch et al. 2019	CNN	X-ray	Orthopaedics	Vertebral fractures	No
Ding et al. 2019	InceptionV3	FDG-PET	Neurology/Neurosurgery	Alzheimer's Disease	Yes
Ding et al. 2019	Faster R-CNN	MRI	Oncology	Metastatic lymph nodes of rectal cancer	Yes
Diniz et al. 2018	CNN	MRI	Neurology/Neurosurgery	White matter lesions	No

Supplementary Table 1: Characteristics of included studies from all other specialities

Study	Model	Imaging Modality	Speciality	Disease	AI vs clinician?
Duan et al. 2019	CNN	Digital Subtraction Angiography	Neurology/Neurosurgery	Intracranial aneurysms	No
Duc et al. 2019	3D CNN	MRI	Neurology/Neurosurgery	Alzheimer's Disease	No
Duraisamy et al. 2019	FCM based weighted probabilistic neural network	MRI	Neurology/Neurosurgery	Alzheimer's Disease	No
Eitel et al. 2019	3D CNN	MRI	Neurology/Neurosurgery	Multiple Sclerosis	No
Ekert et al. 2019	CNN	Panoramic Radiographs	Maxillofacial Surgery	Apical lesions	No
England et al. 2018	DCNN	X-ray	Orthopaedics	Elbow joint effusion	No
Esteva et al. 2017	CNN	Photographs	Dermatology	Skin tumours	Yes
Faron et al. 2019	CNN	MRA	Neurology/Neurosurgery	Intracranial aneurysms	Yes
Frid-Adar et al. 2018	CNN	CT	Gastroenterology/Hepatology	Liver Tumours	No
Fujisawa et al. 2018	DCNN	Photographs	Dermatology	Skin tumours	Yes
Gan et al. 2019	CNN	X-ray	Orthopaedics	Distal radius fractures	Yes
Gao et al. 2017	Fused CNN	CT	Neurology/Neurosurgery	Alzheimer's Disease	No
Gao et al. 2019	Inceptionv4	MRI	Gastroenterology/Hepatology	Pancreatic Diseases	No
Gao et al. 2019	CNN	MRI	Gastroenterology/Hepatology	Pancreatic neuroendocrine tumours	No
Gao et al. 2019	Faster R-CNN	CT	Oncology	Peri gastric metastatic lymph nodes	No
Ghafoorian et al. 2017	3D CNN	MRI	Neurology/Neurosurgery	Lacunes	Yes
Ginat et al. 2019	Aidoc	CT	Neurology/Neurosurgery	Intracranial haemorrhage	No
Gorji et al. 2019	CNN	MRI	Neurology/Neurosurgery	Alzheimer's Disease	No
Grovik et al. 2019	CNN	MRI	Neurology/Neurosurgery	Brain Metastases	No
Guan et al. 2019	InceptionV3	Ultrasound	Endocrine	Thyroid nodules	No
Haenssle et al. 2018	CNN	Photographs	Dermatology	Melanoma	Yes
Hallac et al. 2019	GoogLeNet	Photographs	ENT	Ear Abnormality	No
Hamm et al. 2019	Custom CNN	MRI	Gastroenterology/Hepatology	Liver Tumours	Yes
Han et al. 2018	ResNet 152	Photographs	Dermatology	Skin tumours	Yes
Han et al. 2019	ResNet-50	Photographs	Dermatology	Skin tumours	Yes
Han et al. 2019	GoogLeNet	CT	Urology	Renal tumours	No
Harris et al. 2019	CNN	CT	Vascular Surgery	Aortic Dissection and Rupture	No
Hosny et al. 2019	AlexNet	Photographs	Dermatology	Skin tumours	No
Hosseini-Asl et al. 2018	3D CNN	MRI	Neurology/Neurosurgery	Alzheimer's Disease	No
Huang et al. 2019	3D CNN	MRI	Neurology/Neurosurgery	Alzheimer's Disease	No
Hussein et al. 2019	VGG-Net	MRI	Gastroenterology/Hepatology	Intraductal Papillary Mucinous Neoplasia	No

Supplementary Table 1: Characteristics of included studies from all other specialities

Study	Model	Imaging Modality	Speciality	Disease	AI vs clinician?
Ishioka et al. 2018	CNN	MRI	Urology	Prostate Cancer	No
Ismael et al. 2019	ResNet	MRI	Neurology/Neurosurgery	Brain Tumours	No
Jain et al. 2019	VGG-16	MRI	Neurology/Neurosurgery	Alzheimer's Disease	No
Kann et al. 2019	CNN	CT	Maxillofacial Surgery	Extranodal extension in head and neck squamous cell carcinoma	Yes
Ker et al. 2019	3d CNN	CT	Neurology/Neurosurgery	Intracranial haemorrhage	No
Kim et al. 2018	InceptionV3	SPECT	Neurology/Neurosurgery	Parkinson's	No
Kim et al. 2018	InceptionV3	X-ray	Orthopaedics	Fractures	No
Kim et al. 2019	ResNet	Radiographs	Maxillofacial Surgery	Maxillary Sinusitis	Yes
Kim et al. 2019	ResNet-101	X-ray	Maxillofacial Surgery	Sinusitis	No
Kim et al. 2019	CNN	X-ray	Maxillofacial Surgery	Moyamoya disease	No
Kim et al. 2019	DCNN	MRI	Orthopaedics	Spondylitis	Yes
Kim et al. 2019	YOLOv3	X-ray	GI Surgery	Ileocolic Intussusception	No
Kise et al. 2019	CNN	CT	Rheumatology	Sjogren's disease	Yes
Kise et al. 2019	CNN	Ultrasound	Rheumatology	Sjogren's disease	Yes
Kitamura et al. 2019	Inception V3, ResNet, Xception	X-ray	Orthopaedics	Ankle fracture	No
Ko et al. 2019	CNN	Ultrasound	Endocrine	Thyroid nodules	Yes
Kuo et al. 2019	CNN	CT	Neurology/Neurosurgery	Intracranial haemorrhage	Yes
Kutlu et al. 2019 (a)	CNN	CT	Gastroenterology/Hepatology	Liver Tumours	No
Kutlu et al. 2019 (b)	CNN	MRI	Neurology/Neurosurgery	Brain Tumours	No
Le et al. 2017	Multimodal CNN	MRI	Urology	Prostate Cancer	No
Lee et al. 2018	DCNN	X-ray	Metabolic Medicine	Osteoporosis	No
Lee et al. 2018	VGG-Net	Ultrasound	Oncology	Cervical lymph node metastasis	No
Lee et al. 2018	Deep Feature Classifier	CT	Urology	Renal tumours	No
Lee et al. 2019	ResNet50	CT	Endocrine	Cervical lymph node metastasis	No
Lee et al. 2019	VGG16, ResNet-50, Inception-v3, Inception-Resnet-v2	CT	Neurology/Neurosurgery	Acute Intracranial Haemorrhage	Yes
Lee et al. 2020	GoogLeNet Inception-v3	Radiographs	Maxillofacial Surgery	Odontogenic cystic lesions	No
Li et al. 2015	Restricted Boltzmann machine	MRI	Neurology/Neurosurgery	Alzheimer's Disease	No
Li et al. 2018	R-CNN	Ultrasound	Endocrine	Thyroid papillary cancer	No
Li et al. 2019	DCNN	Ultrasound	Endocrine	Thyroid cancer	Yes
Li et al. 2019	InceptionV3	CT	Maxillofacial Surgery	Orbital Blowout Fractures	No

Supplementary Table 1: Characteristics of included studies from all other specialities

Study	Model	Imaging Modality	Speciality	Disease	AI vs clinician?
Li et al. 2020	VGG-16	CT	Gastroenterology/Hepatology	Hepatocellular carcinoma	No
Lim et al. 2019	Inceptionv4	Photographs	Dermatology	Acne Vulgaris	No
Lin et al. 2018	CNN	MRI	Neurology/Neurosurgery	Alzheimer's Disease	No
Lindsey et al. 2018	CNN	X-ray	Orthopaedics	Fractures	Yes
Liu et al. 2017	Faster R-CNN	CT	Gastroenterology/Hepatology	Colitis	No
Liu et al. 2018	2D CNN	FDG-PET	Neurology/Neurosurgery	Alzheimer's Disease	No
Liu et al. 2018	3D-CNN	MRI	Neurology/Neurosurgery	Alzheimer's Disease	No
Liu et al. 2018	VGG-16	MRI	Orthopaedics	Knee cartilage lesions	No
Liu et al. 2019	CNN	Ultrasound	Endocrine	Thyroid nodules	Yes
Liu et al. 2019	VGG-16	CT	Gastroenterology/Hepatology	Pancreatic Cancer	No
Lu et al. 2018	Multimodal and multiscale deep neural network	MRI	Neurology/Neurosurgery	Alzheimer's Disease	No
Lu et al. 2018	Faster R-CNN	MRI	Oncology	Pelvic lymph node metastasis	Yes
Ma et al. 2017	CNN	Ultrasound	Endocrine	Thyroid nodules	No
Ma et al. 2017	Cascade CNN	Ultrasound	Endocrine	Thyroid nodules	No
Ma et al. 2019	DenseNet	SPECT	Endocrine	Thyroid diseases	No
Maki et al. 2019	CNN	MRI	Neurology/Neurosurgery	Spinal Schwannoma and Meningioma	Yes
Maqsood et al. 2019	AlexNet	MRI	Neurology/Neurosurgery	Alzheimer's Disease	No
Maron et al. 2019	CNN	Photographs	Dermatology	Skin tumours	Yes
Muneer et al. 2019	VGG-19	MRI	Neurology/Neurosurgery	Glioma	No
Nakao et al. 2018	CNN	MRA	Neurology/Neurosurgery	Cerebral aneurysms	No
Nguyen et al. 2019	CNN	Ultrasound	Endocrine	Thyroid nodules	No
Oh et al. 2019	Convolutional Autoencoder	MRI	Neurology/Neurosurgery	Alzheimer's Disease	No
Olczak et al. 2017	VGG-16	X-ray	Orthopaedics	Fractures	Yes
Oman et al. 2019	3D CNN	CT	Neurology/Neurosurgery	Ischaemic Stroke	No
Ortiz et al. 2016	Deep Belief Network	MRI	Neurology/Neurosurgery	Alzheimer's Disease	No
Ortiz-Ramon et al. 2019	CNN	MRI	Neurology/Neurosurgery	Ischaemic Stroke	No
Ozyurt et al. 2020	SqueezeNet	MRI	Neurology/Neurosurgery	Brain tumours	No
Pang et al. 2019	Yolov3-arch	CT	Gastroenterology/Hepatology	Cholelithiasis	No
Pang et al. 2019	MobileNetV2	CT	Gastroenterology/Hepatology	Cholelithiasis	No
Park et al. 2019	CNN	Ultrasound	Endocrine	Thyroid nodules	Yes
Park et al. 2019	HeadXNet	MRI	Neurology/Neurosurgery	Cerebral aneurysms	Yes
Phillips et al. 2019	Deep Ensemble for Recognition Malignancy	Photographs	Dermatology	Melanoma	Yes

Supplementary Table 1: Characteristics of included studies from all other specialities

Study	Model	Imaging Modality	Speciality	Disease	AI vs clinician?
Pinto dos Santos et al. 2019	InceptionV3	X-ray	Orthopaedics	Ankle fracture	No
Podgorsak et al. 2019	CNN	Angiographic parametric imaging	Neurology/Neurosurgery	Intracranial aneurysms	No
Poedjiastoeti et al. 2018	VGG-16	Panoramic Radiographs	Maxillofacial Surgery	Jaw Tumours	Yes
Pranata et al. 2019	ResNet	CT	Orthopaedics	Calcaneus fractures	No
Qi Dou et al. 2016	3D CNN	MRI	Neurology/Neurosurgery	Cerebral microbleeds	No
Qin et al. 2019	CNN	Ultrasound	Endocrine	Thyroid nodules	No
Ramzan et al. 2019	ResNet-18	MRI	Neurology/Neurosurgery	Alzheimer's Disease	No
Roblot et al. 2019	Faster R-CNN	MRI	Orthopaedics	Knee meniscus tear	No
Salvador et al. 2019	1D-CNN	MRI	Neurology/Neurosurgery	Schizophrenia	No
Schelb et al. 2019	U-net	MRI	Urology	Prostate Cancer	No
Schmauch et al. 2019	CNN	Ultrasound	Gastroenterology/Hepatology	Liver Tumours	No
Seetha et al. 2018	CNN	MRI	Neurology/Neurosurgery	Brain Tumours	No
Shen et al. 2019	Group Lasso Sparse Deep Belief Network	MRI	Neurology/Neurosurgery	Parkinson's	No
Shi et al. 2018	Deep Polynomial Network	MRI	Neurology/Neurosurgery	Alzheimer's Disease	No
Shinohara et al. 2019	Xception	CT	Neurology/Neurosurgery	Ischaemic Stroke	No
Sibille et al. 2019	CNN	PET/CT	Oncology	Lymphoma and Lung Cancer	No
Sichtermann et al. 2019	CNN	MRA	Neurology/Neurosurgery	Intracranial aneurysms	No
Song et al. 2018	InceptionV3	Ultrasound	Endocrine	Thyroid nodules	No
Song et al. 2018	VGG-Net	MRI	Urology	Prostate Cancer	No
Song et al. 2019	VGG-16	Ultrasound	Endocrine	Thyroid nodules	Yes
Stember et al. 2019	U-net	MRA	Neurology/Neurosurgery	Cerebral aneurysms	No
Streba et al. 2012	ANN	Ultrasound	Gastroenterology/Hepatology	Liver Tumours	Yes
Suk et al. 2014	Deep Boltzmann Machine	MRI	Neurology/Neurosurgery	Alzheimer's Disease	No
Suk et al. 2015	Stacked Auto-Encoder	MRI	Neurology/Neurosurgery	Alzheimer's Disease	No
Sumathipala et al. 2018	CNN	MRI	Urology	Prostate Cancer	No
Swietlik et al. 2019	ANN	SPECT	Neurology/Neurosurgery	Alzheimer's Disease	No
Talo et al. 2019	ResNet134	MRI	Neurology/Neurosurgery	Abnormal scans	No
Talo et al. 2019	ResNet-50	MRI	Neurology/Neurosurgery	Brain Tumours	No
Tekchandani et al. 2020	AlexNet	CT	Oncology	Mediastinal Lymph node metastasis	No
Thian et al. 2019	Inception-ResNet Faster R-CNN	X-ray	Orthopaedics	Wrist fractures	No

Supplementary Table 1: Characteristics of included studies from all other specialities

Study	Model	Imaging Modality	Speciality	Disease	AI vs clinician?
Titano et al. 2018	ResNet-50	CT	Neurology/Neurosurgery	Acute Neurologic Events	Yes
Tiulpin et al. 2018	Deep Siamese CNN	X-ray	Orthopaedics	Knee osteoarthritis	No
Togo et al. 2019	DCNN	Double-contrast upper gastrointestinal barium X-ray	Gastroenterology/Hepatology	Gastritis	No
Tomita et al. 2018	CNN	CT	Orthopaedics	Vertebral fractures	No
Trivizakis et al. 2019	3D CNN	MRI	Gastroenterology/Hepatology	Liver Tumours	No
Tschandl et al. 2019	InceptionV3, ResNet50	Photographs	Dermatology	Skin tumours	Yes
Ueda et al. 2019	ResNet-18	MRA	Neurology/Neurosurgery	Cerebral aneurysms	No
Urakawa et al. 2019	CNN	X-ray	Orthopaedics	Hip fractures	Yes
Ureten et al. 2019	CNN	X-ray	Rheumatology	Rheumatoid arthritis	No
Varuna Shree et al. 2018	Probabilistic neural network	MRI	Neurology/Neurosurgery	Brain Tumours	No
Wang et al. 2017	Siamese neural networks	MRI	Neurology/Neurosurgery	Spinal metastasis	No
Wang et al. 2017	CNN	FDG-PET	Oncology	Mediastinal Lymph node metastasis	Yes
Wang et al. 2017	DCNN	MRI	Urology	Prostate Cancer	No
Wang et al. 2018	CNN	MRI	Urology	Prostate Cancer	No
Wang et al. 2019	YOLOv2	Ultrasound	Endocrine	Thyroid nodules	Yes
Wang et al. 2019	InceptionV3	CT	ENT	Chronic Otitis Media	No
Wee et al. 2019	Graph CNN	MRI	Neurology/Neurosurgery	Alzheimer’s Disease	No
Winkel et al. 2019	CNN	CT	GI Surgery	Acute Abdominal Findings	No
Xu et al. 2018	CNN	PET/CT	Haematology	Multiple Myeloma	No
Xu et al. 2018	VGG-Net	MRI	Urology	Prostate Cancer	No
Xue et al. 2017	VGG-16	X-ray	Orthopaedics	Hip osteoarthritis	Yes
Yan et al. 2018	CNN	CT	Oncology	Lesions	No
Yang et al. 2017	CNN	MRI	Urology	Prostate Cancer	No
Yang et al. 2018	GoogLeNet, AlexNet	MRI	Neurology/Neurosurgery	Glioma	No
Yang et al. 2019	ResNet	Photographs	Orthopaedics	Scoliosis	No
Yao et al. 2018	CNN	MRI	Neurology/Neurosurgery	Alzheimer’s Disease	No
Yap et al. 2018	CNN	Photographs	Dermatology	Skin tumours	No
Yasaka et al. 2018	CNN	CT	Gastroenterology/Hepatology	Liver Tumours	No
Ye et al. 2019	3D CNN-RNN	CT	Neurology/Neurosurgery	Intracranial haemorrhage	Yes
Yoo et al. 2019	ResNet	MRI	Urology	Prostate Cancer	No
Yu et al. 2017	ANN	Ultrasound	Endocrine	Thyroid nodules	No
Yu et al. 2018	VGG-16	Photographs	Dermatology	Acral melanoma	Yes

Supplementary Table 1: Characteristics of included studies from all other specialities

Study	Model	Imaging Modality	Speciality	Disease	AI vs clinician?
Yu et al. 2019	InceptionV3	X-ray	Orthopaedics	Hip fractures	Yes
Yuan et al. 2019	DCNN	MRI	Urology	Prostate Cancer	No
Zhang et al. 2019	Voxel-level-1D CNN	CT	Gastroenterology/Hepatology	Colorectal polyps	No
Zhou et al. 2019	InceptionV3	CT	Urology	Renal tumours	No
Zhu et al. 2013	ANN	Ultrasound	Endocrine	Thyroid cancer	No
Zhu et al. 2017	Stacked Auto-Encoder	MRI	Urology	Prostate Cancer	No
Zreik et al. 2019	RCNN	Coronary CT Angiography	Cardiology	Coronary Artery Plaque and Stenosis	No

Supplementary Figure 1 – Summary ROC curve for six different patient cohorts to diagnose glaucoma on retinal fundus photographs from Liu et al. 2019

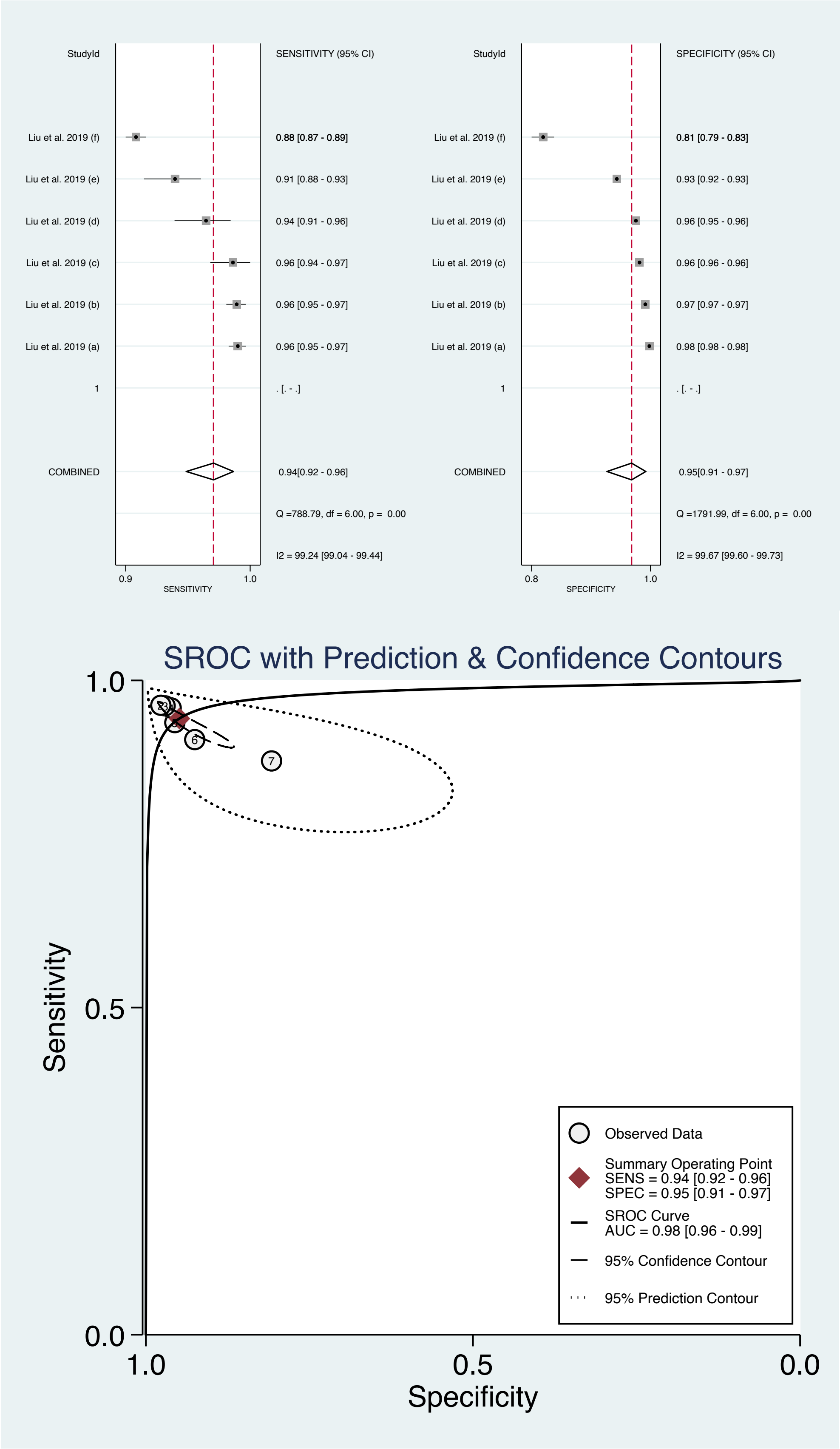

Supplementary Figure 2 – Summary ROC curve for five different patient cohorts to diagnose pneumothorax on CXR from Taylor et al. 2018 and Park et al. 2019

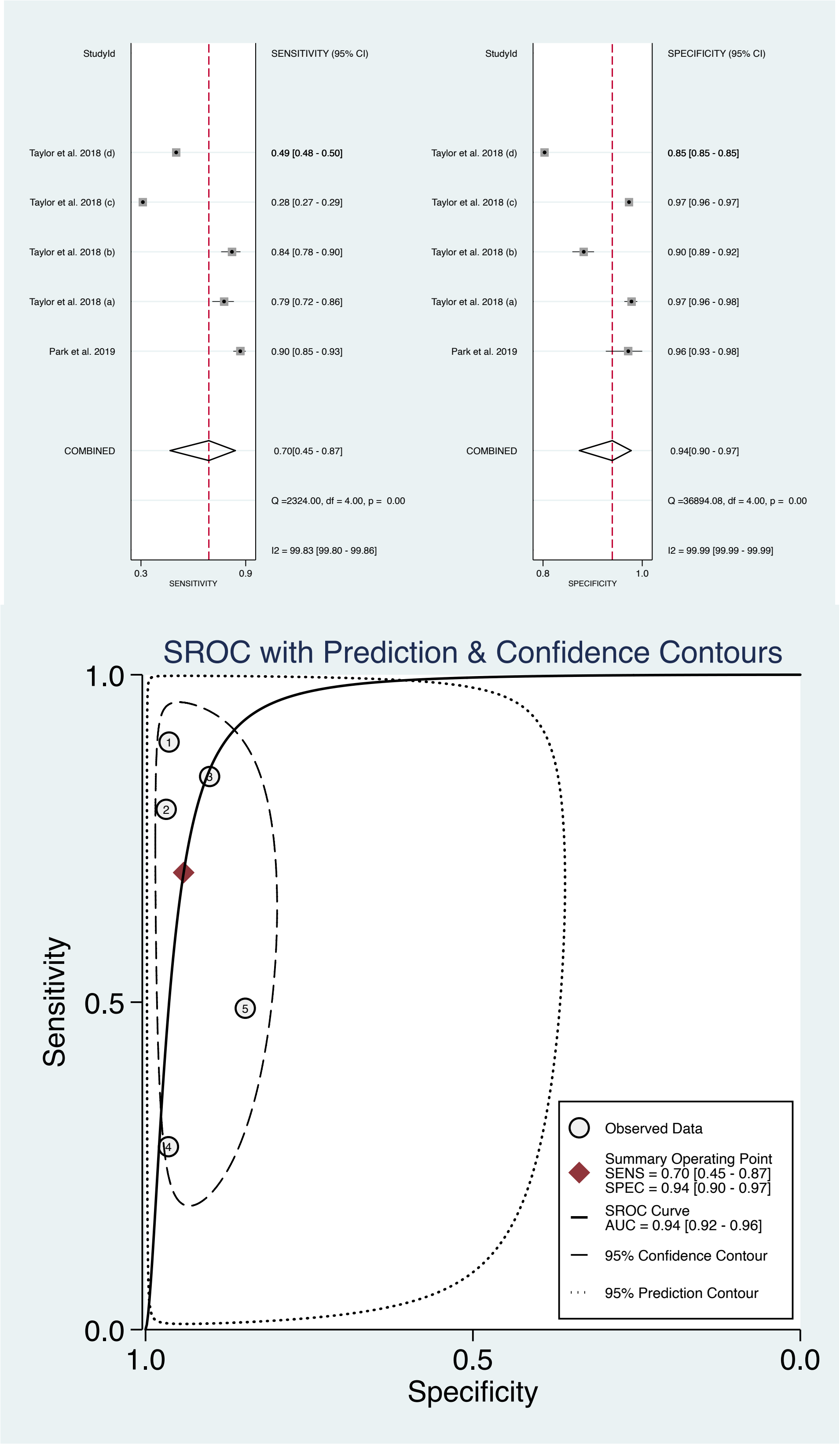

Supplementary Figure 3 – Summary ROC curve for four different patient cohorts to diagnose tuberculosis on CXR from Lakhani et al. 2017

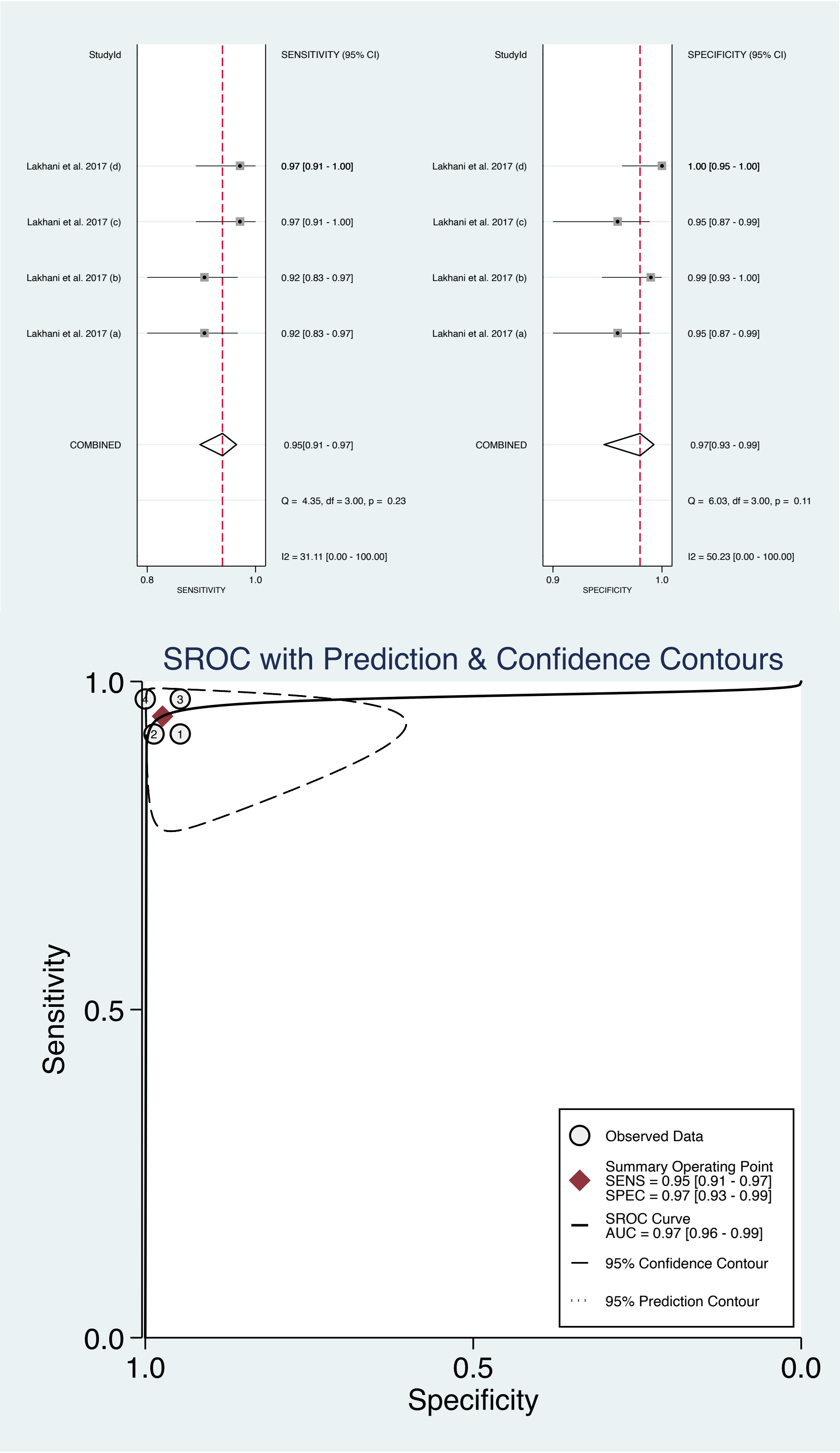

Supplementary Figure 4: Histogram demonstrating number of studies comparing algorithm performance against health-care professionals in each speciality

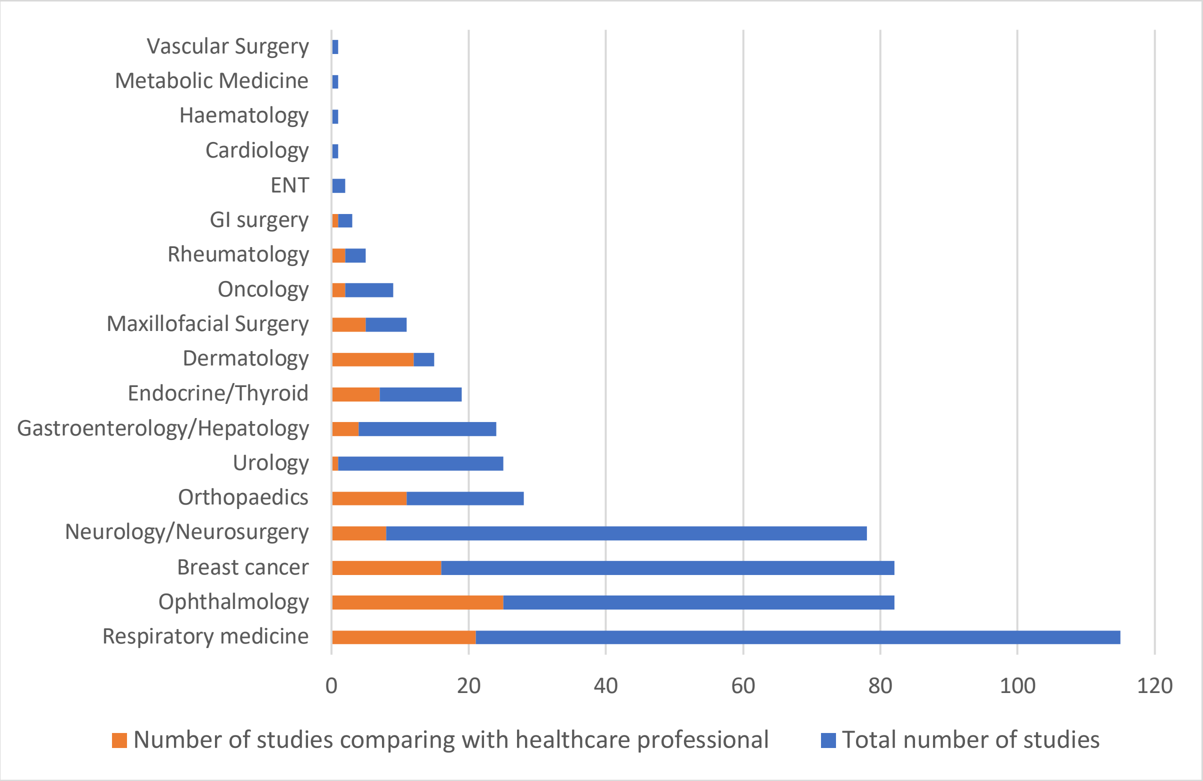

Supplementary Figure 5: Funnel Plot for all cohorts reporting on the sensitivity of deep learning to identify lung nodules on CT scans

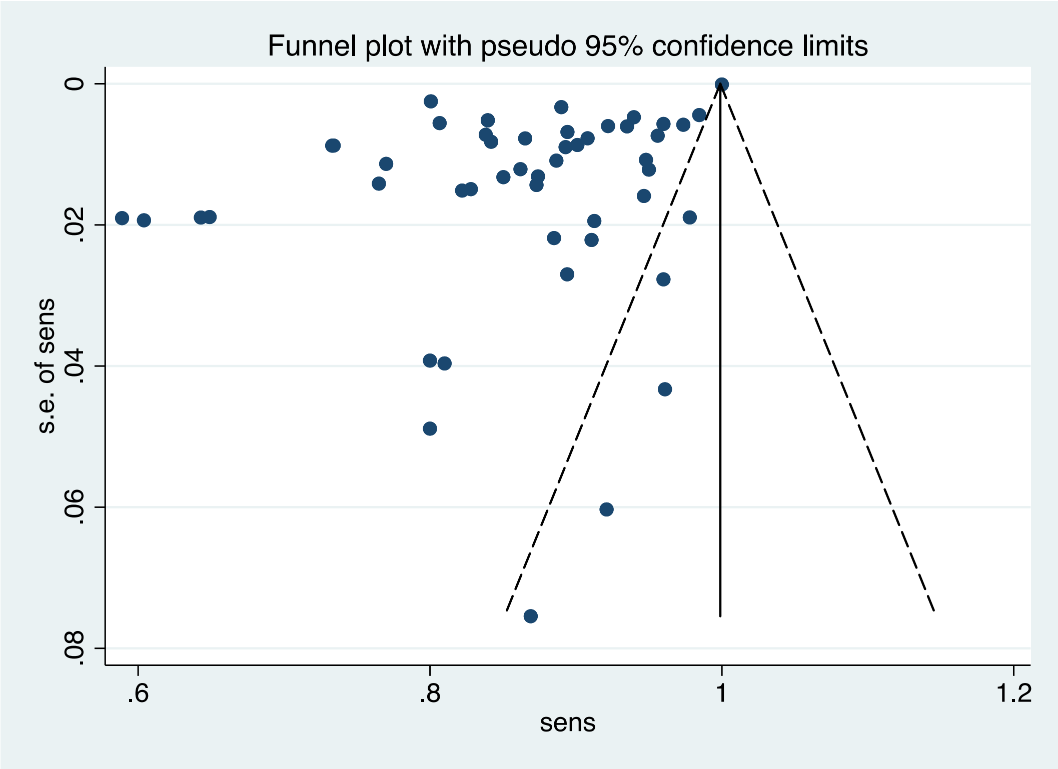


```
Egger's test for small-study effects:
Regress standard normal deviate of intervention
effect estimate against its standard error

Number of studies =   50                              Root MSE      =   13.55
```

Std_Eff	Coef.	Std. Err.	t	P>|t|	[95% Conf. Interval]	
slope	**1.001033**	**.0013817**	**724.50**	**0.000**	**.9982547**	**1.003811**
bias	**-13.91079**	**1.962083**	**-7.09**	**0.000**	**-17.85582**	**-9.965757**

Test of H0: no small-study effects   P = **0.000**

Supplementary Figure 6: Funnel Plot for all cohorts reporting on the AUC of deep learning to identify breast cancer on mammography

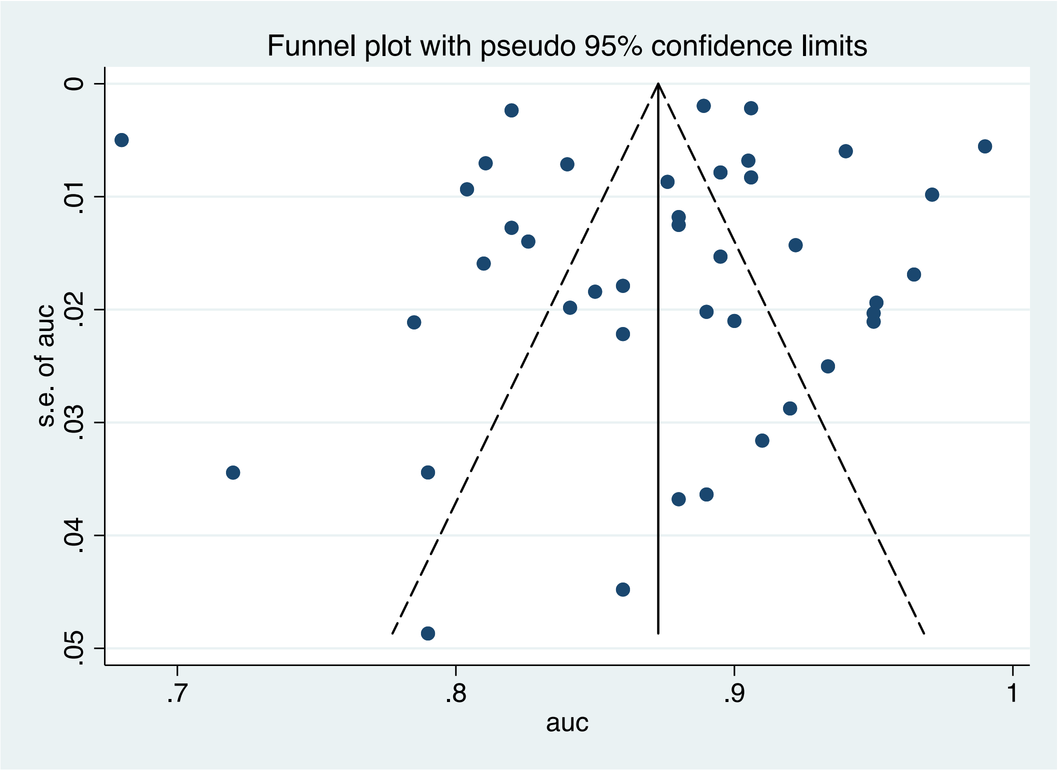


```
Note: data input format theta se_theta assumed.

Egger's test for small-study effects:
Regress standard normal deviate of intervention
effect estimate against its standard error

Number of studies =   41                              Root MSE      =    9.294
```

Std_Eff	Coef.	Std. Err.	t	P>|t|	[95% Conf.	Interval]
slope	.8728206	.0130025	67.13	0.000	.8465207	.8991206
bias	-.0356264	1.987155	-0.02	0.986	-4.055027	3.983774

```
Test of H0: no small-study effects          P = 0.986
```

Supplementary Figure 7: Funnel Plot for all cohorts reporting on the sensitivity of deep learning to identify diabetic retinopathy on retinal fundus photography

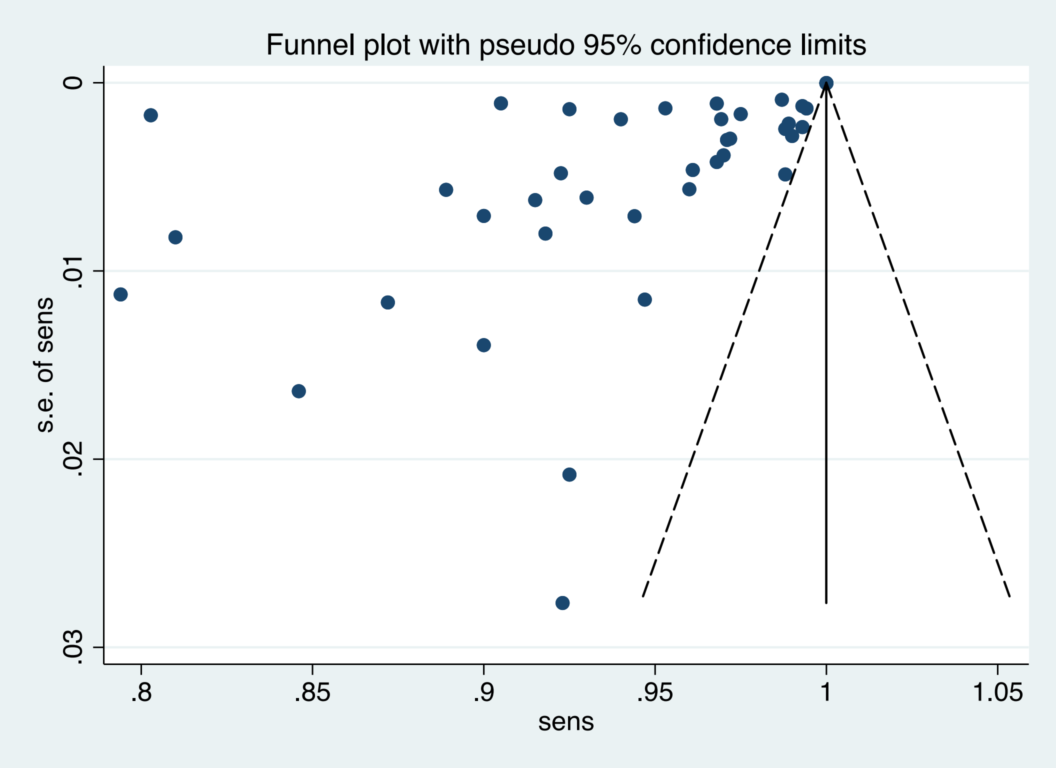


```
Note: data input format theta se_theta assumed.

Egger's test for small-study effects:
Regress standard normal deviate of intervention
effect estimate against its standard error

Number of studies =   40                              Root MSE      =    22.41
```

Std_Eff	Coef.	Std. Err.	t	P>|t|	[95% Conf. Interval]	
slope	**1.000255**	**.0002227**	**4491.40**	**0.000**	**.9998038**	**1.000705**
bias	**-17.30663**	**3.683509**	**-4.70**	**0.000**	**-24.76351**	**-9.849757**

```
Test of H0: no small-study effects          P = 0.000
```

Supplementary Figure 8 – Pooled AUC for diagnosing features of diabetic retinopathy on retinal fundus photography

Study	ES	[95% Conf.	Interval]	% Weight
Abramoff et al. 2016	0.980	0.973	0.987	2.59
Arcadu et al. 2019 (	0.940	0.927	0.953	2.56
Arcadu et al. 2019 (	0.970	0.962	0.978	2.58
Bellemo et al. 2019	0.973	0.967	0.979	2.59
Bellemo et al. 2019	0.934	0.925	0.943	2.58
Bellemo et al. 2019	0.942	0.934	0.950	2.58
Gargeya et al. 2017	0.970	0.967	0.973	2.59
Gargeya et al. 2017	0.940	0.929	0.951	2.57
Gargeya et al. 2017	0.950	0.930	0.970	2.52
Gulshan et al. 2019	0.953	0.945	0.961	2.58
Gulshan et al. 2016	0.991	0.989	0.993	2.59
Gulshan et al. 2016	0.990	0.985	0.995	2.59
Keel et al. 2018	0.933	0.882	0.984	2.19
Krause et al. 2018	0.986	0.981	0.991	2.59
Li et al. 2019	0.990	0.988	0.993	2.59
Li Z et al. 2018	0.955	0.953	0.957	2.59
Nagasawa et al. 2019	0.969	0.952	0.986	2.54
Ramachandran et al.	0.901	0.874	0.928	2.47
Ramachandran et al.	0.980	0.972	0.988	2.58
Raumviboonsuk et al.	0.987	0.986	0.988	2.59
Raumviboonsuk et al.	0.991	0.990	0.992	2.59
Raumviboonsuk et al.	0.993	0.992	0.994	2.59
Raumviboonsuk et al.	0.993	0.992	0.994	2.59
Sandhu et al. 2018	0.970	0.944	0.996	2.47
Stevenson et al. 201	0.746	0.728	0.764	2.54
Ting et al. 2017 (a)	0.936	0.934	0.938	2.59
Ting et al. 2017 (b)	0.949	0.946	0.952	2.59
Ting et al. 2017 (c)	0.889	0.878	0.900	2.57
Ting et al. 2017 (d)	0.917	0.909	0.925	2.58
Ting et al. 2017 (e)	0.919	0.907	0.931	2.57
Ting et al. 2017 (f)	0.929	0.913	0.945	2.55
Ting et al. 2017 (g)	0.980	0.974	0.986	2.59
Ting et al. 2017 (h)	0.983	0.978	0.988	2.59
Ting et al. 2017 (i)	0.950	0.938	0.962	2.57
Ting et al. 2017 (j)	0.948	0.936	0.960	2.57
Ting et al. 2017 (k)	0.964	0.960	0.968	2.59
Ting et al. 2019 (a)	0.738	0.735	0.741	2.59
Ting et al. 2019 (b)	0.795	0.792	0.798	2.59
Ting et al. 2019 (c)	0.810	0.807	0.813	2.59
D+L pooled ES	0.939	0.920	0.958	100.00

Heterogeneity chi-squared = **63746.34** (d.f. = **38**) p = **0.000**
I-squared (variation in ES attributable to heterogeneity) = **99.9%**
Estimate of between-study variance Tau-squared = **0.0036**

Test of ES=0 : z= **96.92** p = **0.000**

Supplementary Figure 9 – Pooled sensitivity for diagnosing features of diabetic retinopathy on retinal fundus photography

Study	ES	[95% Conf. Interval]		% Weight
Abramoff et al. 2016	**0.968**	**0.960**	**0.976**	**0.51**
Abramoff et al. 2018	**0.872**	**0.849**	**0.895**	**0.07**
Arcadu et al. 2019 (	**0.990**	**0.984**	**0.996**	**1.08**
Arcadu et al. 2019 (	**0.900**	**0.886**	**0.914**	**0.18**
Bellemo et al. 2019	**0.923**	**0.913**	**0.932**	**0.39**
Bellemo et al. 2019	**0.994**	**0.992**	**0.997**	**3.76**
Bellemo et al. 2019	**0.972**	**0.966**	**0.978**	**0.98**
Gargeya et al. 2017	**0.940**	**0.936**	**0.944**	**2.12**
Gargeya et al. 2017	**0.930**	**0.918**	**0.942**	**0.24**
Gargeya et al. 2017	**0.900**	**0.873**	**0.927**	**0.05**
Gulshan et al. 2019	**0.889**	**0.878**	**0.900**	**0.28**
Gulshan et al. 2016	**0.975**	**0.972**	**0.978**	**2.74**
Gulshan et al. 2016	**0.961**	**0.952**	**0.970**	**0.42**
Keel et al. 2018	**0.923**	**0.869**	**0.977**	**0.01**
Krause et al. 2018	**0.970**	**0.962**	**0.978**	**0.60**
Li et al. 2019	**0.969**	**0.966**	**0.973**	**2.13**
Li Z et al. 2018	**0.925**	**0.922**	**0.928**	**3.60**
Nagasawa et al. 2019	**0.947**	**0.924**	**0.970**	**0.07**
Raju et al. 2017	**0.803**	**0.799**	**0.806**	**2.59**
Ramachandran et al.	**0.846**	**0.814**	**0.878**	**0.03**
Ramachandran et al.	**0.960**	**0.949**	**0.971**	**0.28**
Raumviboonsuk et al.	**0.968**	**0.966**	**0.970**	**5.08**
Raumviboonsuk et al.	**0.953**	**0.950**	**0.956**	**3.79**
Sandhu et al. 2018	**0.925**	**0.884**	**0.966**	**0.02**
Sayres et al. 2019	**0.915**	**0.903**	**0.927**	**0.23**
Stevenson et al. 201	**0.810**	**0.794**	**0.826**	**0.14**
Ting et al. 2017 (a)	**0.905**	**0.903**	**0.907**	**5.15**
Ting et al. 2017 (b)	**0.987**	**0.985**	**0.989**	**6.55**
Ting et al. 2017 (c)	**0.971**	**0.965**	**0.977**	**0.94**
Ting et al. 2017 (d)	**0.993**	**0.991**	**0.995**	**4.32**
Ting et al. 2017 (e)	**1.000**	**1.000**	**1.000**	**15.40**
Ting et al. 2017 (f)	**0.944**	**0.930**	**0.958**	**0.18**
Ting et al. 2017 (g)	**0.988**	**0.983**	**0.993**	**1.40**
Ting et al. 2017 (h)	**0.989**	**0.985**	**0.993**	**1.74**
Ting et al. 2017 (i)	**0.918**	**0.902**	**0.934**	**0.14**
Ting et al. 2017 (j)	**0.993**	**0.988**	**0.998**	**1.51**
Ting et al. 2017 (k)	**1.000**	**1.000**	**1.000**	**15.40**
Verbraak et al. 2019	**1.000**	**1.000**	**1.000**	**15.40**
Verbraak et al. 2019	**0.794**	**0.772**	**0.816**	**0.07**
Yang et al. 2019	**0.988**	**0.978**	**0.998**	**0.38**
D+L pooled ES	**0.976**	**0.975**	**0.977**	**100.00**

Heterogeneity chi-squared = **30174.23** (d.f. = **39**) p = **0.000**
I-squared (variation in ES attributable to heterogeneity) = **99.9%**
Estimate of between-study variance Tau-squared = **0.0000**

Test of ES=0 : z= **3206.98** p = **0.000**

Supplementary Figure 10 – Pooled specificity for diagnosing features of diabetic retinopathy on retinal fundus photography

Study	ES	[95% Conf.	Interval]	% Weight
Abramoff et al. 2016	**0.870**	**0.854**	**0.886**	**2.64**
Abramoff et al. 2018	**0.907**	**0.887**	**0.927**	**2.59**
Arcadu et al. 2019 (	**0.944**	**0.931**	**0.957**	**2.67**
Arcadu et al. 2019 (	**0.940**	**0.929**	**0.951**	**2.69**
Bellemo et al. 2019	**0.890**	**0.879**	**0.901**	**2.69**
Gargeya et al. 2017	**0.980**	**0.978**	**0.982**	**2.74**
Gargeya et al. 2017	**0.870**	**0.854**	**0.886**	**2.64**
Gargeya et al. 2017	**0.940**	**0.918**	**0.962**	**2.57**
Gulshan et al. 2019	**0.922**	**0.912**	**0.932**	**2.70**
Gulshan et al. 2016	**0.934**	**0.929**	**0.939**	**2.73**
Gulshan et al. 2016	**0.939**	**0.928**	**0.950**	**2.69**
Kanagasingham et al.	**0.920**	**0.893**	**0.947**	**2.49**
Keel et al. 2018	**0.937**	**0.888**	**0.986**	**2.03**
Krause et al. 2018	**0.917**	**0.905**	**0.929**	**2.68**
Li et al. 2019	**0.934**	**0.929**	**0.940**	**2.73**
Li Z et al. 2018	**0.985**	**0.984**	**0.986**	**2.74**
Raju et al. 2017	**0.923**	**0.921**	**0.925**	**2.74**
Ramachandran et al.	**0.797**	**0.761**	**0.833**	**2.31**
Ramachandran et al.	**0.900**	**0.883**	**0.917**	**2.63**
Raumviboonsuk et al.	**0.956**	**0.953**	**0.959**	**2.74**
Raumviboonsuk et al.	**0.982**	**0.980**	**0.984**	**2.74**
Sandhu et al. 2018	**0.950**	**0.916**	**0.984**	**2.35**
Sayres et al. 2019	**0.947**	**0.937**	**0.957**	**2.70**
Stevenson et al. 201	**0.978**	**0.972**	**0.984**	**2.72**
Ting et al. 2017 (a)	**0.916**	**0.914**	**0.918**	**2.74**
Ting et al. 2017 (b)	**0.816**	**0.810**	**0.822**	**2.72**
Ting et al. 2017 (c)	**0.820**	**0.806**	**0.834**	**2.67**
Ting et al. 2017 (d)	**0.733**	**0.720**	**0.746**	**2.67**
Ting et al. 2017 (e)	**0.763**	**0.744**	**0.782**	**2.60**
Ting et al. 2017 (f)	**0.885**	**0.866**	**0.904**	**2.60**
Ting et al. 2017 (g)	**0.865**	**0.850**	**0.880**	**2.65**
Ting et al. 2017 (h)	**0.922**	**0.911**	**0.933**	**2.69**
Ting et al. 2017 (i)	**0.848**	**0.827**	**0.869**	**2.58**
Ting et al. 2017 (j)	**0.831**	**0.810**	**0.852**	**2.58**
Ting et al. 2017 (k)	**0.813**	**0.804**	**0.822**	**2.71**
Verbraak et al. 2019	**0.978**	**0.970**	**0.986**	**2.71**
Verbraak et al. 2019	**0.938**	**0.925**	**0.951**	**2.67**
Yang et al. 2019	**0.880**	**0.852**	**0.908**	**2.45**
D+L pooled ES	**0.902**	**0.889**	**0.916**	**100.00**

Heterogeneity chi-squared = **12048.04** (d.f. = **37**) p = **0.000**
I-squared (variation in ES attributable to heterogeneity) = **99.7%**
Estimate of between-study variance Tau-squared = **0.0018**

Test of ES=0 : z= **127.71** p = **0.000**

Supplementary Figure 11 – Pooled accuracy for diagnosing features of diabetic retinopathy on retinal fundus photography

Study	ES	[95% Conf.	Interval]	% Weight
Choi et al. 2017 (a)	**0.874**	**0.862**	**0.886**	**21.77**
Li et al. 2019	**0.935**	**0.929**	**0.940**	**22.48**
Sandhu et al. 2018	**0.938**	**0.900**	**0.975**	**16.06**
Stevenson et al. 201	**0.950**	**0.941**	**0.959**	**22.15**
Xu et al. 2017	**0.945**	**0.913**	**0.977**	**17.54**
D+L pooled ES	**0.927**	**0.899**	**0.955**	**100.00**

Heterogeneity chi-squared = **108.70** (d.f. = **4**) p = **0.000**
I-squared (variation in ES attributable to heterogeneity) = **96.3%**
Estimate of between-study variance Tau-squared = **0.0009**

Test of ES=0 : z= **65.29** p = **0.000**

Supplementary Figure 12 – Pooled PPV for diagnosing features of diabetic retinopathy on retinal fundus photography

Study	ES	[95% Conf. Interval]		% Weight
Abramoff et al. 2016	**0.674**	**0.652**	**0.696**	**25.03**
Kanagasingham et al.	**0.120**	**0.088**	**0.152**	**24.96**
Verbraak et al. 2019	**0.364**	**0.338**	**0.390**	**25.00**
Verbraak et al. 2019	**0.397**	**0.370**	**0.424**	**25.00**
D+L pooled ES	**0.389**	**0.166**	**0.612**	**100.00**

Heterogeneity chi-squared = **863.79** (d.f. = **3**) p = **0.000**
I-squared (variation in ES attributable to heterogeneity) = **99.7%**
Estimate of between-study variance Tau-squared = **0.0517**

Test of ES=0 : z= **3.41** p = **0.001**

Supplementary Figure 13 – Pooled NPV for diagnosing features of diabetic retinopathy on retinal fundus photography

Study	ES	[95% Conf. Interval]		% Weight
Abramoff et al. 2016	**0.990**	**0.985**	**0.995**	**0.04**
Kanagasingham et al.	**1.000**	**1.000**	**1.000**	**49.08**
Verbraak et al. 2019	**1.000**	**1.000**	**1.000**	**50.84**
Verbraak et al. 2019	**0.989**	**0.983**	**0.995**	**0.03**
D+L pooled ES	**1.000**	**1.000**	**1.000**	**100.00**

Heterogeneity chi-squared = **32.04** (d.f. = **3**) p = **0.000**
I-squared (variation in ES attributable to heterogeneity) = **90.6%**
Estimate of between-study variance Tau-squared = **0.0000**

Test of ES=0 : z= **20142.49** p = **0.000**

Supplementary Figure 14 – Pooled AUC for diagnosing features of age-related macular degeneration on retinal fundus photography

Study	ES	[95% Conf.	Interval]	% Weight
Burlina et al. 2018	**0.960**	**0.957**	**0.963**	**17.11**
Burlina et al. 2018	**0.972**	**0.967**	**0.977**	**16.99**
Keel et al. 2019	**0.967**	**0.966**	**0.968**	**17.23**
Matsuba et al. 2018	**0.998**	**0.988**	**1.007**	**16.29**
Ting et al. 2017 (m)	**0.931**	**0.928**	**0.934**	**17.16**
Yoo et al. 2019 (b)	**0.954**	**0.940**	**0.968**	**15.22**
D+L pooled ES	**0.963**	**0.948**	**0.979**	**100.00**

Heterogeneity chi-squared = **684.77** (d.f. = **5**) p = **0.000**
I-squared (variation in ES attributable to heterogeneity) = **99.3%**
Estimate of between-study variance Tau-squared = **0.0004**

Test of ES=0 : z= **121.07** p = **0.000**

Supplementary Figure 15 – Pooled sensitivity for diagnosing features of age-related macular degeneration on retinal fundus photography

Study	ES	[95% Conf.	Interval]	% Weight
Burlina et al. 2018	**0.884**	**0.879**	**0.889**	**7.52**
Burlina et al. 2018	**0.890**	**0.881**	**0.899**	**3.46**
Keel et al. 2019	**1.000**	**1.000**	**1.000**	**29.59**
Matsuba et al. 2018	**1.000**	**1.000**	**1.000**	**29.58**
Peng et al. 2019	**0.590**	**0.558**	**0.622**	**0.28**
Stevenson et al. 201	**0.991**	**0.987**	**0.995**	**11.22**
Ting et al. 2017 (m)	**0.932**	**0.929**	**0.935**	**17.61**
Yoo et al. 2019 (b)	**0.900**	**0.880**	**0.920**	**0.75**
D+L pooled ES	**0.973**	**0.971**	**0.974**	**100.00**

Heterogeneity chi-squared = **5754.18** (d.f. = **7**) p = **0.000**

I-squared (variation in ES attributable to heterogeneity) = **99.9%**

Estimate of between-study variance Tau-squared = **0.0000**

Test of ES=0 : z= **1111.04** p = **0.000**

Supplementary Figure 16 – Pooled specificity for diagnosing features of age-related macular degeneration on retinal fundus photography

Study	ES	[95% Conf.	Interval]	% Weight
Burlina et al. 2018	**0.941**	**0.937**	**0.945**	**12.93**
Burlina et al. 2018	**0.936**	**0.929**	**0.943**	**12.87**
Keel et al. 2019	**0.962**	**0.961**	**0.963**	**12.96**
Matsuba et al. 2018	**0.973**	**0.943**	**1.003**	**11.27**
Peng et al. 2019	**0.930**	**0.913**	**0.947**	**12.39**
Stevenson et al. 201	**0.889**	**0.876**	**0.902**	**12.59**
Ting et al. 2017 (m)	**0.887**	**0.884**	**0.890**	**12.94**
Yoo et al. 2019 (b)	**0.877**	**0.856**	**0.898**	**12.04**
D+L pooled ES	**0.924**	**0.896**	**0.952**	**100.00**

Heterogeneity chi-squared = **1930.27** (d.f. = **7**) p = **0.000**
I-squared (variation in ES attributable to heterogeneity) = **99.6%**
Estimate of between-study variance Tau-squared = **0.0016**

Test of ES=0 : z= **64.87** p = **0.000**

Supplementary Figure 17 – Pooled accuracy for diagnosing features of age-related macular degeneration on retinal fundus photography

Study	ES	[95% Conf.	Interval]	% Weight
Burlina et al. 2017	**0.794**	**0.783**	**0.805**	**9.10**
Burlina et al. 2018	**0.916**	**0.911**	**0.921**	**9.12**
Burlina et al. 2018	**0.916**	**0.908**	**0.924**	**9.11**
Burlina et al. 2018	**0.757**	**0.747**	**0.767**	**9.10**
Burlina et al. 2018	**0.591**	**0.578**	**0.604**	**9.10**
Choi et al. 2017 (b)	**0.772**	**0.757**	**0.787**	**9.09**
Grassman et al. 2018	**0.633**	**0.624**	**0.642**	**9.11**
Grassman et al. 2018	**0.831**	**0.821**	**0.841**	**9.10**
Peng et al. 2019	**0.671**	**0.640**	**0.702**	**8.99**
Stevenson et al. 201	**0.991**	**0.987**	**0.995**	**9.12**
Yoo et al. 2019 (b)	**0.892**	**0.872**	**0.912**	**9.06**
D+L pooled ES	**0.797**	**0.719**	**0.875**	**100.00**

Heterogeneity chi-squared = **9676.16** (d.f. = **10**) p = **0.000**
I-squared (variation in ES attributable to heterogeneity) = **99.9%**
Estimate of between-study variance Tau-squared = **0.0175**

Test of ES=0 : z= **19.93** p = **0.000**

Supplementary Figure 18 – Pooled AUC for diagnosing features of glaucoma on retinal fundus photography

Study	ES	[95% Conf. Interval]		% Weight
Ahn et al. 2018 (a)	**0.930**	**0.907**	**0.953**	**3.33**
Ahn et al. 2018 (b)	**0.940**	**0.918**	**0.962**	**3.43**
Al-Aswad et al. 2019	**0.926**	**0.877**	**0.975**	**1.91**
Christopher et al. 2	**0.890**	**0.874**	**0.906**	**3.76**
Christopher et al. 2	**0.900**	**0.885**	**0.915**	**3.80**
Christopher et al. 2	**0.910**	**0.895**	**0.925**	**3.83**
Gomez-Valverde et al	**0.940**	**0.919**	**0.961**	**3.47**
Jammal et al. 2019	**0.801**	**0.766**	**0.836**	**2.59**
Lee et al. 2019	**0.939**	**0.906**	**0.972**	**2.72**
Li et al. 2018	**0.986**	**0.983**	**0.989**	**4.23**
Liu et al. 2018 (a)	**0.970**	**0.958**	**0.982**	**3.95**
Liu et al. 2018 (b)	**0.890**	**0.778**	**1.002**	**0.57**
Liu et al. 2019 (a)	**0.996**	**0.995**	**0.997**	**4.25**
Liu et al. 2019 (b)	**0.995**	**0.994**	**0.996**	**4.25**
Liu et al. 2019 (c)	**0.994**	**0.993**	**0.995**	**4.24**
Liu et al. 2019 (d)	**0.987**	**0.985**	**0.989**	**4.24**
Liu et al. 2019 (e)	**0.964**	**0.962**	**0.966**	**4.24**
Liu et al. 2019 (f)	**0.923**	**0.917**	**0.929**	**4.17**
MacCormick et al. 20	**0.997**	**0.988**	**1.006**	**4.07**
MacCormick et al. 20	**0.910**	**0.866**	**0.954**	**2.11**
Medeiros et al. 2019	**0.944**	**0.938**	**0.950**	**4.18**
Phan et al. 2019	**0.978**	**0.968**	**0.988**	**4.04**
Phene et al. 2019 (a	**0.945**	**0.932**	**0.958**	**3.92**
Phene et al. 2019 (b	**0.855**	**0.848**	**0.862**	**4.14**
Phene et al. 2019 ©	**0.881**	**0.847**	**0.915**	**2.67**
Shibata et al. 2018	**0.965**	**0.931**	**0.999**	**2.65**
Shibata et al. 2018	**0.863**	**0.799**	**0.927**	**1.37**
Stevenson et al. 201	**0.700**	**0.681**	**0.719**	**3.61**
Ting et al. 2017 (l)	**0.942**	**0.940**	**0.944**	**4.24**
D+L pooled ES	**0.933**	**0.924**	**0.942**	**100.00**

Heterogeneity chi-squared = **6308.69** (d.f. = **28**) p = **0.000**
I-squared (variation in ES attributable to heterogeneity) = **99.6%**
Estimate of between-study variance Tau-squared = **0.0005**

Test of ES=0 : z= **200.34** p = **0.000**

Supplementary Figure 19 – Pooled sensitivity for diagnosing features of glaucoma on retinal fundus photography

Study	ES	[95% Conf.	Interval]	% Weight
Al-Aswad et al. 2019	0.837	0.768	0.906	3.73
Christopher et al. 2	0.850	0.832	0.868	5.94
Gomez-Valverde et al	0.870	0.840	0.900	5.54
Li et al. 2018	0.956	0.952	0.960	6.20
Liu et al. 2018 (a)	0.879	0.856	0.902	5.78
Liu et al. 2018 (b)	0.867	0.745	0.989	2.02
Liu et al. 2019 (a)	0.962	0.960	0.964	6.22
Liu et al. 2019 (b)	0.961	0.958	0.964	6.21
Liu et al. 2019 (c)	0.960	0.957	0.963	6.21
Liu et al. 2019 (d)	0.936	0.931	0.941	6.20
Liu et al. 2019 (e)	0.910	0.907	0.913	6.21
Liu et al. 2019 (f)	0.877	0.870	0.884	6.17
MacCormick et al. 20	1.000	1.000	1.000	6.22
Medeiros et al. 2019	0.760	0.749	0.771	6.12
Phene et al. 2019 (a	0.800	0.777	0.823	5.80
Rogers et al. 2019	0.809	0.730	0.888	3.30
Stevenson et al. 201	0.603	0.583	0.623	5.89
Ting et al. 2017 (l)	0.964	0.962	0.966	6.22
D+L pooled ES	0.883	0.862	0.904	100.00

Heterogeneity chi-squared = **13162.61** (d.f. = **17**) p = **0.000**
I-squared (variation in ES attributable to heterogeneity) = **99.9%**
Estimate of between-study variance Tau-squared = **0.0019**

Test of ES=0 : z= **82.21** p = **0.000**

Supplementary Figure 20 – Pooled specificity for diagnosing features of glaucoma on retinal fundus photography

Study	ES	[95% Conf. Interval]		% Weight
Al-Aswad et al. 2019	**0.882**	**0.822**	**0.942**	**3.92**
Christopher et al. 2	**0.800**	**0.780**	**0.820**	**5.81**
Gomez-Valverde et al	**0.890**	**0.863**	**0.918**	**5.52**
Li et al. 2018	**0.920**	**0.914**	**0.926**	**6.16**
Liu et al. 2018 (a)	**0.965**	**0.952**	**0.978**	**6.03**
Liu et al. 2018 (b)	**0.867**	**0.745**	**0.989**	**1.84**
Liu et al. 2019 (a)	**0.977**	**0.975**	**0.979**	**6.20**
Liu et al. 2019 (b)	**0.971**	**0.969**	**0.973**	**6.19**
Liu et al. 2019 (c)	**0.961**	**0.958**	**0.964**	**6.19**
Liu et al. 2019 (d)	**0.956**	**0.952**	**0.960**	**6.18**
Liu et al. 2019 (e)	**0.926**	**0.923**	**0.929**	**6.19**
Liu et al. 2019 (f)	**0.808**	**0.799**	**0.817**	**6.12**
MacCormick et al. 20	**0.983**	**0.961**	**1.005**	**5.74**
Medeiros et al. 2019	**0.950**	**0.945**	**0.955**	**6.17**
Phene et al. 2019 (a	**0.902**	**0.885**	**0.919**	**5.93**
Rogers et al. 2019	**0.862**	**0.792**	**0.932**	**3.48**
Stevenson et al. 201	**0.950**	**0.941**	**0.959**	**6.12**
Ting et al. 2017 (l)	**0.872**	**0.869**	**0.875**	**6.19**
D+L pooled ES	**0.918**	**0.898**	**0.938**	**100.00**

Heterogeneity chi-squared = **4898.91** (d.f. = **17**) p = **0.000**
I-squared (variation in ES attributable to heterogeneity) = **99.7%**
Estimate of between-study variance Tau-squared = **0.0016**

Test of ES=0 : z= **91.47** p = **0.000**

Supplementary Figure 21 – Pooled accuracy for diagnosing features of glaucoma on retinal fundus photography

Study	ES	[95% Conf.	Interval]	% Weight
Rogers et al. 2019	**0.834**	**0.759**	**0.909**	**8.26**
Medeiros et al. 2019	**0.837**	**0.828**	**0.846**	**13.71**
Ahn et al. 2018 (a)	**0.845**	**0.812**	**0.878**	**12.26**
Ahn et al. 2018 (b)	**0.879**	**0.849**	**0.909**	**12.53**
Gomez-Valverde et al	**0.881**	**0.852**	**0.909**	**12.61**
Stevenson et al. 201	**0.908**	**0.896**	**0.920**	**13.62**
Liu et al. 2018	**0.916**	**0.896**	**0.936**	**13.23**
Li et al. 2018	**0.929**	**0.923**	**0.935**	**13.79**
D+L pooled ES	**0.881**	**0.847**	**0.915**	**100.00**

Heterogeneity chi-squared = **305.24** (d.f. = **7**) p = **0.000**

I-squared (variation in ES attributable to heterogeneity) = **97.7%**

Estimate of between-study variance Tau-squared = **0.0022**

Test of ES=0 : z= **50.77** p = **0.000**

Supplementary Figure 22 – Pooled sensitivity for diagnosing features of plus disease in retinopathy of prematurity on retinal fundus photography

Study	ES	[95% Conf. Interval]		% Weight
Brown et al. 2018	**1.000**	**1.000**	**1.000**	**33.60**
Redd et al. 2018	**0.940**	**0.933**	**0.947**	**33.39**
Zhang et al. 2019	**0.941**	**0.930**	**0.952**	**33.01**
D+L pooled ES	**0.960**	**0.913**	**1.008**	**100.00**

Heterogeneity chi-squared = **419.45** (d.f. = **2**) p = **0.000**
I-squared (variation in ES attributable to heterogeneity) = **99.5%**
Estimate of between-study variance Tau-squared = **0.0018**

Test of ES=0 : z= **39.32** p = **0.000**

Supplementary Figure 23 – Pooled specificity for diagnosing features of plus disease in retinopathy of prematurity on retinal fundus photography

Study	ES	[95% Conf. Interval]		% Weight
Brown et al. 2018	**0.940**	**0.893**	**0.987**	**32.72**
Redd et al. 2018	**0.790**	**0.779**	**0.801**	**33.61**
Zhang et al. 2019	**0.993**	**0.989**	**0.997**	**33.67**
D+L pooled ES	**0.907**	**0.749**	**1.066**	**100.00**

Heterogeneity chi-squared = **1082.84** (d.f. = **2**) p = **0.000**
I-squared (variation in ES attributable to heterogeneity) = **99.8%**
Estimate of between-study variance Tau-squared = **0.0194**

Test of ES=0 : z= **11.22** p = **0.000**

Supplementary Figure 24 – Pooled AUC for diagnosing features of diabetic retinopathy on OCT scans

Study	ES	[95% Conf.	Interval]	% Weight
Alqudah et al. 2019	**0.999**	**0.998**	**1.001**	**6.67**
Bhatia et al. 2019 (	**0.980**	**0.957**	**1.003**	**0.02**
Bhatia et al. 2019 (	**0.998**	**0.989**	**1.007**	**0.16**
Karri et al. 2017 (a	**0.860**	**0.712**	**1.008**	**0.00**
Kermany et al. 2018	**0.999**	**0.997**	**1.001**	**2.93**
Kermany et al. 2018	**1.000**	**1.000**	**1.000**	**44.35**
Kermany et al. 2018	**0.987**	**0.977**	**0.997**	**0.12**
Li et al. 2019	**1.000**	**1.000**	**1.000**	**44.41**
Li et al. 2019	**0.996**	**0.993**	**0.999**	**1.25**
Perdomo et al. 2019	**0.860**	**0.847**	**0.873**	**0.07**
D+L pooled ES	**1.000**	**0.999**	**1.000**	**100.00**

Heterogeneity chi-squared = **479.83** (d.f. = **9**) p = **0.000**
I-squared (variation in ES attributable to heterogeneity) = **98.1%**
Estimate of between-study variance Tau-squared = **0.0000**

Test of ES=0 : z= **5643.56** p = **0.000**

Supplementary Figure 25 – Pooled sensitivity for diagnosing features of diabetic retinopathy on OCT scans

Study	ES	[95% Conf.	Interval]	% Weight
Alqudah et al. 2019	**0.960**	**0.949**	**0.971**	**8.63**
Bhatia et al. 2019 (	**0.960**	**0.928**	**0.992**	**6.90**
Bhatia et al. 2019 (	**0.960**	**0.922**	**0.998**	**6.22**
Chan et al. 2018	**0.938**	**0.930**	**0.945**	**8.78**
Das et al. 2019	**0.996**	**0.992**	**1.000**	**8.89**
ElTanboly et al. 201	**0.830**	**0.617**	**1.043**	**0.62**
Kermany et al. 2018	**0.978**	**0.969**	**0.987**	**8.71**
Kermany et al. 2018	**1.000**	**1.000**	**1.000**	**8.93**
Kermany et al. 2018	**0.968**	**0.953**	**0.983**	**8.34**
Li et al. 2019	**0.900**	**0.880**	**0.920**	**7.98**
Li et al. 2019	**0.978**	**0.969**	**0.987**	**8.71**
Li et al. 2019	**0.986**	**0.980**	**0.992**	**8.84**
Perdomo et al. 2019	**0.830**	**0.816**	**0.844**	**8.45**
D+L pooled ES	**0.954**	**0.937**	**0.972**	**100.00**

Heterogeneity chi-squared = **1098.85** (d.f. = **12**) p = **0.000**
I-squared (variation in ES attributable to heterogeneity) = **98.9%**
Estimate of between-study variance Tau-squared = **0.0009**

Test of ES=0 : z= **107.63** p = **0.000**

Supplementary Figure 26– Pooled specificity for diagnosing features of diabetic retinopathy on OCT scans

Study	ES	[95% Conf.	Interval]	% Weight
Bhatia et al. 2019 (	0.910	0.864	0.956	0.11
Bhatia et al. 2019 (	0.960	0.922	0.998	0.17
Chan et al. 2018	0.938	0.930	0.945	3.81
Das et al. 2019	0.999	0.996	1.001	16.90
ElTanboly et al. 201	1.000	1.000	1.000	25.65
Kermany et al. 2018	0.974	0.964	0.984	2.30
Kermany et al. 2018	1.000	1.000	1.000	25.73
Kermany et al. 2018	0.996	0.990	1.002	6.11
Li et al. 2019	0.950	0.935	0.965	1.11
Li et al. 2019	0.994	0.989	0.999	7.57
Li et al. 2019	0.991	0.986	0.996	8.05
Perdomo et al. 2019	0.930	0.921	0.939	2.50
D+L pooled ES	0.993	0.991	0.994	100.00

Heterogeneity chi-squared = **599.89** (d.f. = **11**) p = **0.000**
I-squared (variation in ES attributable to heterogeneity) = **98.2%**
Estimate of between-study variance Tau-squared = **0.0000**

Test of ES=0 : z= **1242.06** p = **0.000**

Supplementary Figure 27 – Pooled accuracy for diagnosing features of diabetic retinopathy on OCT scans

Study	ES	[95% Conf. Interval]		% Weight
Alqudah et al. 2019	**0.992**	**0.987**	**0.997**	**9.10**
Bhatia et al. 2019 (	**0.930**	**0.889**	**0.971**	**3.92**
Bhatia et al. 2019 (	**0.960**	**0.922**	**0.998**	**4.23**
Chan et al. 2018	**0.938**	**0.930**	**0.945**	**8.88**
Das et al. 2019	**0.996**	**0.992**	**1.000**	**9.16**
De Fauw et al. 2018	**0.945**	**0.931**	**0.959**	**7.98**
De Fauw et al. 2018	**0.966**	**0.933**	**0.999**	**4.93**
ElTanboly et al. 201	**0.920**	**0.767**	**1.073**	**0.46**
Kermany et al. 2018	**0.966**	**0.955**	**0.977**	**8.42**
Kermany et al. 2018	**1.000**	**1.000**	**1.000**	**9.28**
Kermany et al. 2018	**0.982**	**0.970**	**0.994**	**8.36**
Li et al. 2019	**0.920**	**0.902**	**0.938**	**7.32**
Li et al. 2019	**0.986**	**0.979**	**0.993**	**8.89**
Li et al. 2019	**0.988**	**0.983**	**0.993**	**9.07**
D+L pooled ES	**0.970**	**0.959**	**0.981**	**100.00**

Heterogeneity chi-squared = **518.70** (d.f. = **13**) p = **0.000**
I-squared (variation in ES attributable to heterogeneity) = **97.5%**
Estimate of between-study variance Tau-squared = **0.0003**

Test of ES=0 : z= **177.65** p = **0.000**

Supplementary Figure 28 – Pooled AUC for diagnosing features of age-related macular degeneration on OCT scans

Study	ES	[95% Conf.	Interval]	% Weight
Alqudah et al. 2019	1.000	1.000	1.000	10.06
Bhatia et al. 2019 (	0.890	0.859	0.921	6.76
Bhatia et al. 2019 (	0.990	0.974	1.006	8.92
Bhatia et al. 2019 (	0.990	0.970	1.010	8.45
Bhatia et al. 2019 (	0.980	0.956	1.004	7.87
Bhatia et al. 2019 (	0.990	0.973	1.007	8.82
Karri et al. 2017 (b	0.890	0.756	1.024	1.01
Kermany et al. 2018	1.000	0.998	1.001	10.04
Lee et al. 2017	0.928	0.924	0.932	10.00
Prahs et al. 2017	0.968	0.963	0.973	9.95
Treder et al. 2017	0.997	0.986	1.008	9.51
Yoo et al. 2019 (a)	0.914	0.896	0.932	8.62
D+L pooled ES	0.969	0.955	0.983	100.00

Heterogeneity chi-squared = **1918.19** (d.f. = **11**) p = **0.000**
I-squared (variation in ES attributable to heterogeneity) = **99.4%**
Estimate of between-study variance Tau-squared = **0.0005**

Test of ES=0 : z= **133.73** p = **0.000**

Supplementary Figure 29 – Pooled sensitivity for diagnosing features of age-related macular degeneration on OCT scans

Study	ES	[95% Conf.	Interval]	% Weight
Alqudah et al. 2019	**1.000**	**1.000**	**1.000**	**19.29**
Bhatia et al. 2019 (	**0.930**	**0.904**	**0.956**	**0.04**
Bhatia et al. 2019 (	**0.960**	**0.928**	**0.992**	**0.03**
Bhatia et al. 2019 (	**1.000**	**1.000**	**1.000**	**19.24**
Bhatia et al. 2019 (	**0.990**	**0.973**	**1.007**	**0.09**
Bhatia et al. 2019 (	**0.950**	**0.913**	**0.987**	**0.02**
Hwang et al. 2019 (a	**0.872**	**0.861**	**0.882**	**0.24**
Hwang et al. 2019 (b	**0.865**	**0.854**	**0.875**	**0.22**
Hwang et al. 2019 (c	**0.885**	**0.875**	**0.895**	**0.26**
Hwang et al. 2019 (d	**0.980**	**0.970**	**0.990**	**0.26**
Hwang et al. 2019 (e	**0.992**	**0.986**	**0.998**	**0.63**
Hwang et al. 2019 (f	**1.000**	**1.000**	**1.000**	**19.28**
Kermany et al. 2018	**0.980**	**0.968**	**0.992**	**0.17**
Lee et al. 2017	**0.846**	**0.841**	**0.851**	**1.03**
Motozawa et al. 2019	**1.000**	**1.000**	**1.000**	**19.28**
Prahs et al. 2017	**0.942**	**0.936**	**0.948**	**0.65**
Treder et al. 2017	**1.000**	**1.000**	**1.000**	**19.24**
Yoo et al. 2019 (a)	**0.808**	**0.782**	**0.834**	**0.04**
D+L pooled ES	**0.997**	**0.996**	**0.997**	**100.00**

Heterogeneity chi-squared = **6047.40** (d.f. = **17**) p = **0.000**
I-squared (variation in ES attributable to heterogeneity) = **99.7%**
Estimate of between-study variance Tau-squared = **0.0000**

Test of ES=0 : z= **3804.88** p = **0.000**

Supplementary Figure 30 – Pooled specificity for diagnosing features of age-related macular degeneration on OCT scans

Study	ES	[95% Conf.	Interval]	% Weight
Bhatia et al. 2019 (	**0.790**	**0.749**	**0.831**	**5.08**
Bhatia et al. 2019 (	**0.950**	**0.915**	**0.985**	**5.44**
Bhatia et al. 2019 (	**0.920**	**0.867**	**0.973**	**4.30**
Bhatia et al. 2019 (	**0.640**	**0.559**	**0.721**	**2.88**
Bhatia et al. 2019 (	**0.940**	**0.900**	**0.980**	**5.13**
Hwang et al. 2019 (a	**0.978**	**0.974**	**0.983**	**6.83**
Hwang et al. 2019 (b	**0.990**	**0.987**	**0.994**	**6.85**
Hwang et al. 2019 (c	**0.990**	**0.987**	**0.993**	**6.85**
Hwang et al. 2019 (d	**0.972**	**0.960**	**0.984**	**6.67**
Hwang et al. 2019 (e	**0.916**	**0.896**	**0.936**	**6.34**
Hwang et al. 2019 (f	**0.980**	**0.970**	**0.990**	**6.72**
Kermany et al. 2018	**0.992**	**0.984**	**1.000**	**6.78**
Lee et al. 2017	**0.915**	**0.911**	**0.919**	**6.84**
Motozawa et al. 2019	**0.918**	**0.890**	**0.946**	**5.92**
Prahs et al. 2017	**0.941**	**0.935**	**0.947**	**6.81**
Treder et al. 2017	**0.920**	**0.867**	**0.973**	**4.30**
Yoo et al. 2019 (a)	**0.883**	**0.862**	**0.904**	**6.28**
D+L pooled ES	**0.932**	**0.914**	**0.950**	**100.00**

Heterogeneity chi-squared = **1513.56** (d.f. = **16**) p = **0.000**
I-squared (variation in ES attributable to heterogeneity) = **98.9%**
Estimate of between-study variance Tau-squared = **0.0012**

Test of ES=0 : z= **101.40** p = **0.000**

Supplementary Figure 31 – Pooled accuracy for diagnosing features of age-related macular degeneration on OCT scans

Study	ES	[95% Conf.	Interval]	% Weight
Alqudah et al. 2019	**1.000**	**1.000**	**1.000**	**5.47**
Bhatia et al. 2019 (	**0.890**	**0.859**	**0.921**	**5.16**
Bhatia et al. 2019 (	**0.950**	**0.915**	**0.985**	**5.08**
Bhatia et al. 2019 (	**0.960**	**0.922**	**0.998**	**5.01**
Bhatia et al. 2019 (	**0.870**	**0.813**	**0.927**	**4.56**
Bhatia et al. 2019 (	**0.950**	**0.913**	**0.987**	**5.05**
De Fauw et al. 2018	**0.945**	**0.931**	**0.959**	**5.40**
De Fauw et al. 2018	**0.966**	**0.933**	**0.999**	**5.13**
Hwang et al. 2019 (a	**0.907**	**0.898**	**0.916**	**5.44**
Hwang et al. 2019 (b	**0.914**	**0.905**	**0.923**	**5.45**
Hwang et al. 2019 (c	**0.927**	**0.918**	**0.935**	**5.45**
Hwang et al. 2019 (d	**0.959**	**0.944**	**0.973**	**5.40**
Hwang et al. 2019 (e	**0.912**	**0.892**	**0.932**	**5.34**
Hwang et al. 2019 (f	**0.969**	**0.957**	**0.982**	**5.42**
Kermany et al. 2018	**0.990**	**0.981**	**0.999**	**5.45**
Lee et al. 2017	**0.876**	**0.872**	**0.880**	**5.47**
Motozawa et al. 2019	**0.990**	**0.980**	**1.000**	**5.44**
Treder et al. 2017	**0.960**	**0.922**	**0.998**	**5.01**
Yoo et al. 2019 (a)	**0.833**	**0.809**	**0.857**	**5.28**
D+L pooled ES	**0.936**	**0.906**	**0.965**	**100.00**

Heterogeneity chi-squared = **4454.61** (d.f. = **18**) p = **0.000**
I-squared (variation in ES attributable to heterogeneity) = **99.6%**
Estimate of between-study variance Tau-squared = **0.0042**

Test of ES=0 : z= **61.81** p = **0.000**

Supplementary Figure 32 – Pooled AUC for diagnosing features of glaucoma on OCT scans

Study	ES	[95% Conf.	Interval]	% Weight
Asaoka et al. 2019	**0.937**	**0.903**	**0.971**	**15.52**
Asaoka et al. 2019 (	**0.948**	**0.918**	**0.978**	**16.70**
Asaoka et al. 2019 (	**0.994**	**0.982**	**1.006**	**22.48**
Maetshke et al. 2019	**0.940**	**0.896**	**0.984**	**12.45**
Muhammad et al. 2017	**0.945**	**0.901**	**0.989**	**12.49**
Zheng et al. 2019	**0.990**	**0.971**	**1.009**	**20.35**
D+L pooled ES	**0.964**	**0.941**	**0.986**	**100.00**

Heterogeneity chi-squared = **22.44** (d.f. = **5**) p = **0.000**
I-squared (variation in ES attributable to heterogeneity) = **77.7%**
Estimate of between-study variance Tau-squared = **0.0006**

Test of ES=0 : z= **83.44** p = **0.000**

Supplementary Figure 33 – Pooled AUC for diagnosing lung nodules on CT scans

Study	ES	[95% Conf.	Interval]	% Weight
Al-Shabi et al. 2019	0.956	0.942	0.970	3.32
Causey et al. 2018 (	0.984	0.972	0.996	3.35
Causey et al. 2018 (	0.974	0.953	0.995	3.15
Chae et al. 2019	0.850	0.760	0.940	1.28
Cheng et al. 2016 (b	0.984	0.977	0.991	3.42
da Silva et al. 2017	0.949	0.919	0.979	2.89
da Silva et al. 2018	0.955	0.946	0.964	3.40
Dai et al. 2018	0.969	0.946	0.992	3.10
Gruetzemacher et al.	0.932	0.918	0.947	3.31
Liu et al. 2017	0.732	0.684	0.780	2.33
Monkam et al. 2018	0.870	0.857	0.883	3.34
Nibali et al. 2017	0.946	0.911	0.980	2.77
Onishi et al. 2019	0.841	0.748	0.934	1.24
Paul et al. 2018	0.940	0.910	0.970	2.90
Sahu et al. 2019	0.980	0.956	1.004	3.08
Setio et al. 2016 (a	0.969	0.959	0.979	3.38
Shaffie et al. 2018	0.957	0.943	0.972	3.30
Shen et al. 2017	0.930	0.917	0.943	3.33
Song et al. 2017 (a)	0.910	0.902	0.918	3.41
Tan et al. 2019	0.960	0.937	0.983	3.11
Tran et al. 2019	0.982	0.974	0.990	3.41
Uthoff et al. 2019	0.965	0.929	1.001	2.72
Xie et al. 2018	0.966	0.958	0.974	3.41
Xie et al. 2019	0.957	0.948	0.966	3.40
Zhang et al. 2019	0.710	0.601	0.819	1.00
Zhang et al. 2019	0.969	0.958	0.979	3.37
Zhang S et al. 2019	0.994	0.989	0.999	3.43
Zhang T et al. 2017	0.932	0.920	0.944	3.35
Zhao X et al. 2018	0.877	0.853	0.901	3.09
Zhao X et al. 2019 (	0.910	0.897	0.922	3.34
Zhao X et al. 2019 (	0.940	0.930	0.950	3.38
Zhao X et al. 2019 (	0.910	0.898	0.923	3.35
Zhao X et al. 2019 (	0.882	0.868	0.896	3.32
D+L pooled ES	0.937	0.924	0.949	100.00

Heterogeneity chi-squared = **1066.67** (d.f. = **32**) p = **0.000**
I-squared (variation in ES attributable to heterogeneity) = **97.0%**
Estimate of between-study variance Tau-squared = **0.0012**

Test of ES=0 : z= **142.58** p = **0.000**

Supplementary Figure 34 – Pooled sensitivity for diagnosing lung nodules on CT scans

Study	ES	[95% Conf. Interval]		% Weight
Al-Shabi et al. 2019	**0.887**	**0.865**	**0.908**	**2.05**
Ali et al. 2018	**0.589**	**0.552**	**0.626**	**2.01**
Causey et al. 2018 (	**0.948**	**0.927**	**0.969**	**2.05**
Causey et al. 2018 (	**0.885**	**0.842**	**0.928**	**1.99**
Cheng et al. 2016 (b	**0.908**	**0.893**	**0.923**	**2.07**
Ciompi et al. 2017 (	**0.822**	**0.792**	**0.852**	**2.03**
Ciompi et al. 2017 (	**0.828**	**0.799**	**0.857**	**2.04**
Ciompi et al. 2017 (	**0.649**	**0.612**	**0.686**	**2.01**
Ciompi et al. 2017 (	**0.874**	**0.848**	**0.900**	**2.04**
Ciompi et al. 2017 (	**0.604**	**0.566**	**0.642**	**2.01**
Ciompi et al. 2017 (	**0.643**	**0.606**	**0.680**	**2.01**
da Silva et al. 2017	**0.947**	**0.915**	**0.978**	**2.03**
da Silva et al. 2018	**0.922**	**0.910**	**0.934**	**2.07**
Dai et al. 2018	**0.913**	**0.874**	**0.951**	**2.01**
Gruetzemacher et al.	**0.893**	**0.875**	**0.910**	**2.06**
Hamidian et al. 2017	**0.800**	**0.723**	**0.877**	**1.82**
Hua et al. 2015 (a)	**0.733**	**0.716**	**0.750**	**2.06**
Hua et al. 2015 (b)	**0.734**	**0.717**	**0.751**	**2.06**
Jiang et al. 2018	**0.801**	**0.796**	**0.805**	**2.08**
Kang et al. 2017	**0.984**	**0.976**	**0.993**	**2.07**
Li et al. 2016	**0.890**	**0.884**	**0.896**	**2.07**
Li et al. 2019	**0.862**	**0.838**	**0.886**	**2.05**
Liu et al. 2019	**0.873**	**0.845**	**0.901**	**2.04**
Monkam et al. 2018	**0.838**	**0.824**	**0.852**	**2.07**
Naqi et al. 2018	**0.956**	**0.942**	**0.970**	**2.07**
Nasrullah et al. 201	**0.940**	**0.930**	**0.949**	**2.07**
Nibali et al. 2017	**0.911**	**0.867**	**0.954**	**1.99**
Onishi et al. 2019	**0.978**	**0.941**	**1.015**	**2.01**
Ren et al. 2019	**0.810**	**0.732**	**0.888**	**1.82**
Sahu et al. 2019	**0.894**	**0.841**	**0.947**	**1.95**
Setio et al. 2016 (a	**0.901**	**0.884**	**0.918**	**2.06**
Setio et al. 2016 (c	**0.765**	**0.737**	**0.793**	**2.04**
Shaffie et al. 2018	**0.850**	**0.824**	**0.876**	**2.04**
Shen et al. 2017	**0.770**	**0.748**	**0.792**	**2.05**
Song et al. 2017 (a)	**0.840**	**0.829**	**0.850**	**2.07**
Song et al. 2017 (b)	**0.807**	**0.796**	**0.818**	**2.07**
Song et al. 2017 (c)	**0.840**	**0.829**	**0.850**	**2.07**
Tran et al. 2019	**0.960**	**0.949**	**0.971**	**2.07**
Tu et al. 2017 (a)	**0.961**	**0.876**	**1.046**	**1.77**
Tu et al. 2017 (b)	**0.869**	**0.721**	**1.017**	**1.37**
Tu et al. 2017 (c)	**0.921**	**0.803**	**1.039**	**1.56**
Uthoff et al. 2019	**1.000**	**1.000**	**1.000**	**2.08**
Xie et al. 2018	**0.842**	**0.826**	**0.858**	**2.06**
Xie et al. 2019	**0.865**	**0.850**	**0.880**	**2.07**
Ye et al. 2019 (a)	**0.950**	**0.926**	**0.974**	**2.05**
Zhang C et al. 2019	**0.960**	**0.906**	**1.014**	**1.94**
Zhang et al. 2019	**0.800**	**0.704**	**0.896**	**1.70**
Zhang S et al. 2019	**0.974**	**0.962**	**0.985**	**2.07**
Zhang T et al. 2017	**0.935**	**0.923**	**0.947**	**2.07**
Zhao X et al. 2019 (	**0.894**	**0.881**	**0.908**	**2.07**
D+L pooled ES	**0.860**	**0.831**	**0.890**	**100.00**

Heterogeneity chi-squared = **18153.44** (d.f. = **49**) p = **0.000**
I-squared (variation in ES attributable to heterogeneity) = **99.7%**
Estimate of between-study variance Tau-squared = **0.0109**

Test of ES=0 : z= **57.09** p = **0.000**

Supplementary Figure 35 – Pooled specificity for diagnosing lung nodules on CT scans

Study	ES	[95% Conf.	Interval]	% Weight
Ali et al. 2018	**0.553**	**0.515**	**0.591**	**3.30**
Causey et al. 2018 (	**0.943**	**0.921**	**0.965**	**3.47**
Causey et al. 2018 (	**0.942**	**0.911**	**0.973**	**3.37**
Cheng et al. 2016 (b	**0.981**	**0.974**	**0.988**	**3.55**
da Silva et al. 2017	**0.951**	**0.922**	**0.981**	**3.39**
da Silva et al. 2018	**0.986**	**0.981**	**0.991**	**3.55**
Dai et al. 2018	**0.917**	**0.879**	**0.954**	**3.30**
Hua et al. 2015 (a)	**0.787**	**0.771**	**0.803**	**3.51**
Hua et al. 2015 (b)	**0.822**	**0.807**	**0.837**	**3.52**
Jung et al. 2018	**0.993**	**0.988**	**0.998**	**3.56**
Kang et al. 2017	**0.898**	**0.876**	**0.919**	**3.47**
Naqi et al. 2018	**0.970**	**0.958**	**0.982**	**3.53**
Nasrullah et al. 201	**0.898**	**0.886**	**0.910**	**3.53**
Nibali et al. 2017	**0.884**	**0.835**	**0.933**	**3.14**
Onishi et al. 2019	**0.778**	**0.673**	**0.883**	**2.20**
Ren et al. 2019	**0.950**	**0.907**	**0.993**	**3.23**
Sahu et al. 2019	**0.956**	**0.921**	**0.991**	**3.33**
Shaffie et al. 2018	**0.959**	**0.944**	**0.973**	**3.52**
Shen et al. 2017	**0.930**	**0.917**	**0.943**	**3.52**
Song et al. 2017 (a)	**0.843**	**0.833**	**0.853**	**3.54**
Song et al. 2017 (b)	**0.839**	**0.829**	**0.849**	**3.54**
Song et al. 2017 (c)	**0.813**	**0.803**	**0.824**	**3.54**
Tran et al. 2019	**0.973**	**0.964**	**0.982**	**3.54**
Uthoff et al. 2019	**0.960**	**0.922**	**0.998**	**3.29**
Xie et al. 2018	**0.920**	**0.908**	**0.932**	**3.53**
Xie et al. 2019	**0.940**	**0.929**	**0.951**	**3.54**
Zhang C et al. 2019	**0.880**	**0.790**	**0.970**	**2.45**
Zhang et al. 2019	**0.530**	**0.410**	**0.650**	**1.98**
Zhang S et al. 2019	**0.963**	**0.949**	**0.976**	**3.52**
Zhang T et al. 2017	**0.902**	**0.888**	**0.916**	**3.52**
D+L pooled ES	**0.896**	**0.871**	**0.921**	**100.00**

Heterogeneity chi-squared = **3448.11** (d.f. = **29**) p = **0.000**

I-squared (variation in ES attributable to heterogeneity) = **99.2%**

Estimate of between-study variance Tau-squared = **0.0047**

Test of ES=0 : z= **69.50** p = **0.000**

Supplementary Figure 36 – Pooled accuracy for diagnosing lung nodules on CT scans

Study	ES	[95% Conf.	Interval]	% Weight
Al-Shabi et al. 2019	**0.885**	**0.863**	**0.906**	**2.77**
Ali et al. 2018	**0.644**	**0.608**	**0.680**	**2.60**
Causey et al. 2018 (	**0.946**	**0.924**	**0.968**	**2.77**
Causey et al. 2018 (	**0.913**	**0.875**	**0.951**	**2.58**
Chen et al. 2019	**0.901**	**0.891**	**0.911**	**2.86**
Cheng et al. 2016 (b	**0.944**	**0.932**	**0.956**	**2.84**
Ciompi et al. 2017 (	**0.729**	**0.695**	**0.763**	**2.63**
da Silva et al. 2017	**0.948**	**0.917**	**0.979**	**2.67**
da Silva et al. 2018	**0.976**	**0.970**	**0.983**	**2.87**
Dai et al. 2018	**0.915**	**0.877**	**0.952**	**2.58**
Li et al. 2016	**0.864**	**0.857**	**0.871**	**2.87**
Naqi et al. 2018	**0.969**	**0.957**	**0.981**	**2.84**
Nasrullah et al. 201	**0.888**	**0.876**	**0.900**	**2.84**
Nibali et al. 2017	**0.899**	**0.853**	**0.945**	**2.46**
Nishio et al. 2018	**0.680**	**0.598**	**0.762**	**1.86**
Paul et al. 2018	**0.869**	**0.826**	**0.912**	**2.51**
Ren et al. 2019	**0.900**	**0.841**	**0.959**	**2.24**
Sahu et al. 2019	**0.932**	**0.888**	**0.975**	**2.50**
Shaffie et al. 2018	**0.912**	**0.891**	**0.933**	**2.78**
Shen et al. 2017	**0.871**	**0.854**	**0.889**	**2.81**
Song et al. 2017 (a)	**0.841**	**0.831**	**0.852**	**2.85**
Song et al. 2017 (b)	**0.824**	**0.813**	**0.834**	**2.85**
Song et al. 2017 (c)	**0.826**	**0.815**	**0.836**	**2.85**
Tan et al. 2019	**0.895**	**0.859**	**0.930**	**2.61**
Tran et al. 2019	**0.972**	**0.963**	**0.981**	**2.86**
Tu et al. 2017 (c)	**0.917**	**0.796**	**1.038**	**1.33**
Uthoff et al. 2019	**0.980**	**0.953**	**1.007**	**2.71**
Xie et al. 2018	**0.895**	**0.882**	**0.909**	**2.84**
Xie et al. 2019	**0.916**	**0.904**	**0.928**	**2.84**
Zhang C et al. 2019	**0.920**	**0.845**	**0.995**	**1.98**
Zhang et al. 2019	**0.780**	**0.681**	**0.879**	**1.61**
Zhang et al. 2019	**0.938**	**0.923**	**0.953**	**2.83**
Zhang T et al. 2017	**0.950**	**0.940**	**0.960**	**2.85**
Zhao X et al. 2018	**0.822**	**0.794**	**0.850**	**2.71**
Zhao X et al. 2019 (	**0.923**	**0.911**	**0.934**	**2.85**
Zhao X et al. 2019 (	**0.943**	**0.933**	**0.953**	**2.85**
Zhao X et al. 2019 (	**0.938**	**0.928**	**0.949**	**2.85**
Zhao X et al. 2019 (	**0.817**	**0.800**	**0.834**	**2.81**
D+L pooled ES	**0.889**	**0.870**	**0.908**	**100.00**

Heterogeneity chi-squared = **2248.96** (d.f. = **37**) p = **0.000**
I-squared (variation in ES attributable to heterogeneity) = **98.4%**
Estimate of between-study variance Tau-squared = **0.0033**

Test of ES=0 : z= **91.93** p = **0.000**

Supplementary Figure 37 – Pooled PPV for diagnosing lung nodules on CT scans

Study	ES	[95% Conf.	Interval]	% Weight
Al-Shabi et al. 2019	**0.874**	**0.851**	**0.896**	**6.04**
Ali et al. 2018	**0.542**	**0.504**	**0.580**	**5.97**
Cheng et al. 2016 (b	**0.916**	**0.901**	**0.931**	**6.06**
Ciompi et al. 2017 (	**0.892**	**0.868**	**0.916**	**6.03**
Ciompi et al. 2017 (	**0.889**	**0.865**	**0.913**	**6.03**
Ciompi et al. 2017 (	**0.436**	**0.398**	**0.474**	**5.97**
Ciompi et al. 2017 (	**0.874**	**0.848**	**0.900**	**6.02**
Ciompi et al. 2017 (	**0.784**	**0.752**	**0.816**	**6.00**
Ciompi et al. 2017 (	**0.327**	**0.291**	**0.363**	**5.98**
Li et al. 2019	**0.570**	**0.536**	**0.604**	**5.99**
Liu et al. 2019	**0.797**	**0.763**	**0.831**	**5.99**
Nibali et al. 2017	**0.893**	**0.847**	**0.940**	**5.92**
Shaffie et al. 2018	**0.940**	**0.923**	**0.957**	**6.05**
Tu et al. 2017 (a)	**0.996**	**0.968**	**1.024**	**6.02**
Tu et al. 2017 (b)	**0.880**	**0.738**	**1.022**	**4.94**
Tu et al. 2017 (c)	**0.878**	**0.735**	**1.021**	**4.93**
Xie et al. 2019	**0.877**	**0.863**	**0.892**	**6.06**
D+L pooled ES	**0.785**	**0.711**	**0.858**	**100.00**

Heterogeneity chi-squared = **2117.43** (d.f. = **16**) p = **0.000**
I-squared (variation in ES attributable to heterogeneity) = **99.2%**
Estimate of between-study variance Tau-squared = **0.0231**

Test of ES=0 : z= **20.94** p = **0.000**

Supplementary Figure 38 – Pooled F1 score for diagnosing lung nodules on CT scans

Study	ES	[95% Conf. Interval]		% Weight
Ciompi et al. 2017 (	**0.633**	**0.596**	**0.670**	**10.71**
Ciompi et al. 2017 (	**0.892**	**0.868**	**0.916**	**11.23**
Ciompi et al. 2017 (	**0.647**	**0.610**	**0.684**	**10.72**
Ciompi et al. 2017 (	**0.773**	**0.741**	**0.805**	**10.92**
Ciompi et al. 2017 (	**0.779**	**0.747**	**0.811**	**10.93**
Ciompi et al. 2017 (	**0.792**	**0.761**	**0.823**	**10.96**
Li et al. 2016	**0.877**	**0.870**	**0.884**	**11.59**
Monkam et al. 2018	**0.830**	**0.816**	**0.844**	**11.48**
Xie et al. 2019	**0.871**	**0.856**	**0.886**	**11.47**
D+L pooled ES	**0.790**	**0.747**	**0.834**	**100.00**

Heterogeneity chi-squared = **383.12** (d.f. = **8**) p = **0.000**
I-squared (variation in ES attributable to heterogeneity) = **97.9%**
Estimate of between-study variance Tau-squared = **0.0042**

Test of ES=0 : z= **35.61** p = **0.000**

Supplementary Figure 39 – Pooled AUC for diagnosing lung cancer on CT scans

Study	ES	[95% Conf.	Interval]	% Weight
Alakwaa et al. 2017	**0.830**	**0.794**	**0.866**	**24.51**
Ardila et al. 2019 (	**0.944**	**0.939**	**0.949**	**30.26**
Ardila et al. 2019 (	**0.955**	**0.943**	**0.967**	**29.63**
Beig et al. 2019	**0.740**	**0.669**	**0.811**	**15.60**
D+L pooled ES	**0.887**	**0.847**	**0.928**	**100.00**

Heterogeneity chi-squared = **72.91** (d.f. = **3**) p = **0.000**
I-squared (variation in ES attributable to heterogeneity) = **95.9%**
Estimate of between-study variance Tau-squared = **0.0014**

Test of ES=0 : z= **43.08** p = **0.000**

Supplementary Figure 40 – Pooled sensitivity for diagnosing lung cancer on CT scans

Study	ES	[95% Conf. Interval]		% Weight
Ardila et al. 2019 (	**0.837**	**0.828**	**0.846**	**28.07**
Beig et al. 2019	**0.770**	**0.702**	**0.838**	**20.07**
Chakravarthy et al.	**0.950**	**0.911**	**0.989**	**24.94**
Hussein et al. 2019	**0.782**	**0.758**	**0.806**	**26.92**
D+L pooled ES	**0.837**	**0.780**	**0.894**	**100.00**

Heterogeneity chi-squared = **55.37** (d.f. = **3**) p = **0.000**
I-squared (variation in ES attributable to heterogeneity) = **94.6%**
Estimate of between-study variance Tau-squared = **0.0030**

Test of ES=0 : z= **28.79** p = **0.000**

Supplementary Figure 41 – Pooled specificity for diagnosing lung cancer on CT scans

Study	ES	[95% Conf.	Interval]	% Weight
Ardila et al. 2019 (	**0.950**	**0.945**	**0.955**	**27.01**
Beig et al. 2019	**0.630**	**0.551**	**0.709**	**22.51**
Chakravarthy et al.	**0.850**	**0.786**	**0.914**	**23.84**
Hussein et al. 2019	**0.846**	**0.825**	**0.867**	**26.65**
D+L pooled ES	**0.826**	**0.735**	**0.918**	**100.00**

Heterogeneity chi-squared = **158.55** (d.f. = **3**) p = **0.000**
I-squared (variation in ES attributable to heterogeneity) = **98.1%**
Estimate of between-study variance Tau-squared = **0.0080**

Test of ES=0 : z= **17.77** p = **0.000**

Supplementary Figure 42 – Pooled accuracy for diagnosing lung cancer on CT scans

Study	ES	[95% Conf. Interval]		% Weight
Alakwaa et al. 2017	0.866	0.833	0.899	17.37
Beig et al. 2019	0.680	0.604	0.756	12.07
Chakravarthy et al.	0.900	0.846	0.954	14.82
Hussein et al. 2019	0.817	0.795	0.840	18.34
Togacar et al. 2020	0.812	0.735	0.889	12.00
Togacar et al. 2020	0.891	0.830	0.952	13.92
Togacar et al. 2020	0.781	0.700	0.862	11.47
D+L pooled ES	0.827	0.784	0.870	100.00

Heterogeneity chi-squared = **32.75** (d.f. = **6**) p = **0.000**
I-squared (variation in ES attributable to heterogeneity) = **81.7%**
Estimate of between-study variance Tau-squared = **0.0025**

Test of ES=0 : z= **37.58** p = **0.000**

Supplementary Figure 43 – Pooled AUC for diagnosing abnormal Chest X-rays

Study	ES	[95% Conf.	Interval]	% Weight
Baltruschat et al. 2	**0.819**	**0.814**	**0.824**	**9.18**
Bar et al. 2018 (a)	**0.920**	**0.882**	**0.958**	**8.70**
Dunnmon et al. 2019	**0.960**	**0.943**	**0.977**	**9.09**
Hwang et al. 2019	**0.950**	**0.937**	**0.963**	**9.13**
Hwang et al. 2019 (a	**0.965**	**0.954**	**0.976**	**9.15**
Hwang et al. 2019 (b	**0.979**	**0.970**	**0.988**	**9.16**
Liu H et al. 2019 (o	**0.815**	**0.813**	**0.817**	**9.19**
Park et al. 2019 (e)	**0.985**	**0.968**	**1.002**	**9.09**
Wang et al. 2018 (a)	**0.821**	**0.793**	**0.849**	**8.93**
Wang et al. 2019 (o)	**0.896**	**0.892**	**0.900**	**9.19**
Yates et al. 2018	**0.980**	**0.976**	**0.984**	**9.19**
D+L pooled ES	**0.917**	**0.869**	**0.966**	**100.00**

Heterogeneity chi-squared = **7563.94** (d.f. = **10**) p = **0.000**
I-squared (variation in ES attributable to heterogeneity) = **99.9%**
Estimate of between-study variance Tau-squared = **0.0067**

Test of ES=0 : z= **37.00** p = **0.000**

Supplementary Figure 44 – Pooled sensitivity for diagnosing abnormal Chest X-rays

Study	ES	[95% Conf. Interval]		% Weight
Annarumma et al. 201	**0.650**	**0.643**	**0.657**	**14.32**
Dunnmon et al. 2019	**0.990**	**0.982**	**0.998**	**14.32**
Hwang et al. 2019	**0.887**	**0.869**	**0.905**	**14.27**
Hwang et al. 2019 (a	**0.920**	**0.904**	**0.936**	**14.29**
Hwang et al. 2019 (b	**0.979**	**0.970**	**0.988**	**14.32**
Wang et al. 2018 (a)	**0.740**	**0.708**	**0.772**	**14.17**
Yates et al. 2018	**0.946**	**0.940**	**0.952**	**14.32**
D+L pooled ES	**0.873**	**0.762**	**0.985**	**100.00**

Heterogeneity chi-squared = **5386.18** (d.f. = **6**) p = **0.000**
I-squared (variation in ES attributable to heterogeneity) = **99.9%**
Estimate of between-study variance Tau-squared = **0.0225**

Test of ES=0 : z= **15.39** p = **0.000**

Supplementary Figure 45 – Pooled specificity for diagnosing abnormal Chest X-rays

Study	ES	[95% Conf. Interval]		% Weight
Annarumma et al. 201	**0.940**	**0.936**	**0.944**	**14.84**
Dunnmon et al. 2019	**0.880**	**0.852**	**0.908**	**13.56**
Hwang et al. 2019	**0.669**	**0.642**	**0.696**	**13.58**
Hwang et al. 2019 (a	**0.950**	**0.937**	**0.963**	**14.56**
Hwang et al. 2019 (b	**0.880**	**0.860**	**0.900**	**14.15**
Park et al. 2019 (e)	**0.990**	**0.976**	**1.004**	**14.52**
Yates et al. 2018	**0.934**	**0.927**	**0.941**	**14.79**
D+L pooled ES	**0.894**	**0.860**	**0.929**	**100.00**

Heterogeneity chi-squared = **479.57** (d.f. = **6**) p = **0.000**
I-squared (variation in ES attributable to heterogeneity) = **98.7%**
Estimate of between-study variance Tau-squared = **0.0021**

Test of ES=0 : z= **51.14** p = **0.000**

Supplementary Figure 46– Pooled accuracy for diagnosing abnormal Chest X-rays

Study	ES	[95% Conf. Interval]		% Weight
Abiyev et al. 2018	**0.929**	**0.904**	**0.955**	**33.28**
Wang et al. 2018 (a)	**0.700**	**0.667**	**0.733**	**32.98**
Yates et al. 2018	**0.946**	**0.940**	**0.952**	**33.74**
D+L pooled ES	**0.859**	**0.736**	**0.983**	**100.00**

Heterogeneity chi-squared = **208.00** (d.f. = **2**) p = **0.000**
I-squared (variation in ES attributable to heterogeneity) = **99.0%**
Estimate of between-study variance Tau-squared = **0.0118**

Test of ES=0 : z= **13.64** p = **0.000**

Supplementary Figure 47 – Pooled PPV for diagnosing abnormal Chest X-rays

Study	ES	[95% Conf. Interval]		% Weight
Annarumma et al. 201	**0.610**	**0.602**	**0.618**	**33.34**
Wang et al. 2018 (a)	**0.943**	**0.926**	**0.960**	**33.31**
Yates et al. 2018	**0.998**	**0.997**	**0.999**	**33.35**
D+L pooled ES	**0.850**	**0.567**	**1.133**	**100.00**

Heterogeneity chi-squared = **9844.61** (d.f. = **2**) p = **0.000**
I-squared (variation in ES attributable to heterogeneity) = **100.0%**
Estimate of between-study variance Tau-squared = **0.0626**

Test of ES=0 : z= **5.89** p = **0.000**

Supplementary Figure 48 – Pooled F1 score for diagnosing abnormal Chest X-rays

Study	ES	[95% Conf. Interval]		% Weight
Annarumma et al. 201	**0.630**	**0.622**	**0.638**	**33.46**
Dunnmon et al. 2019	**0.930**	**0.908**	**0.952**	**33.35**
Wang et al. 2018 (a)	**0.720**	**0.688**	**0.752**	**33.19**
D+L pooled ES	**0.760**	**0.558**	**0.962**	**100.00**

Heterogeneity chi-squared = **669.61** (d.f. = **2**) p = **0.000**
I-squared (variation in ES attributable to heterogeneity) = **99.7%**
Estimate of between-study variance Tau-squared = **0.0318**

Test of ES=0 : z= **7.36** p = **0.000**

Supplementary Figure 49 – Pooled AUC for diagnosing atelectasis on Chest X-ray

Study	ES	[95% Conf. Interval]		% Weight
Baltruschat et al. 2	**0.803**	**0.798**	**0.808**	**25.86**
Liu H et al. 2019 (a	**0.781**	**0.779**	**0.783**	**25.94**
Rajpurkar et al. 201	**0.862**	**0.829**	**0.895**	**22.31**
Wang et al. 2019 (a)	**0.856**	**0.852**	**0.860**	**25.89**
D+L pooled ES	**0.824**	**0.783**	**0.866**	**100.00**

Heterogeneity chi-squared = **902.65** (d.f. = **3**) p = **0.000**
I-squared (variation in ES attributable to heterogeneity) = **99.7%**
Estimate of between-study variance Tau-squared = **0.0017**

Test of ES=0 : z= **38.87** p = **0.000**

Supplementary Figure 50 – Pooled AUC for diagnosing cardiomegaly on Chest X-ray

Study	ES	[95% Conf.	Interval]	% Weight
Baltruschat et al. 2	0.885	0.881	0.889	14.90
Bar et al. 2018 (b)	0.960	0.932	0.988	13.56
Cicero et al. 2017 (	0.875	0.862	0.888	14.60
Liu H et al. 2019 (b	0.885	0.883	0.887	14.93
Rajpurkar et al. 201	0.831	0.795	0.867	12.75
Singh et al. 2018 (d	0.936	0.918	0.954	14.33
Wang et al. 2019 (b)	0.957	0.955	0.959	14.93
D+L pooled ES	0.905	0.871	0.938	100.00

Heterogeneity chi-squared = **2257.90** (d.f. = **6**) p = **0.000**
I-squared (variation in ES attributable to heterogeneity) = **99.7%**
Estimate of between-study variance Tau-squared = **0.0019**

Test of ES=0 : z= **53.04** p = **0.000**

Supplementary Figure 51 – Pooled AUC for diagnosing consolidation on Chest X-ray

Study	ES	[95% Conf. Interval]		% Weight
Baltruschat et al. 2	**0.795**	**0.790**	**0.800**	**12.56**
Behzadi-Khormouji et	**0.985**	**0.975**	**0.995**	**12.54**
Behzadi-Khormouji et	**0.995**	**0.989**	**1.001**	**12.56**
Behzadi-Khormouji et	**0.869**	**0.841**	**0.896**	**12.36**
Cicero et al. 2017 (	**0.850**	**0.836**	**0.864**	**12.51**
Liu H et al. 2019 (i	**0.743**	**0.740**	**0.746**	**12.57**
Rajpurkar et al. 201	**0.893**	**0.863**	**0.923**	**12.33**
Wang et al. 2019 (i)	**0.870**	**0.866**	**0.874**	**12.57**
D+L pooled ES	**0.875**	**0.800**	**0.949**	**100.00**

Heterogeneity chi-squared = **8667.29** (d.f. = **7**) p = **0.000**
I-squared (variation in ES attributable to heterogeneity) = **99.9%**
Estimate of between-study variance Tau-squared = **0.0115**

Test of ES=0 : z= **23.02** p = **0.000**

Supplementary Figure 52 – Pooled sensitivity for diagnosing consolidation on Chest X-ray

Study	ES	[95% Conf.	Interval]	% Weight
Behzadi-Khormouji et	**0.971**	**0.958**	**0.985**	**25.02**
Behzadi-Khormouji et	**0.987**	**0.978**	**0.996**	**25.08**
Behzadi-Khormouji et	**0.958**	**0.942**	**0.974**	**24.96**
Cicero et al. 2017 (	**0.740**	**0.723**	**0.757**	**24.94**
D+L pooled ES	**0.914**	**0.816**	**1.013**	**100.00**

Heterogeneity chi-squared = **629.57** (d.f. = **3**) p = **0.000**
I-squared (variation in ES attributable to heterogeneity) = **99.5%**
Estimate of between-study variance Tau-squared = **0.0100**

Test of ES=0 : z= **18.23** p = **0.000**

Supplementary Figure 53 – Pooled specificity for diagnosing consolidation on Chest X-ray

Study	ES	[95% Conf.	Interval]	% Weight
Behzadi-Khormouji et	**0.859**	**0.830**	**0.887**	**25.04**
Behzadi-Khormouji et	**0.864**	**0.836**	**0.891**	**25.05**
Behzadi-Khormouji et	**0.530**	**0.490**	**0.571**	**24.64**
Cicero et al. 2017 (	**0.750**	**0.733**	**0.767**	**25.28**
D+L pooled ES	**0.751**	**0.637**	**0.866**	**100.00**

Heterogeneity chi-squared = **220.10** (d.f. = **3**) p = **0.000**
I-squared (variation in ES attributable to heterogeneity) = **98.6%**
Estimate of between-study variance Tau-squared = **0.0134**

Test of ES=0 : z= **12.89** p = **0.000**

Supplementary Figure 54 – Pooled accuracy for diagnosing consolidation on Chest X-ray

Study	ES	[95% Conf.	Interval]	% Weight
Behzadi-Khormouji et	**0.933**	**0.913**	**0.953**	**33.74**
Behzadi-Khormouji et	**0.945**	**0.926**	**0.964**	**33.91**
Behzadi-Khormouji et	**0.809**	**0.777**	**0.841**	**32.34**
D+L pooled ES	**0.897**	**0.828**	**0.966**	**100.00**

Heterogeneity chi-squared = **54.81** (d.f. = **2**) p = **0.000**
I-squared (variation in ES attributable to heterogeneity) = **96.4%**
Estimate of between-study variance Tau-squared = **0.0035**

Test of ES=0 : z= **25.59** p = **0.000**

Supplementary Figure 55 – Pooled AUC for diagnosing edema on Chest X-ray

Study	ES	[95% Conf. Interval]		% Weight
Baltruschat et al. 2	0.891	0.887	0.895	20.23
Cicero et al. 2017 (	0.868	0.855	0.881	19.98
Liu H et al. 2019 (j	0.842	0.840	0.844	20.25
Rajpurkar et al. 201	0.924	0.899	0.949	19.29
Wang et al. 2019 (j)	0.943	0.940	0.946	20.25
D+L pooled ES	0.893	0.843	0.944	100.00

Heterogeneity chi-squared = **3149.41** (d.f. = **4**) p = **0.000**
I-squared (variation in ES attributable to heterogeneity) = **99.9%**
Estimate of between-study variance Tau-squared = **0.0033**

Test of ES=0 : z= **34.47** p = **0.000**

Supplementary Figure 56 – Pooled AUC for diagnosing effusion on Chest X-ray

Study	ES	[95% Conf. Interval]		% Weight
Baltruschat et al. 2	**0.871**	**0.867**	**0.875**	**14.51**
Cicero et al. 2017 (	**0.962**	**0.954**	**0.970**	**14.46**
Liu H et al. 2019 (c	**0.832**	**0.830**	**0.834**	**14.52**
Park et al. 2019 (c)	**0.995**	**0.985**	**1.005**	**14.42**
Rajpurkar et al. 201	**0.901**	**0.872**	**0.930**	**13.69**
Singh et al. 2018 (b	**0.863**	**0.838**	**0.888**	**13.88**
Wang et al. 2019 (c)	**0.919**	**0.916**	**0.922**	**14.51**
D+L pooled ES	**0.906**	**0.862**	**0.950**	**100.00**

Heterogeneity chi-squared = **3179.51** (d.f. = **6**) p = **0.000**
I-squared (variation in ES attributable to heterogeneity) = **99.8%**
Estimate of between-study variance Tau-squared = **0.0035**

Test of ES=0 : z= **40.30** p = **0.000**

Supplementary Figure 57 – Pooled AUC for diagnosing emphysema on Chest X-ray

Study	ES	[95% Conf.	Interval]	% Weight
Baltruschat et al. 2	**0.892**	**0.888**	**0.896**	**27.40**
Liu H et al. 2019 (k	**0.921**	**0.919**	**0.923**	**27.51**
Rajpurkar et al. 201	**0.704**	**0.660**	**0.748**	**17.60**
Wang et al. 2019 (k)	**0.959**	**0.957**	**0.961**	**27.48**
D+L pooled ES	**0.885**	**0.855**	**0.916**	**100.00**

Heterogeneity chi-squared = **1103.77** (d.f. = **3**) p = **0.000**
I-squared (variation in ES attributable to heterogeneity) = **99.7%**
Estimate of between-study variance Tau-squared = **0.0009**

Test of ES=0 : z= **56.88** p = **0.000**

Supplementary Figure 58 – Pooled AUC for diagnosing fibrosis on Chest X-ray

Study	ES	[95% Conf.	Interval]	% Weight
Baltruschat et al. 2	**0.800**	**0.795**	**0.805**	**26.29**
Liu H et al. 2019 (l	**0.835**	**0.833**	**0.837**	**26.39**
Rajpurkar et al. 201	**0.806**	**0.768**	**0.844**	**20.97**
Wang et al. 2019 (l)	**0.889**	**0.885**	**0.893**	**26.35**
D+L pooled ES	**0.834**	**0.796**	**0.872**	**100.00**

Heterogeneity chi-squared = **863.35** (d.f. = **3**) p = **0.000**
I-squared (variation in ES attributable to heterogeneity) = **99.7%**
Estimate of between-study variance Tau-squared = **0.0014**

Test of ES=0 : z= **42.85** p = **0.000**

Supplementary Figure 59 – Pooled AUC for diagnosing hiatus hernia on Chest X-ray

Study	ES	[95% Conf.	Interval]	% Weight
Baltruschat et al. 2	**0.855**	**0.850**	**0.860**	**26.15**
Liu H et al. 2019 (n	**0.911**	**0.909**	**0.913**	**26.25**
Rajpurkar et al. 201	**0.851**	**0.817**	**0.885**	**21.37**
Wang et al. 2019 (n)	**0.951**	**0.948**	**0.954**	**26.23**
D+L pooled ES	**0.894**	**0.858**	**0.930**	**100.00**

Heterogeneity chi-squared = **1393.94** (d.f. = **3**) p = **0.000**
I-squared (variation in ES attributable to heterogeneity) = **99.8%**
Estimate of between-study variance Tau-squared = **0.0013**

Test of ES=0 : z= **48.04** p = **0.000**

Supplementary Figure 60 – Pooled AUC for diagnosing infiltration on Chest X-ray

Study	ES	[95% Conf. Interval]		% Weight
Baltruschat et al. 2	**0.699**	**0.693**	**0.705**	**26.33**
Liu H et al. 2019 (d	**0.700**	**0.697**	**0.703**	**26.44**
Rajpurkar et al. 201	**0.721**	**0.678**	**0.764**	**20.87**
Wang et al. 2019 (d)	**0.776**	**0.771**	**0.781**	**26.36**
D+L pooled ES	**0.724**	**0.682**	**0.767**	**100.00**

Heterogeneity chi-squared = **694.76** (d.f. = **3**) p = **0.000**
I-squared (variation in ES attributable to heterogeneity) = **99.6%**
Estimate of between-study variance Tau-squared = **0.0018**

Test of ES=0 : z= **33.30** p = **0.000**

Supplementary Figure 61 – Pooled AUC for diagnosing a mass on Chest X-ray

Study	ES	[95% Conf.	Interval]	% Weight
Baltruschat et al. 2	**0.822**	**0.817**	**0.827**	**11.39**
Cha et al. 2019 (a)	**0.732**	**0.709**	**0.755**	**10.95**
Cha et al. 2019 (b)	**0.899**	**0.873**	**0.925**	**10.78**
Liu H et al. 2019 (e	**0.815**	**0.813**	**0.817**	**11.41**
Majkowska et al. 201	**0.910**	**0.897**	**0.923**	**11.25**
Majkowska et al. 201	**0.940**	**0.929**	**0.951**	**11.31**
Rajpurkar et al. 201	**0.909**	**0.881**	**0.937**	**10.73**
Singh et al. 2018 (a	**0.843**	**0.817**	**0.869**	**10.78**
Wang et al. 2019 (e)	**0.905**	**0.901**	**0.909**	**11.40**
D+L pooled ES	**0.864**	**0.827**	**0.901**	**100.00**

Heterogeneity chi-squared = **2353.83** (d.f. = **8**) p = **0.000**
I-squared (variation in ES attributable to heterogeneity) = **99.7%**
Estimate of between-study variance Tau-squared = **0.0031**

Test of ES=0 : z= **45.80** p = **0.000**

Supplementary Figure 62 – Pooled sensitivity for diagnosing a mass on Chest X-ray

Study	ES	[95% Conf.	Interval]	% Weight
Cha et al. 2019 (a)	**0.768**	**0.747**	**0.789**	**19.97**
Cha et al. 2019 (b)	**0.920**	**0.896**	**0.944**	**19.94**
Majkowska et al. 201	**0.534**	**0.511**	**0.557**	**19.95**
Majkowska et al. 201	**0.861**	**0.846**	**0.876**	**20.04**
Pesce et al. 2019	**0.920**	**0.914**	**0.926**	**20.09**
D+L pooled ES	**0.801**	**0.683**	**0.919**	**100.00**

Heterogeneity chi-squared = **1162.43** (d.f. = **4**) p = **0.000**
I-squared (variation in ES attributable to heterogeneity) = **99.7%**
Estimate of between-study variance Tau-squared = **0.0180**

Test of ES=0 : z= **13.30** p = **0.000**

Supplementary Figure 63 – Pooled AUC for diagnosing lung nodules on Chest X-ray

Study	ES	[95% Conf.	Interval]	% Weight
Baltruschat et al. 2	**0.726**	**0.720**	**0.732**	**7.92**
Liang et al. 2019	**0.916**	**0.862**	**0.970**	**6.98**
Liu H et al. 2019 (f	**0.765**	**0.763**	**0.767**	**7.93**
Majkowska et al. 201	**0.720**	**0.699**	**0.741**	**7.78**
Majkowska et al. 201	**0.910**	**0.897**	**0.923**	**7.88**
Nam et al. 2018 (a)	**0.960**	**0.944**	**0.976**	**7.84**
Nam et al. 2018 (b)	**0.920**	**0.880**	**0.960**	**7.40**
Nam et al. 2018 (c)	**0.990**	**0.976**	**1.004**	**7.86**
Nam et al. 2018 (d)	**0.940**	**0.905**	**0.975**	**7.52**
Nam et al. 2018 (e)	**0.960**	**0.929**	**0.991**	**7.59**
Park et al. 2019 (a)	**0.971**	**0.948**	**0.994**	**7.74**
Rajpurkar et al. 201	**0.894**	**0.865**	**0.923**	**7.63**
Wang et al. 2019 (f)	**0.832**	**0.827**	**0.837**	**7.93**
D+L pooled ES	**0.884**	**0.842**	**0.925**	**100.00**

Heterogeneity chi-squared = **3267.83** (d.f. = **12**) p = **0.000**
I-squared (variation in ES attributable to heterogeneity) = **99.6%**
Estimate of between-study variance Tau-squared = **0.0057**

Test of ES=0 : z= **41.74** p = **0.000**

Supplementary Figure 64 – Pooled sensitivity for diagnosing lung nodules on Chest X-ray

Study	ES	[95% Conf.	Interval]	% Weight
Liang et al. 2019	**0.766**	**0.683**	**0.849**	**9.70**
Majkowska et al. 201	**0.441**	**0.418**	**0.464**	**10.17**
Majkowska et al. 201	**0.824**	**0.807**	**0.841**	**10.18**
Nam et al. 2018 (b)	**0.790**	**0.731**	**0.849**	**9.94**
Nam et al. 2018 (c)	**0.911**	**0.870**	**0.952**	**10.07**
Nam et al. 2018 (d)	**0.712**	**0.646**	**0.778**	**9.88**
Nam et al. 2018 (e)	**0.880**	**0.828**	**0.932**	**10.00**
Park et al. 2019 (a)	**0.843**	**0.793**	**0.893**	**10.01**
Sim et al. 2019	**0.673**	**0.640**	**0.706**	**10.12**
Wang et al. 2017	**0.659**	**0.598**	**0.720**	**9.92**
D+L pooled ES	**0.750**	**0.634**	**0.866**	**100.00**

Heterogeneity chi-squared = **879.60** (d.f. = **9**) p = **0.000**
I-squared (variation in ES attributable to heterogeneity) = **99.0%**
Estimate of between-study variance Tau-squared = **0.0343**

Test of ES=0 : z= **12.66** p = **0.000**

Supplementary Figure 65 – Pooled specificity for diagnosing lung nodules on Chest X-ray

Study	ES	[95% Conf.	Interval]	% Weight
Liang et al. 2019	**0.887**	**0.825**	**0.949**	**9.18**
Majkowska et al. 201	**0.975**	**0.968**	**0.982**	**13.82**
Majkowska et al. 201	**0.849**	**0.833**	**0.865**	**13.47**
Nam et al. 2018 (b)	**0.950**	**0.918**	**0.982**	**12.26**
Nam et al. 2018 (c)	**0.980**	**0.960**	**1.000**	**13.19**
Nam et al. 2018 (d)	**1.000**	**1.000**	**1.000**	**13.92**
Nam et al. 2018 (e)	**0.930**	**0.889**	**0.971**	**11.36**
Wang et al. 2017	**0.959**	**0.933**	**0.985**	**12.79**
D+L pooled ES	**0.944**	**0.912**	**0.976**	**100.00**

Heterogeneity chi-squared = **442.61** (d.f. = **7**) p = **0.000**
I-squared (variation in ES attributable to heterogeneity) = **98.4%**
Estimate of between-study variance Tau-squared = **0.0019**

Test of ES=0 : z= **57.39** p = **0.000**

Supplementary Figure 66 – Pooled PPV for diagnosing lung nodules on Chest X-ray

Study	ES	[95% Conf.	Interval]	% Weight
Liang et al. 2019	**0.857**	**0.788**	**0.926**	**13.82**
Majkowska et al. 201	**0.777**	**0.758**	**0.796**	**14.39**
Majkowska et al. 201	**0.492**	**0.470**	**0.514**	**14.37**
Nam et al. 2018 (b)	**0.949**	**0.917**	**0.981**	**14.30**
Nam et al. 2018 (c)	**0.991**	**0.977**	**1.005**	**14.41**
Nam et al. 2018 (d)	**1.000**	**1.000**	**1.000**	**14.44**
Nam et al. 2018 (e)	**0.951**	**0.916**	**0.986**	**14.27**
D+L pooled ES	**0.860**	**0.736**	**0.984**	**100.00**

Heterogeneity chi-squared = **2583.29** (d.f. = **6**) p = **0.000**
I-squared (variation in ES attributable to heterogeneity) = **99.8%**
Estimate of between-study variance Tau-squared = **0.0277**

Test of ES=0 : z= **13.59** p = **0.000**

Supplementary Figure 67 – Pooled F1 score for diagnosing lung nodules on Chest X-ray

Study	ES	[95% Conf. Interval]		% Weight
Nam et al. 2018 (b)	**0.870**	**0.821**	**0.919**	**24.23**
Nam et al. 2018 (c)	**0.949**	**0.917**	**0.981**	**27.74**
Nam et al. 2018 (d)	**0.832**	**0.778**	**0.886**	**23.03**
Nam et al. 2018 (e)	**0.912**	**0.867**	**0.957**	**24.99**
D+L pooled ES	**0.894**	**0.842**	**0.945**	**100.00**

Heterogeneity chi-squared = **16.14** (d.f. = **3**) p = **0.001**
I-squared (variation in ES attributable to heterogeneity) = **81.4%**
Estimate of between-study variance Tau-squared = **0.0022**

Test of ES=0 : z= **34.10** p = **0.000**

Supplementary Figure 68 – Pooled AUC for diagnosing pleural thickening on Chest X-ray

Study	ES	[95% Conf.	Interval]	% Weight
Baltruschat et al. 2	**0.790**	**0.785**	**0.795**	**25.72**
Liu H et al. 2019 (m	**0.791**	**0.789**	**0.793**	**25.77**
Rajpurkar et al. 201	**0.798**	**0.760**	**0.836**	**22.77**
Wang et al. 2019 (m)	**0.883**	**0.879**	**0.887**	**25.75**
D+L pooled ES	**0.816**	**0.762**	**0.870**	**100.00**

Heterogeneity chi-squared = **1613.85** (d.f. = **3**) p = **0.000**
I-squared (variation in ES attributable to heterogeneity) = **99.8%**
Estimate of between-study variance Tau-squared = **0.0029**

Test of ES=0 : z= **29.87** p = **0.000**

Supplementary Figure 69 – Pooled AUC for diagnosing pneumonia on Chest X-ray

Study	ES	[95% Conf.	Interval]	% Weight
Baltruschat et al. 2	**0.744**	**0.738**	**0.750**	**7.80**
Kermany et al. 2018	**0.968**	**0.954**	**0.982**	**7.78**
Liang et al. 2019 (a	**0.953**	**0.936**	**0.970**	**7.76**
Liang et al. 2019 (b	**0.840**	**0.811**	**0.869**	**7.68**
Liang et al. 2019 (c	**0.769**	**0.736**	**0.802**	**7.64**
Liang et al. 2019 (d	**0.655**	**0.618**	**0.692**	**7.60**
Liang et al. 2019 (e	**0.930**	**0.910**	**0.950**	**7.74**
Liu H et al. 2019 (g	**0.719**	**0.716**	**0.722**	**7.81**
Patel et al. 2019	**0.938**	**0.871**	**1.005**	**7.17**
Rajpurkar et al. 201	**0.851**	**0.817**	**0.885**	**7.63**
Wang et al. 2019 (g)	**0.869**	**0.865**	**0.873**	**7.80**
Zech et al. 2018 (a)	**0.931**	**0.928**	**0.934**	**7.81**
Zech et al. 2018 (b)	**0.815**	**0.803**	**0.827**	**7.78**
D+L pooled ES	**0.845**	**0.782**	**0.907**	**100.00**

Heterogeneity chi-squared = **13615.43** (d.f. = **12**) p = **0.000**
I-squared (variation in ES attributable to heterogeneity) = **99.9%**
Estimate of between-study variance Tau-squared = **0.0130**

Test of ES=0 : z= **26.49** p = **0.000**

Supplementary Figure 70 – Pooled sensitivity for diagnosing pneumonia on Chest X-ray

Study	ES	[95% Conf.	Interval]	% Weight
Kermany et al. 2018	**0.932**	**0.912**	**0.952**	**9.92**
Liang et al. 2019 (a	**0.967**	**0.953**	**0.981**	**10.96**
Liang et al. 2019 (b	**0.951**	**0.934**	**0.968**	**10.45**
Liang et al. 2019 (c	**0.964**	**0.949**	**0.979**	**10.86**
Liang et al. 2019 (d	**0.841**	**0.812**	**0.870**	**8.19**
Liang et al. 2019 (e	**0.967**	**0.953**	**0.981**	**10.96**
Patel et al. 2019	**0.900**	**0.817**	**0.983**	**2.37**
Togacar et al. 2019	**0.985**	**0.979**	**0.991**	**12.02**
Zech et al. 2018 (a)	**0.950**	**0.948**	**0.952**	**12.21**
Zech et al. 2018 (b)	**0.974**	**0.969**	**0.979**	**12.07**
D+L pooled ES	**0.951**	**0.936**	**0.965**	**100.00**

Heterogeneity chi-squared = **244.15** (d.f. = **9**) p = **0.000**
I-squared (variation in ES attributable to heterogeneity) = **96.3%**
Estimate of between-study variance Tau-squared = **0.0004**

Test of ES=0 : z= **130.79** p = **0.000**

Supplementary Figure 71 – Pooled specificity for diagnosing pneumonia on Chest X-ray

Study	ES	[95% Conf.	Interval]	% Weight
Kermany et al. 2018	**0.901**	**0.878**	**0.924**	**20.16**
Patel et al. 2019	**0.767**	**0.650**	**0.884**	**19.25**
Togacar et al. 2019	**0.979**	**0.972**	**0.986**	**20.20**
Zech et al. 2018 (a)	**0.706**	**0.701**	**0.711**	**20.20**
Zech et al. 2018 (b)	**0.230**	**0.217**	**0.243**	**20.19**
D+L pooled ES	**0.716**	**0.480**	**0.953**	**100.00**

Heterogeneity chi-squared = **10719.13** (d.f. = **4**) p = **0.000**
I-squared (variation in ES attributable to heterogeneity) = **100.0%**
Estimate of between-study variance Tau-squared = **0.0721**

Test of ES=0 : z= **5.94** p = **0.000**

Supplementary Figure 72 – Pooled accuracy for diagnosing pneumonia on Chest X-ray

Study	ES	[95% Conf. Interval]		% Weight
Kermany et al. 2018	**0.928**	**0.908**	**0.948**	**19.98**
Stephen et al. 2019	**0.937**	**0.927**	**0.948**	**20.00**
Togacar et al. 2019	**0.982**	**0.976**	**0.988**	**20.01**
Zech et al. 2018 (a)	**0.732**	**0.727**	**0.737**	**20.01**
Zech et al. 2018 (b)	**0.238**	**0.224**	**0.252**	**20.00**
D+L pooled ES	**0.763**	**0.559**	**0.968**	**100.00**

Heterogeneity chi-squared = **11481.58** (d.f. = **4**) p = **0.000**
I-squared (variation in ES attributable to heterogeneity) = **100.0%**
Estimate of between-study variance Tau-squared = **0.0546**

Test of ES=0 : z= **7.30** p = **0.000**

Supplementary Figure 73 – Pooled PPV for diagnosing pneumonia on Chest X-ray

Study	ES	[95% Conf.	Interval]	% Weight
Liang et al. 2019 (a	**0.891**	**0.867**	**0.915**	**12.50**
Liang et al. 2019 (b	**0.723**	**0.688**	**0.758**	**12.49**
Liang et al. 2019 (c	**0.792**	**0.760**	**0.824**	**12.49**
Liang et al. 2019 (d	**0.916**	**0.894**	**0.938**	**12.50**
Liang et al. 2019 (e	**0.857**	**0.830**	**0.884**	**12.50**
Togacar et al. 2019	**0.979**	**0.972**	**0.986**	**12.51**
Zech et al. 2018 (a)	**0.279**	**0.274**	**0.284**	**12.51**
Zech et al. 2018 (b)	**0.013**	**0.009**	**0.017**	**12.51**
D+L pooled ES	**0.681**	**0.367**	**0.995**	**100.00**

Heterogeneity chi-squared = **71571.34** (d.f. = **7**) p = **0.000**
I-squared (variation in ES attributable to heterogeneity) = **100.0%**
Estimate of between-study variance Tau-squared = **0.2056**

Test of ES=0 : z= **4.25** p = **0.000**

Supplementary Figure 74 – Pooled F1 score for diagnosing pneumonia on Chest X-ray

Study	ES	[95% Conf.	Interval]	% Weight
Liang et al. 2019 (a	**0.927**	**0.907**	**0.947**	**15.25**
Liang et al. 2019 (b	**0.822**	**0.792**	**0.852**	**14.84**
Liang et al. 2019 (c	**0.869**	**0.843**	**0.895**	**15.01**
Liang et al. 2019 (d	**0.877**	**0.851**	**0.903**	**15.04**
Liang et al. 2019 (e	**0.908**	**0.885**	**0.931**	**15.17**
Patel et al. 2019	**0.800**	**0.689**	**0.911**	**9.09**
Togacar et al. 2019	**0.982**	**0.976**	**0.988**	**15.59**
D+L pooled ES	**0.889**	**0.838**	**0.941**	**100.00**

Heterogeneity chi-squared = **252.61** (d.f. = **6**) p = **0.000**
I-squared (variation in ES attributable to heterogeneity) = **97.6%**
Estimate of between-study variance Tau-squared = **0.0045**

Test of ES=0 : z= **33.71** p = **0.000**

Supplementary Figure 75 – Pooled AUC for diagnosing pneumothorax on Chest X-ray

Study	ES	[95% Conf.	Interval]	% Weight
Baltruschat et al. 2	**0.870**	**0.866**	**0.874**	**7.16**
Cicero et al. 2017 (	**0.861**	**0.847**	**0.875**	**7.12**
Liu H et al. 2019 (h	**0.866**	**0.864**	**0.868**	**7.17**
Majkowska et al. 201	**0.950**	**0.940**	**0.960**	**7.14**
Majkowska et al. 201	**0.940**	**0.929**	**0.951**	**7.14**
Park et al. 2019	**0.984**	**0.973**	**0.995**	**7.14**
Park et al. 2019 (d)	**0.995**	**0.985**	**1.005**	**7.14**
Rajpurkar et al. 201	**0.944**	**0.922**	**0.966**	**7.05**
Taylor et al. 2018 (	**0.960**	**0.951**	**0.969**	**7.15**
Taylor et al. 2018 (	**0.940**	**0.930**	**0.950**	**7.14**
Taylor et al. 2018 (	**0.750**	**0.747**	**0.753**	**7.16**
Taylor et al. 2018 (	**0.750**	**0.747**	**0.753**	**7.16**
Wang et al. 2019	**0.991**	**0.982**	**1.000**	**7.15**
Wang et al. 2019 (h)	**0.941**	**0.938**	**0.944**	**7.16**
D+L pooled ES	**0.910**	**0.863**	**0.957**	**100.00**

Heterogeneity chi-squared = **19917.43** (d.f. = **13**) p = **0.000**
I-squared (variation in ES attributable to heterogeneity) = **99.9%**
Estimate of between-study variance Tau-squared = **0.0079**

Test of ES=0 : z= **38.24** p = **0.000**

Supplementary Figure 76 – Pooled sensitivity for diagnosing pneumothorax on Chest X-ray

Study	ES	[95% Conf.	Interval]	% Weight
Cicero et al. 2017 (	**0.780**	**0.764**	**0.796**	**11.11**
Majkowska et al. 201	**0.648**	**0.626**	**0.670**	**11.11**
Majkowska et al. 201	**0.728**	**0.708**	**0.748**	**11.11**
Park et al. 2019	**0.897**	**0.870**	**0.924**	**11.10**
Park et al. 2019 (d)	**1.000**	**1.000**	**1.000**	**11.12**
Taylor et al. 2018 (	**0.800**	**0.782**	**0.818**	**11.11**
Taylor et al. 2018 (	**0.840**	**0.824**	**0.856**	**11.11**
Taylor et al. 2018 (	**0.280**	**0.277**	**0.283**	**11.12**
Taylor et al. 2018 (	**0.490**	**0.487**	**0.493**	**11.12**
D+L pooled ES	**0.718**	**0.433**	**1.004**	**100.00**

Heterogeneity chi-squared = **4.1e+05** (d.f. = **8**) p = **0.000**
I-squared (variation in ES attributable to heterogeneity) = **100.0%**
Estimate of between-study variance Tau-squared = **0.1909**

Test of ES=0 : z= **4.93** p = **0.000**

Supplementary Figure 77 – Pooled specificity for diagnosing pneumothorax on Chest X-ray

Study	ES	[95% Conf.	Interval]	% Weight
Cicero et al. 2017 (	**0.780**	**0.764**	**0.796**	**12.40**
Majkowska et al. 201	**0.997**	**0.994**	**1.000**	**12.58**
Majkowska et al. 201	**0.908**	**0.895**	**0.921**	**12.47**
Park et al. 2019	**0.964**	**0.948**	**0.980**	**12.40**
Taylor et al. 2018 (	**0.970**	**0.963**	**0.977**	**12.54**
Taylor et al. 2018 (	**0.900**	**0.887**	**0.913**	**12.46**
Taylor et al. 2018 (	**0.970**	**0.969**	**0.971**	**12.58**
Taylor et al. 2018 (	**0.850**	**0.848**	**0.852**	**12.58**
D+L pooled ES	**0.918**	**0.870**	**0.965**	**100.00**

Heterogeneity chi-squared = **12176.25** (d.f. = **7**) p = **0.000**
I-squared (variation in ES attributable to heterogeneity) = **99.9%**
Estimate of between-study variance Tau-squared = **0.0047**

Test of ES=0 : z= **37.56** p = **0.000**

Supplementary Figure 78 – Pooled PPV for diagnosing pneumothorax on Chest X-ray

Study	ES	[95% Conf.	Interval]	% Weight
Majkowska et al. 201	**0.900**	**0.886**	**0.914**	**16.68**
Majkowska et al. 201	**0.487**	**0.465**	**0.509**	**16.63**
Taylor et al. 2018 (	**0.710**	**0.690**	**0.730**	**16.64**
Taylor et al. 2018 (	**0.450**	**0.428**	**0.472**	**16.63**
Taylor et al. 2018 (	**0.280**	**0.277**	**0.283**	**16.71**
Taylor et al. 2018 (	**0.150**	**0.148**	**0.152**	**16.71**
D+L pooled ES	**0.496**	**0.369**	**0.623**	**100.00**

Heterogeneity chi-squared = **18934.81** (d.f. = **5**) p = **0.000**
I-squared (variation in ES attributable to heterogeneity) = **100.0%**
Estimate of between-study variance Tau-squared = **0.0252**

Test of ES=0 : z= **7.64** p = **0.000**

Supplementary Figure 79 – Pooled AUC for diagnosing tuberculosis on Chest X-ray

Hwang et al. 2018 (f	**0.996**	**0.985**	**1.007**	**2.45**
Hwang et al. 2018 (g	**0.977**	**0.965**	**0.989**	**2.14**
Lakhani et al. 2017	**0.980**	**0.958**	**1.002**	**0.60**
Lakhani et al. 2017	**0.970**	**0.943**	**0.997**	**0.41**
Lakhani et al. 2017	**0.990**	**0.974**	**1.006**	**1.16**
Pasa et al. 2019	**0.925**	**0.890**	**0.960**	**0.25**
Qin et al. 2019 (a)	**0.940**	**0.927**	**0.953**	**1.61**
Qin et al. 2019 (b)	**0.940**	**0.927**	**0.953**	**1.61**
Qin et al. 2019 (c)	**0.920**	**0.905**	**0.935**	**1.25**
D+L pooled ES	**0.979**	**0.978**	**0.981**	**100.00**

Heterogeneity chi-squared = **4153.52** (d.f. = **15**) p = **0.000**
I-squared (variation in ES attributable to heterogeneity) = **99.6%**
Estimate of between-study variance Tau-squared = **0.0000**

Test of ES=0 : z= **1097.17** p = **0.000**

Supplementary Figure 80 – Pooled sensitivity for diagnosing tuberculosis on Chest X-ray

Study	ES	[95% Conf.	Interval]	% Weight
Hwang et al. 2018 (b	**0.952**	**0.921**	**0.983**	**0.12**
Hwang et al. 2018 (c	**0.943**	**0.905**	**0.981**	**0.08**
Hwang et al. 2018 (d	**1.000**	**1.000**	**1.000**	**32.85**
Hwang et al. 2018 (e	**1.000**	**1.000**	**1.000**	**32.85**
Hwang et al. 2018 (f	**1.000**	**1.000**	**1.000**	**32.84**
Hwang et al. 2018 (g	**0.947**	**0.930**	**0.964**	**0.37**
Lakhani et al. 2017	**0.920**	**0.877**	**0.963**	**0.06**
Lakhani et al. 2017	**0.920**	**0.877**	**0.963**	**0.06**
Lakhani et al. 2017	**0.973**	**0.947**	**0.999**	**0.17**
Lakhani et al. 2017	**0.973**	**0.947**	**0.999**	**0.17**
Qin et al. 2019 (a)	**0.580**	**0.552**	**0.608**	**0.14**
Qin et al. 2019 (b)	**0.710**	**0.684**	**0.736**	**0.17**
Qin et al. 2019 (c)	**0.470**	**0.442**	**0.498**	**0.14**
D+L pooled ES	**0.998**	**0.997**	**0.999**	**100.00**

Heterogeneity chi-squared = **2791.50** (d.f. = **12**) p = **0.000**
I-squared (variation in ES attributable to heterogeneity) = **99.6%**
Estimate of between-study variance Tau-squared = **0.0000**

Test of ES=0 : z= **1850.98** p = **0.000**

Supplementary Figure 81 – Pooled specificity for diagnosing tuberculosis on Chest X-ray

Study	ES	[95% Conf.	Interval]	% Weight
Hwang et al. 2018 (b	**1.000**	**1.000**	**1.000**	**49.49**
Hwang et al. 2018 (c	**0.957**	**0.923**	**0.991**	**0.02**
Hwang et al. 2018 (d	**0.914**	**0.872**	**0.956**	**0.02**
Hwang et al. 2018 (e	**0.980**	**0.959**	**1.001**	**0.06**
Hwang et al. 2018 (f	**0.938**	**0.897**	**0.979**	**0.02**
Hwang et al. 2018 (g	**0.911**	**0.889**	**0.933**	**0.06**
Lakhani et al. 2017	**0.947**	**0.911**	**0.983**	**0.02**
Lakhani et al. 2017	**0.987**	**0.969**	**1.005**	**0.08**
Lakhani et al. 2017	**0.947**	**0.911**	**0.983**	**0.02**
Lakhani et al. 2017	**1.000**	**1.000**	**1.000**	**49.45**
Qin et al. 2019 (a)	**0.970**	**0.960**	**0.980**	**0.30**
Qin et al. 2019 (b)	**0.960**	**0.949**	**0.971**	**0.23**
Qin et al. 2019 (c)	**0.960**	**0.949**	**0.971**	**0.23**
D+L pooled ES	**1.000**	**0.999**	**1.000**	**100.00**

Heterogeneity chi-squared = **253.28** (d.f. = **12**) p = **0.000**
I-squared (variation in ES attributable to heterogeneity) = **95.3%**
Estimate of between-study variance Tau-squared = **0.0000**

Test of ES=0 : z= **3707.41** p = **0.000**

Supplementary Figure 82– Pooled accuracy for diagnosing tuberculosis on Chest X-ray

Study	ES	[95% Conf.	Interval]	% Weight
Lakhani et al. 2017	**0.933**	**0.893**	**0.973**	**9.59**
Lakhani et al. 2017	**0.953**	**0.919**	**0.987**	**10.87**
Lakhani et al. 2017	**0.960**	**0.929**	**0.991**	**11.43**
Lakhani et al. 2017	**0.987**	**0.969**	**1.005**	**14.33**
Pasa et al. 2019	**0.862**	**0.816**	**0.908**	**8.54**
Qin et al. 2019 (a)	**0.940**	**0.927**	**0.953**	**15.19**
Qin et al. 2019 (b)	**0.940**	**0.927**	**0.953**	**15.19**
Qin et al. 2019 (c)	**0.920**	**0.905**	**0.935**	**14.86**
D+L pooled ES	**0.940**	**0.921**	**0.959**	**100.00**

Heterogeneity chi-squared = **45.40** (d.f. = **7**) p = **0.000**
I-squared (variation in ES attributable to heterogeneity) = **84.6%**
Estimate of between-study variance Tau-squared = **0.0006**

Test of ES=0 : z= **95.85** p = **0.000**

SupplementaryFigure 83 – Pooled AUC for diagnosing breast cancer on mammogram

Study	ES	[95% Conf.	Interval]	% Weight
Agnes et al. 2020	**0.990**	**0.979**	**1.001**	**2.65**
Akselrod-Ballin et a	**0.720**	**0.653**	**0.787**	**2.08**
Al-Masni et al. 2018	**0.965**	**0.931**	**0.998**	**2.50**
Antropova et al. 201	**0.860**	**0.817**	**0.903**	**2.39**
Arevalo et al. 2016	**0.826**	**0.799**	**0.853**	**2.55**
Becker et al. 2017	**0.790**	**0.695**	**0.885**	**1.70**
Cai et al. 2019	**0.934**	**0.885**	**0.983**	**2.32**
Cogan et al. 2019	**0.951**	**0.913**	**0.989**	**2.45**
Dhungel et al. 2017	**0.910**	**0.848**	**0.972**	**2.15**
Duggento et al. 2019	**0.785**	**0.744**	**0.826**	**2.41**
Gao et al. 2018 (b)	**0.920**	**0.864**	**0.976**	**2.23**
Ha et al. 2019	**0.860**	**0.772**	**0.948**	**1.80**
Huyng et al. 2016	**0.810**	**0.779**	**0.841**	**2.52**
Kim et al. 2018	**0.841**	**0.802**	**0.880**	**2.44**
Kim et al. 2018	**0.906**	**0.890**	**0.922**	**2.62**
Kooi et al. 2017	**0.895**	**0.880**	**0.910**	**2.63**
Kooi et al. 2017	**0.906**	**0.902**	**0.910**	**2.66**
Kooi T et al. 2017	**0.804**	**0.786**	**0.822**	**2.61**
Li et al. 2019 (b)	**0.905**	**0.892**	**0.918**	**2.64**
McKinney et al. 2020	**0.889**	**0.885**	**0.893**	**2.67**
McKinney et al. 2020	**0.811**	**0.797**	**0.824**	**2.64**
Mendel et al. 2018 (	**0.880**	**0.808**	**0.952**	**2.01**
Qiu et al. 2017	**0.790**	**0.723**	**0.857**	**2.08**
Ragab et al. 2019 (a	**0.880**	**0.856**	**0.904**	**2.57**
Ragab et al. 2019 (b	**0.940**	**0.928**	**0.952**	**2.65**
Ribli et al. 2018	**0.950**	**0.910**	**0.990**	**2.43**
Rodriguez-Ruiz et al	**0.890**	**0.850**	**0.930**	**2.43**
Rodriguez-Ruiz et al	**0.840**	**0.826**	**0.854**	**2.64**
Samala et al. 2017	**0.820**	**0.795**	**0.845**	**2.57**
Shen et al. 2019 (a)	**0.860**	**0.825**	**0.895**	**2.48**
Shen et al. 2019 (b)	**0.850**	**0.814**	**0.886**	**2.47**
Shen et al. 2019 (c)	**0.950**	**0.909**	**0.991**	**2.41**
Sun et al. 2017	**0.880**	**0.857**	**0.903**	**2.58**
Teare et al. 2017	**0.922**	**0.894**	**0.950**	**2.54**
Wang et al. 2016	**0.890**	**0.819**	**0.961**	**2.03**
Wang et al. 2016	**0.900**	**0.859**	**0.941**	**2.41**
Wang et al. 2017	**0.971**	**0.952**	**0.990**	**2.61**
Wu et al. 2019 (a)	**0.895**	**0.865**	**0.925**	**2.53**
Wu et al. 2019 (b)	**0.876**	**0.859**	**0.893**	**2.62**
Yala et al. 2019	**0.680**	**0.670**	**0.690**	**2.65**
Yala et al. 2019	**0.820**	**0.815**	**0.825**	**2.66**
D+L pooled ES	**0.873**	**0.853**	**0.894**	**100.00**

Heterogeneity chi-squared = **3368.73** (d.f. = **40**) p = **0.000**
I-squared (variation in ES attributable to heterogeneity) = **98.8%**
Estimate of between-study variance Tau-squared = **0.0042**

Test of ES=0 : z= **82.81** p = **0.000**

Supplementary Figure 84 – Pooled sensitivity for diagnosing breast cancer on mammogram

Study	ES	[95% Conf.	Interval]	% Weight
Agnes et al. 2020	**0.960**	**0.939**	**0.981**	**4.23**
Al-Masni et al. 2018	**1.000**	**1.000**	**1.000**	**4.24**
Bandeira Diniz et al	**0.904**	**0.863**	**0.945**	**4.18**
Bandeira Diniz et al	**0.915**	**0.883**	**0.947**	**4.21**
Becker et al. 2017	**0.716**	**0.610**	**0.822**	**3.89**
Cai et al. 2019	**0.870**	**0.804**	**0.936**	**4.10**
Duggento et al. 2019	**0.844**	**0.807**	**0.881**	**4.20**
Gao et al. 2018 (b)	**0.830**	**0.752**	**0.908**	**4.04**
Ha et al. 2019	**0.846**	**0.755**	**0.937**	**3.97**
Jung et al. 2018 (a)	**0.910**	**0.882**	**0.938**	**4.22**
Jung et al. 2018 (b)	**0.980**	**0.962**	**0.998**	**4.23**
Kim et al. 2018	**0.761**	**0.737**	**0.785**	**4.22**
Li et al. 2019	**0.956**	**0.947**	**0.965**	**4.24**
Li et al. 2019 (b)	**0.657**	**0.635**	**0.679**	**4.23**
McKinney et al. 2020	**0.681**	**0.676**	**0.687**	**4.24**
McKinney et al. 2020	**0.575**	**0.558**	**0.592**	**4.23**
Peng et al. 2016 (a)	**0.986**	**0.963**	**1.009**	**4.22**
Peng et al. 2016 (b)	**0.958**	**0.919**	**0.997**	**4.19**
Qiu et al. 2017	**0.631**	**0.551**	**0.711**	**4.03**
Ribli et al. 2018	**0.900**	**0.845**	**0.955**	**4.14**
Rodriguez-Ruiz et al	**0.860**	**0.816**	**0.904**	**4.18**
Teare et al. 2017	**0.901**	**0.870**	**0.932**	**4.21**
Wang et al. 2016	**0.890**	**0.847**	**0.933**	**4.18**
Wang et al. 2018	**0.874**	**0.836**	**0.912**	**4.19**
D+L pooled ES	**0.851**	**0.779**	**0.923**	**100.00**

Heterogeneity chi-squared = **16331.45** (d.f. = **23**) p = **0.000**
I-squared (variation in ES attributable to heterogeneity) = **99.9%**
Estimate of between-study variance Tau-squared = **0.0322**

Test of ES=0 : z= **23.04** p = **0.000**

Supplementary Figure 85 – Pooled specificity for diagnosing breast cancer on mammogram

Study	ES	[95% Conf.	Interval]	% Weight
Agnes et al. 2020	**0.960**	**0.939**	**0.981**	**5.85**
Al-Masni et al. 2018	**0.940**	**0.898**	**0.982**	**5.10**
Bandeira Diniz et al	**0.964**	**0.938**	**0.990**	**5.72**
Bandeira Diniz et al	**0.907**	**0.873**	**0.941**	**5.45**
Becker et al. 2017	**0.696**	**0.588**	**0.804**	**2.64**
Cai et al. 2019	**0.867**	**0.800**	**0.934**	**4.07**
Duggento et al. 2019	**0.624**	**0.576**	**0.673**	**4.84**
Gao et al. 2018 (b)	**0.940**	**0.891**	**0.989**	**4.82**
Ha et al. 2019	**0.882**	**0.800**	**0.964**	**3.49**
Kim et al. 2018	**0.885**	**0.867**	**0.903**	**5.94**
Li et al. 2019	**0.954**	**0.944**	**0.963**	**6.10**
Li et al. 2019 (b)	**0.953**	**0.943**	**0.963**	**6.09**
McKinney et al. 2020	**0.962**	**0.960**	**0.965**	**6.15**
McKinney et al. 2020	**0.865**	**0.853**	**0.877**	**6.05**
Peng et al. 2016 (a)	**0.893**	**0.832**	**0.954**	**4.34**
Peng et al. 2016 (b)	**0.889**	**0.827**	**0.951**	**4.29**
Qiu et al. 2017	**0.800**	**0.734**	**0.866**	**4.10**
Rodriguez-Ruiz et al	**0.790**	**0.738**	**0.842**	**4.72**
Teare et al. 2017	**0.783**	**0.740**	**0.826**	**5.08**
Wang et al. 2016	**0.900**	**0.859**	**0.941**	**5.16**
D+L pooled ES	**0.882**	**0.859**	**0.905**	**100.00**

Heterogeneity chi-squared = **667.25** (d.f. = **19**) p = **0.000**
I-squared (variation in ES attributable to heterogeneity) = **97.2%**
Estimate of between-study variance Tau-squared = **0.0023**

Test of ES=0 : z= **74.50** p = **0.000**

Supplementary Figure 86 – Pooled accuracy for diagnosing breast cancer on mammogram

Study	ES	[95% Conf.	Interval]	% Weight
Abdelsamea et al. 20	**0.952**	**0.913**	**0.991**	**3.60**
Agnes et al. 2020	**0.965**	**0.945**	**0.985**	**3.84**
Akselrod-Ballin et a	**0.770**	**0.707**	**0.833**	**3.15**
Al-Antari et al. 201	**0.929**	**0.887**	**0.970**	**3.56**
Al-Antari et al. 201	**0.990**	**0.980**	**0.999**	**3.91**
Al-Masni et al. 2018	**0.970**	**0.939**	**1.001**	**3.72**
Bandeira Diniz et al	**0.948**	**0.917**	**0.979**	**3.71**
Bandeira Diniz et al	**0.910**	**0.877**	**0.943**	**3.68**
Cai et al. 2019	**0.877**	**0.812**	**0.942**	**3.12**
Chougrad et al. 2018	**0.974**	**0.969**	**0.978**	**3.93**
Chougrad et al. 2018	**0.967**	**0.952**	**0.981**	**3.88**
Chougrad et al. 2018	**0.955**	**0.926**	**0.984**	**3.74**
Dhungel et al. 2017	**0.870**	**0.797**	**0.943**	**2.95**
Gao et al. 2018 (b)	**0.900**	**0.838**	**0.962**	**3.16**
Ha et al. 2019	**0.867**	**0.781**	**0.953**	**2.69**
Jadoon et al. 2016	**0.812**	**0.798**	**0.826**	**3.89**
Jiao et al. 2016	**0.967**	**0.947**	**0.987**	**3.84**
Jiao et al. 2018 (a)	**0.925**	**0.883**	**0.967**	**3.54**
Jiao et al. 2018 (b)	**0.974**	**0.949**	**0.999**	**3.78**
Li et al. 2019	**0.946**	**0.936**	**0.955**	**3.91**
Li et al. 2019 (b)	**0.909**	**0.896**	**0.922**	**3.89**
Peng et al. 2016 (a)	**0.960**	**0.922**	**0.998**	**3.60**
Peng et al. 2016 (b)	**0.920**	**0.867**	**0.973**	**3.34**
Ragab et al. 2019 (a	**0.710**	**0.676**	**0.744**	**3.67**
Ragab et al. 2019 (b	**0.736**	**0.714**	**0.758**	**3.82**
Sun et al. 2017	**0.824**	**0.797**	**0.851**	**3.76**
Wang et al. 2016	**0.850**	**0.769**	**0.931**	**2.78**
Wang et al. 2016	**0.897**	**0.855**	**0.939**	**3.55**
D+L pooled ES	**0.905**	**0.880**	**0.930**	**100.00**

Heterogeneity chi-squared = **1314.10** (d.f. = **27**) p = **0.000**
I-squared (variation in ES attributable to heterogeneity) = **97.9%**
Estimate of between-study variance Tau-squared = **0.0042**

Test of ES=0 : z= **70.75** p = **0.000**

Supplementary Figure 87 – Pooled AUC for diagnosing breast cancer on ultrasound

Study	ES	[95% Conf.	Interval]	% Weight
Antropova et al. 201	**0.900**	**0.882**	**0.918**	**8.27**
Becker et al. 2018	**0.840**	**0.788**	**0.892**	**6.58**
Byra et al. 2019 (a)	**0.890**	**0.840**	**0.940**	**6.68**
Byra et al. 2019 (b)	**0.893**	**0.846**	**0.940**	**6.84**
Byra et al. 2019 (c)	**0.881**	**0.818**	**0.944**	**5.90**
Cheng et al. 2016 (a	**0.896**	**0.870**	**0.922**	**7.95**
Ciritsis et al. 2019	**0.838**	**0.766**	**0.910**	**5.42**
Ciritsis et al. 2019	**0.967**	**0.914**	**1.020**	**6.49**
Fujioka et al. 2019	**0.913**	**0.863**	**0.963**	**6.66**
Hizukuri et al. 2018	**0.976**	**0.954**	**0.998**	**8.14**
Kim et al. 2012	**0.870**	**0.791**	**0.949**	**5.05**
Qi et al. 2019	**0.980**	**0.973**	**0.987**	**8.51**
Stoffel et al. 2018	**0.730**	**0.579**	**0.881**	**2.40**
Tanaka et al. 2019	**0.951**	**0.917**	**0.985**	**7.57**
Xiao et al. 2019	**0.930**	**0.895**	**0.965**	**7.54**
D+L pooled ES	**0.909**	**0.881**	**0.936**	**100.00**

Heterogeneity chi-squared = **169.24** (d.f. = **14**) p = **0.000**
I-squared (variation in ES attributable to heterogeneity) = **91.7%**
Estimate of between-study variance Tau-squared = **0.0023**

Test of ES=0 : z= **64.41** p = **0.000**

Supplementary Figure 88 – Pooled sensitivity for diagnosing breast cancer on ultrasound

Study	ES	[95% Conf. Interval]		% Weight
Becker et al. 2018	0.842	0.790	0.894	6.04
Byra et al. 2019 (a)	0.848	0.791	0.905	5.89
Byra et al. 2019 (b)	0.851	0.796	0.906	5.96
Byra et al. 2019 (c)	0.807	0.730	0.884	5.31
Cao et al. 2019	0.800	0.752	0.848	6.14
Cheng et al. 2016 (a	0.787	0.752	0.822	6.42
Choi et al. 2019	0.850	0.806	0.894	6.23
Fujioka et al. 2019	0.958	0.922	0.994	6.40
Hizukuri et al. 2018	0.930	0.894	0.966	6.40
Lin et al. 2014	0.912	0.843	0.981	5.56
Qi et al. 2019	0.874	0.856	0.892	6.69
Stoffel et al. 2018	0.500	0.329	0.671	2.89
Tanaka et al. 2019	0.909	0.864	0.954	6.19
Xiao et al. 2019	0.887	0.844	0.930	6.25
Yap et al. 2018 (a)	0.980	0.964	0.996	6.71
Yap et al. 2018 (b)	0.920	0.878	0.962	6.28
Yap et al. 2019	0.568	0.468	0.668	4.63
D+L pooled ES	0.853	0.815	0.891	100.00

Heterogeneity chi-squared = **263.56** (d.f. = **16**) p = **0.000**
I-squared (variation in ES attributable to heterogeneity) = **93.9%**
Estimate of between-study variance Tau-squared = **0.0056**

Test of ES=0 : z= **43.73** p = **0.000**

Supplementary Figure 89 – Pooled specificity for diagnosing breast cancer on ultrasound

Study	ES	[95% Conf.	Interval]	% Weight
Becker et al. 2018	0.804	0.748	0.860	7.03
Byra et al. 2019 (a)	0.863	0.808	0.918	7.10
Byra et al. 2019 (b)	0.834	0.777	0.891	6.97
Byra et al. 2019 (c)	0.854	0.785	0.923	6.25
Cheng et al. 2016 (a	0.857	0.827	0.887	8.50
Choi et al. 2019	0.954	0.928	0.980	8.69
Fujioka et al. 2019	0.875	0.816	0.934	6.85
Hizukuri et al. 2018	0.931	0.895	0.967	8.22
Lin et al. 2014	0.936	0.876	0.995	6.81
Qi et al. 2019	0.967	0.957	0.976	9.19
Stoffel et al. 2018	1.000	1.000	1.000	9.27
Tanaka et al. 2019	0.870	0.817	0.923	7.21
Xiao et al. 2019	0.899	0.858	0.940	7.92
D+L pooled ES	0.901	0.870	0.931	100.00

Heterogeneity chi-squared = **348.20** (d.f. = **12**) p = **0.000**
I-squared (variation in ES attributable to heterogeneity) = **96.6%**
Estimate of between-study variance Tau-squared = **0.0026**

Test of ES=0 : z= **58.32** p = **0.000**

Supplementary Figure 90 – Pooled accuracy for diagnosing breast cancer on ultrasound

Study	ES	[95% Conf.	Interval]	% Weight
Byra et al. 2019 (a)	**0.860**	**0.804**	**0.916**	**7.09**
Byra et al. 2019 (b)	**0.840**	**0.784**	**0.896**	**7.05**
Byra et al. 2019 (c)	**0.830**	**0.756**	**0.904**	**6.12**
Cao et al. 2019	**0.730**	**0.677**	**0.783**	**7.23**
Cheng et al. 2016 (a	**0.824**	**0.791**	**0.857**	**8.20**
Chiao et al. 2019	**0.850**	**0.760**	**0.940**	**5.31**
Choi et al. 2019	**0.921**	**0.888**	**0.954**	**8.18**
Ciritsis et al. 2019	**0.871**	**0.806**	**0.936**	**6.56**
Ciritsis et al. 2019	**0.930**	**0.854**	**1.006**	**5.98**
Fujioka et al. 2019	**0.925**	**0.878**	**0.972**	**7.52**
Hizukuri et al. 2018	**0.876**	**0.830**	**0.922**	**7.56**
Lin et al. 2014	**0.923**	**0.858**	**0.988**	**6.59**
Qi et al. 2019	**0.935**	**0.922**	**0.948**	**8.82**
Xiao et al. 2019	**0.894**	**0.852**	**0.936**	**7.78**
D+L pooled ES	**0.873**	**0.841**	**0.906**	**100.00**

Heterogeneity chi-squared = **104.34** (d.f. = **13**) p = **0.000**
I-squared (variation in ES attributable to heterogeneity) = **87.5%**
Estimate of between-study variance Tau-squared = **0.0031**

Test of ES=0 : z= **52.81** p = **0.000**

Supplementary Figure 91 – Pooled F1 score for diagnosing breast cancer on ultrasound

Study	ES	[95% Conf. Interval]		% Weight
Cao et al. 2019	**0.740**	**0.688**	**0.792**	**16.04**
Cao et al. 2019	**0.794**	**0.735**	**0.852**	**15.36**
Tao et al. 2019	**0.908**	**0.872**	**0.943**	**17.60**
Xiao et al. 2019	**0.870**	**0.824**	**0.916**	**16.66**
Yap et al. 2018 (a)	**0.910**	**0.878**	**0.942**	**17.89**
Yap et al. 2018 (b)	**0.890**	**0.842**	**0.938**	**16.45**
D+L pooled ES	**0.855**	**0.803**	**0.906**	**100.00**

Heterogeneity chi-squared = **41.29** (d.f. = **5**) p = **0.000**
I-squared (variation in ES attributable to heterogeneity) = **87.9%**
Estimate of between-study variance Tau-squared = **0.0035**

Test of ES=0 : z= **32.73** p = **0.000**

Supplementary Figure 92 – Pooled PPV for diagnosing breast cancer on ultrasound

Study	ES	[95% Conf.	Interval]	% Weight
Cao et al. 2019	**0.690**	**0.635**	**0.745**	**15.14**
Cheng et al. 2016 (a	**0.822**	**0.789**	**0.855**	**15.94**
Choi et al. 2019	**0.895**	**0.857**	**0.933**	**15.79**
Hizukuri et al. 2018	**0.930**	**0.894**	**0.966**	**15.85**
Lin et al. 2014	**0.939**	**0.881**	**0.997**	**15.01**
Stoffel et al. 2018	**0.500**	**0.329**	**0.671**	**9.02**
Yap et al. 2019	**0.704**	**0.612**	**0.796**	**13.25**
D+L pooled ES	**0.804**	**0.727**	**0.880**	**100.00**

Heterogeneity chi-squared = **94.60** (d.f. = **6**) p = **0.000**

I-squared (variation in ES attributable to heterogeneity) = **93.7%**

Estimate of between-study variance Tau-squared = **0.0092**

Test of ES=0 : z= **20.65** p = **0.000**

Supplementary Figure 93 – Pooled NPV for diagnosing breast cancer on ultrasound

Study	ES	[95% Conf.	Interval]	% Weight
Cheng et al. 2016 (a	**0.834**	**0.802**	**0.866**	**20.43**
Choi et al. 2019	**0.932**	**0.901**	**0.963**	**20.48**
Hizukuri et al. 2018	**0.931**	**0.895**	**0.967**	**20.22**
Lin et al. 2014	**0.906**	**0.835**	**0.977**	**17.53**
Stoffel et al. 2018	**1.000**	**1.000**	**1.000**	**21.33**
D+L pooled ES	**0.922**	**0.851**	**0.992**	**100.00**

Heterogeneity chi-squared = **143.05** (d.f. = **4**) p = **0.000**
I-squared (variation in ES attributable to heterogeneity) = **97.2%**
Estimate of between-study variance Tau-squared = **0.0060**

Test of ES=0 : z= **25.70** p = **0.000**

Supplementary Figure 94 – Pooled AUC for diagnosing breast cancer on MRI

Study	ES	[95% Conf.	Interval]	% Weight
Antropova et al. 201	**0.890**	**0.867**	**0.913**	**27.96**
Antropova et al. 201	**0.880**	**0.826**	**0.934**	**9.05**
Antropova et al. 201	**0.880**	**0.826**	**0.934**	**9.21**
Dalmis et al. 2019	**0.852**	**0.823**	**0.881**	**22.22**
Herent et al. 2019	**0.816**	**0.757**	**0.875**	**7.94**
Truhn et al. 2018	**0.880**	**0.824**	**0.936**	**8.55**
Zhou et al. 2019	**0.858**	**0.819**	**0.897**	**15.07**
D+L pooled ES	**0.868**	**0.850**	**0.886**	**100.00**

Heterogeneity chi-squared = **8.31** (d.f. = **6**) p = **0.216**
I-squared (variation in ES attributable to heterogeneity) = **27.8%**
Estimate of between-study variance Tau-squared = **0.0002**

Test of ES=0 : z= **95.08** p = **0.000**

Supplementary Figure 95 – Pooled sensitivity for diagnosing breast cancer on MRI

Study	ES	[95% Conf.	Interval]	% Weight
Antropova et al. 201	**0.800**	**0.734**	**0.866**	**26.11**
Dalmis et al. 2018	**0.643**	**0.527**	**0.758**	**18.49**
Truhn et al. 2018	**0.783**	**0.712**	**0.854**	**25.30**
Zhou et al. 2019	**0.864**	**0.826**	**0.902**	**30.09**
D+L pooled ES	**0.786**	**0.710**	**0.861**	**100.00**

Heterogeneity chi-squared = **15.39** (d.f. = **3**) p = **0.002**
I-squared (variation in ES attributable to heterogeneity) = **80.5%**
Estimate of between-study variance Tau-squared = **0.0046**

Test of ES=0 : z= **20.39** p = **0.000**

Supplementary Figure 96 – Pooled specificity for diagnosing breast cancer on MRI

Study	ES	[95% Conf.	Interval]	% Weight
Antropova et al. 201	**0.820**	**0.757**	**0.883**	**32.64**
Truhn et al. 2018	**0.846**	**0.784**	**0.908**	**32.82**
Zhou et al. 2019	**0.703**	**0.652**	**0.754**	**34.54**
D+L pooled ES	**0.788**	**0.697**	**0.880**	**100.00**

Heterogeneity chi-squared = **14.53** (d.f. = **2**) p = **0.001**
I-squared (variation in ES attributable to heterogeneity) = **86.2%**
Estimate of between-study variance Tau-squared = **0.0056**

Test of ES=0 : z= **16.89** p = **0.000**

Supplementary Figure 97 – Pooled AUC for diagnosing breast cancer on digital breast tomosynthesis

Study	ES	[95% Conf.	Interval]	% Weight
Fan et al. 2019	**0.960**	**0.932**	**0.988**	**22.62**
Li et al. 2019 (a)	**0.917**	**0.904**	**0.930**	**27.57**
Mendel et al. 2018 (	**0.890**	**0.821**	**0.959**	**10.75**
Samala et al. 2016	**0.900**	**0.839**	**0.961**	**12.65**
Samala et al. 2018	**0.900**	**0.839**	**0.961**	**12.65**
Samala et al. 2019	**0.820**	**0.742**	**0.898**	**9.29**
Yousefi et al. 2018	**0.870**	**0.745**	**0.995**	**4.48**
D+L pooled ES	**0.908**	**0.880**	**0.937**	**100.00**

Heterogeneity chi-squared = **16.32** (d.f. = **6**) p = **0.012**
I-squared (variation in ES attributable to heterogeneity) = **63.2%**
Estimate of between-study variance Tau-squared = **0.0007**

Test of ES=0 : z= **62.08** p = **0.000**

Supplementary Figure 98 – Pooled sensitivity for diagnosing breast cancer on digital breast tomosynthesis

Study	ES	[95% Conf.	Interval]	% Weight
Fan et al. 2019	**0.900**	**0.856**	**0.944**	**25.66**
Li et al. 2019 (a)	**0.659**	**0.637**	**0.681**	**26.05**
Samala et al. 2016	**0.866**	**0.797**	**0.935**	**24.92**
Yousefi et al. 2018	**0.910**	**0.804**	**1.016**	**23.37**
D+L pooled ES	**0.831**	**0.675**	**0.988**	**100.00**

Heterogeneity chi-squared = **125.66** (d.f. = **3**) p = **0.000**
I-squared (variation in ES attributable to heterogeneity) = **97.6%**
Estimate of between-study variance Tau-squared = **0.0243**

Test of ES=0 : z= **10.41** p = **0.000**

Supplementary Figure 99 – Pooled accuracy for diagnosing breast cancer on digital breast tomosynthesis

Study	ES	[95% Conf.	Interval]	% Weight
Bevilacqua et al. 20	**0.920**	**0.835**	**1.005**	**2.09**
Li et al. 2019 (a)	**0.918**	**0.906**	**0.930**	**96.95**
Yousefi et al. 2018	**0.868**	**0.743**	**0.993**	**0.96**
D+L pooled ES	**0.918**	**0.905**	**0.930**	**100.00**

Heterogeneity chi-squared = **0.61** (d.f. = **2**) p = **0.738**
I-squared (variation in ES attributable to heterogeneity) = **0.0%**
Estimate of between-study variance Tau-squared = **0.0000**

Test of ES=0 : z= **146.25** p = **0.000**
